# NETosis associates with human TB lung tissue destruction and disease pathogenesis

**DOI:** 10.1038/s44321-026-00435-3

**Published:** 2026-06-02

**Authors:** Kimone L Fisher, Thabo Mpotje, Kievershen Nargan, Denelle Moodley, Kerishka Rajkumar-Bhugeloo, Omolara O Baiyegunhi, Threnesan Naidoo, Rajhmun Madansein, Mike Sathekge, Alasdair Leslie, Gillian Tomlinson, Gabriele Pollara, Mahdad Noursadeghi, Adrie J C Steyn, Thumbi Ndung’u, Mohlopheni J Marakalala

**Affiliations:** 1 Africa Health Research Institute, KwaZulu-Natal, South Africa; 2 School of Laboratory Medicine and Medical Sciences, University of KwaZulu-Natal, Durban, South Africa; 3 HIV Pathogenesis Programme, Doris Duke Medical Research Institute, Nelson R Mandela School of Medicine, University of KwaZulu-Natal, Durban, South Africa; 4 Department of Forensic & Legal Medicine, Walter Sisulu University, Mthatha, Eastern Cape South Africa; 5 Department of Cardiothoracic Surgery, University of KwaZulu Natal, Inkosi Albert Luthuli Central and King DinuZulu Hospitals, Durban, South Africa; 6 Nuclear Medicine Research Infrastructure (NuMeRI), Department of Nuclear Medicine, University of Pretoria and Steve Biko Academic Hospital, Pretoria, South Africa; 7 Division of Infection and Immunity, University College London, London, United Kingdom; 8 Clinical Medicine, School of Medicine and Population Health, University of Sheffield, Sheffield, United Kingdom; 9 Department of Microbiology, Centres for AIDS Research and Free Radical Biology, University of Alabama at Birmingham, Birmingham, AL USA; 10 South African Medical Research Council Centre for Tuberculosis Research, Division of Molecular Biology and Human Genetics, Faculty of Medicine and Health Sciences, Stellenbosch University, Cape Town, South Africa

**Keywords:** Immunology, Microbiology, Virology & Host Pathogen Interaction

## Abstract

Understanding drivers of tuberculosis (TB) associated lung pathological damage is vital in identifying targets for host directed therapies (HDT). NETosis is a neutrophil specific cell death characterized by release of neutrophil extracellular traps (NETs). The role of NETosis in TB-associated lung damage and disease pathogenesis is still poorly understood. We analysed human lung TB granuloma samples using a proteomics approach, which revealed enrichment of neutrophil-associated proteins in necrotic regions of caseous and cavitary granulomas. Using immunohistochemistry (IHC), we validated the abundance of neutrophil-associated proteins, including myeloperoxidase (MPO), cytochrome b-245 beta chain (CYBB) and neutrophil cytosolic factor 1(NCF1), as well as NETosis markers, neutrophil elastase (NE) and citrullinated H3, in necrotizing caseum of human TB granulomas. MPO protein expression was also more abundant in the plasma of TB patients compared to healthy and latently infected (LTBI) participants. MPO directly correlated with an inflammatory disease marker, IP-10. In addition, MPO and IP-10 colocalized in caseous lesions. In-vitro drug inhibition assays were used to investigate potential drivers of NETosis, with pharmaceutical inhibition of MPO, NE and CYBB resulting in reduction of NETosis induced by *Mycobacterium tuberculosis* (Mtb). Using RT-qPCR we analysed the expression of 18 neutrophil associated genes in the blood of healthy (n = 20), latent TB infection (LTBI) (n = 20) and TB (n = 30) participants. We found that MPO, NCF1 and NCF2 were upregulated in the TB group. Furthermore, the NETosis-associated genes were induced in a human standardized antigen challenge model. Our data shows evidence of NETosis as an associate of lung pathological damage in TB and identifies key drivers of the neutrophil cell death that can be intercepted as potential HDT targets to reduce neutrophil driven lung pathological damage.

## Synopsis

Understanding the role of neutrophils in TB lung pathological damage remains vital in determining possible targets for host directed therapies.

- Neutrophil-related proteins associate with increasing pathological damage in human lung tissues.
- NETosis associated proteins spatially localize with tissue destruction in necrotizing granulomas.
- In a human in-vivo antigen challenge model, NETosis related genes are more induced in people with active TB.
- Targeting neutrophil specific proteins, including neutrophil elastase and myeloperoxidase, with pharmaceutical inhibitors reduce the level of MTb-induced NETosis in-vitro.

## Abstract (web summary)

Understanding the role of neutrophils in TB lung pathological damage remains vital in30 determining possible targets for host directed therapies.

## Graphical abstract

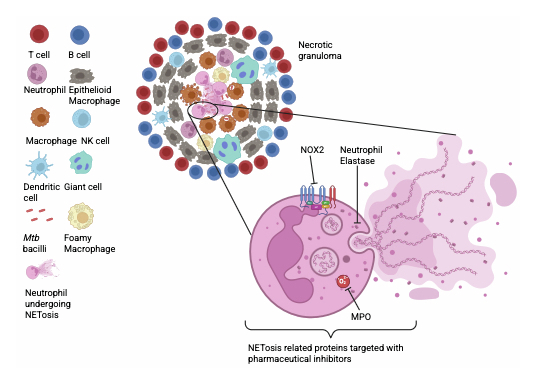

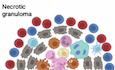

The paper explained **Problem**

Delineating the factors that contribute to Mtb pathological lung damage in humans is vital in identifying potential targets for host-directed therapies. Although neutrophils are well known to play a role in TB, their role in tissue damage and especially within granulomas, is not fully understood.

**Results**

In this study, we demonstrated via immunohistochemistry and immunofluorescence that NETosis-specific proteins were present in various necrotic granulomas. In addition, we report that NETosis-specific genes are upregulated in those with TB and that this response is replicated in a standardized human antigen challenge model. Mechanistically, pharmaceutical inhibition of NETosis-specific pathways such as myeloperoxidase, NADPH-associated NOX2 and neutrophil elastase, reduced the level of NETosis induced in vitro.

**Impact**

Our findings suggest new targets for host-directed therapy, an important aspect in discovering alternative therapies that address the lung damage associated with TB disease. In addition, a merit of this study is repurposing therapeutic drugs that are already approved for clinical trials, especially for those areas with a heavy disease burden that require economical solutions.

## Introduction

Tuberculosis (TB), a disease caused by *Mycobacterium tuberculosis* (Mtb), kills ~1.3 million people each year (World Health Organization. World Health Organization Tuberculosis Fact Sheet No. 104. Geneva: WHO; (2025)). Mtb infection induces a highly inflammatory response that results in the recruitment of innate immune cells to the site of infection (Cooper, 2009). However, the recruitment of cells, cytokines and proteases that initiate other anti-pathogenic activities may also exacerbate tissue damage (Fullerton and Gilroy, 2016; Elkington et al, 2011). Neutrophils are amongst the first cells recruited during infection (Eum et al, 2010). Neutrophilia is well known to be associated with TB pathology and clinical disease severity (Lowe et al, 2012). A recent report has utilized flow cytometry immunophenotyping to reveal diverse subsets of neutrophils in blood and airways during TB disease. The phenotypes included highly activated, proapoptotic and immunoregulatory neutrophil subsets, suggesting multiple mechanisms through which the cells may contribute to TB immunopathogenesis (Nhamoyebonde et al, 2024). Infection with Mtb is characterized by a disease spectrum that ranges from latent TB infection (LTBI) to subclinical and active TB (Pai et al, 2016). Recent work utilizing the Tuberculin Skin Test human challenge model has shown that individuals with active TB had exaggerated interleukin-17A (IL-17A) and T helper 17 (T_H_17) responses that were associated with increased neutrophil recruitment compared to latently infected individuals, suggesting a contribution of IL-17-associated neutrophils in TB pathogenesis (Pollara et al, 2021). Neutrophils form part of the granuloma, which is a multilayered immune structure comprised of various immune cells and is a pathological hallmark of TB (Marakalala et al, 2015; Kiran et al, 2015; Ndlovu and Marakalala, 2016). Although there is a spectrum of granuloma diversity, some of the common types of granulomas include: solid, caseous and cavitary granulomas. Solid granulomas are often well-organized and non-necrotic cellular structures. Caseous granulomas mainly have necrotic centers that are acellular, often surrounded by a cellular periphery. Early caseous granulomas may also be characterized by the presence of karyorrhectic debris at the centers. Some necrotizing caseous granulomas, exhibiting extensive central necrosis, may be characterized by liquefaction, frequently accompanied by cavitation (Marakalala et al, 2015; Sawyer et al, 2023; Gideon et al, 2022). When immune containment fails, TB-infected individuals progress to active TB, typically characterized by the presence of necrotizing caseum and lung cavitation (Ndlovu and Marakalala, 2016; Kim et al, 2010). Differentiating between these disease states is difficult and makes diagnosis and treatment challenging(Pai et al, 2016). Moreover, despite antibacterial treatment, the lung tissue destruction often observed in TB patients can pose serious long-term lung health defects (Ravimohan et al, 2017). Understanding the mechanisms that lead to tissue damage in TB pathogenesis remains vital in identifying host directed therapies (HDTs), to reduce lung pathology and add to adjunctive therapeutic strategies.

Neutrophils are the most abundant immune cells in the lung and are some of the initial cells infected with Mtb (Eum et al, 2010). In addition, neutrophils are attracted to necrotic lesions and make up a considerable portion of necrotic tissue within human tubercles, contributing to bacterial growth and survival (Lowe et al, 2012; Medlar, 1926).

We have previously used the MS-proteomics approach to show the presence of neutrophil-specific proteins in solid, caseous and cavitary granulomas (Marakalala et al, 2015). Among the proteins assessed, myeloperoxidase (MPO), cytochrome-b alpha/beta (CYBA/CYBB), neutrophil cytosol factors 1, 2 and 4 (NCF1, NCF2, and NCF4), and alarmins such as S100A8/9/12 were highly abundant in caseous and cavitary granulomas (Marakalala et al, 2015). These proteins exhibit an association with a mechanism of cell death known as NETosis. NETosis is characterized by the release of decondensed chromatin, which forms neutrophil extracellular traps (NETs) and is accompanied by the release of various other neutrophil antimicrobial components (Brinkmann et al, 2004). NETs specific proteins are associated with damaging pathology in respiratory infections such as influenza pneumonitis and COVID-19 (Veras et al, 2020; Narasaraju et al, 2010), and are used as biomarkers for RSV Bronchiolitis and sepsis (Sebina and Phipps, 2020; Margraf et al, 2008). These net-like structures trap and kill invading pathogens (Margraf et al, 2008; Ramos-Kichik et al, 2009; Urban et al, 2006; Guimarães-Costa et al, 2009). However, the release of potent microbicidal contents, such as histone-bound DNA, can result in cytotoxic activity mediated by NETs, causing tissue destruction (Saffarzadeh et al, 2011). Mtb is known to induce NETs, however the induced NET response does not eradicate the bacteria (Ramos-Kichik et al, 2009). In addition, Mtb infection promotes reactive oxygen species (ROS) driven necrosis of neutrophils and promotes Mtb growth. Necrosis of neutrophils and the resulting release of granular content may lead to tissue destruction in TB (Dallenga et al, 2017). NETosis-specific proteins have been described in various necrotic regions in different tissues, such as in abdominal aorta aneurysms [as reviewed by (Fernández-Ruiz, 2018)] and have been associated with acute lung injury in mice (Saffarzadeh et al, 2011). NETs are abundant in inflammatory regions and mediate tissue damage in other diseases (Papayannopoulos and Zychlinsky, 2009; Remijsen et al, 2011; Fuchs et al, 2007). Plasma NETs have been associated with TB disease, and neutrophil-derived metalloproteinase 8 (MMP8) is associated with TB immunopathology (Schechter et al, 2017; Ong et al, 2015). Recently, NETs have been reported in TB granulomas in C3HeB/FeJ mice, driven by type 1 interferon (IFN) response (Moreira-Teixeira et al, 2020a, 2020b).

Mechanisms that underpin human TB granuloma development and tissue destruction are poorly understood. Understanding how neutrophils and NETosis contribute to tissue destruction will aid the development of host-directed therapies to treat TB-associated lung damage. Here, we show that neutrophil-related proteins become more abundant with increasing pathological damage within human TB granulomas, and that NETosis markers spatially localize in the necrotizing regions of caseous granulomas. Targeting specific neutrophil genes resulted in the reduction of NETosis in a neutrophil in vitro model of Mtb infection. We report that the mediators of NETosis are associated with TB pathogenesis and that NETosis activity is induced to a greater degree in a human in vivo standardized antigen challenge model in people with active TB, indicating that elevated NETosis is a feature of TB disease.

## Results

### TB granuloma caseum is enriched with a neutrophillic protein signature

**Figure 1:**
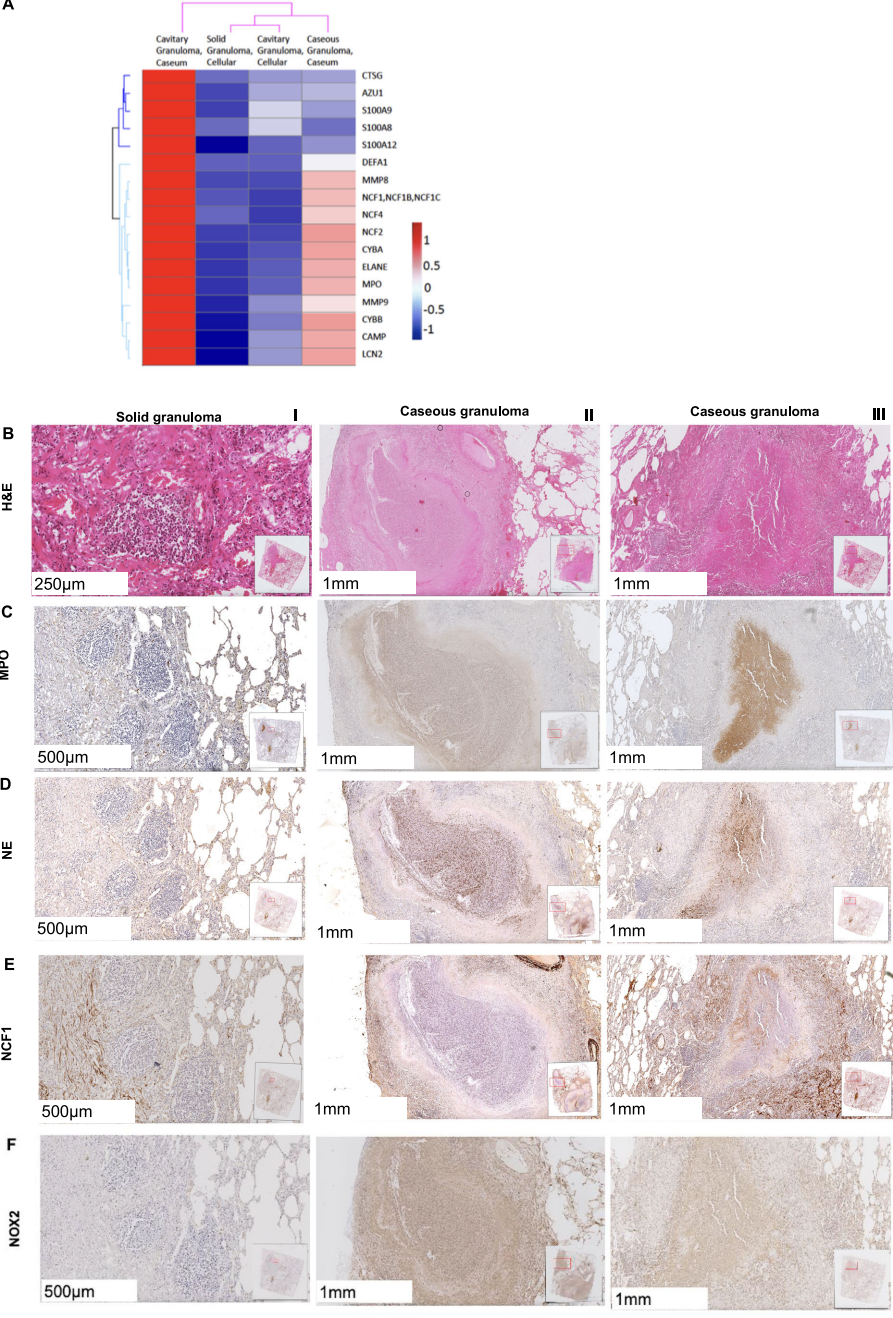
Neutrophil-specific proteins are associated with damaging pathology. (**A**) Heatmap of log2-transformed and z-scores of protein abundance in various granulomas. Unsupervised hierarchical clustering shows a higher relative abundance of neutrophil-specific proteins in caseous necrotic granulomas compared to solid/cellular granulomas. (**B**) Hemotoxylin and Eosin histology of solid granulomas with compact cellular clusters (i), early caseous granulomas with cellular debris at the center (ii), and necrotizing caseous granulomas displaying central necrosis (iii) and IHC staining for antibodies specific for (**C**) MPO, (**D**) ELANE (NE), (**E**) NCF1, and (**F**) NOX2. The counter stain (blue) stains the nuclei of cells, and the DAB (brown) stain is indicative of positive staining for the antibody of interest. Scale bar represents 250 μm to 1 mm indicated on each image. Images were viewed using NDP viewer 2 software. Source data are available online for this figure.

**Figure EV1:**
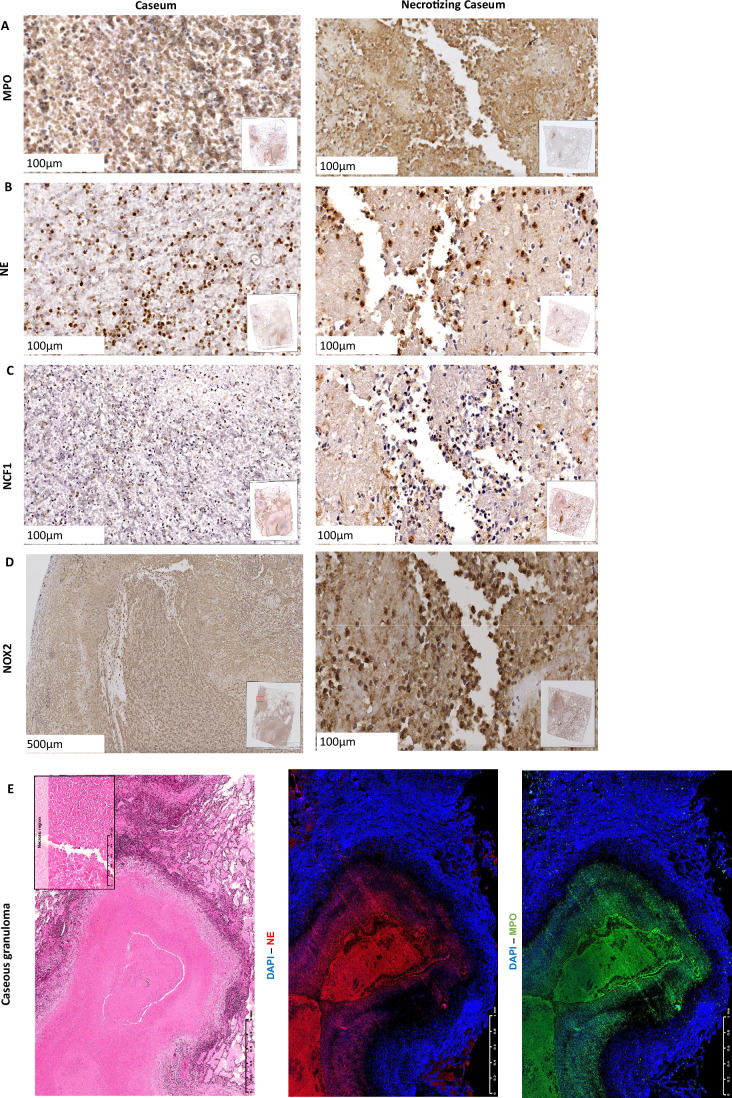
Neutrophil-specific proteins are enriched and colocalize in necrotic caseum. (**A**) MPO and (**B**) NE were abundant in early caseum (containing cellular debris) and necrotizing caseum. (**C**) NCF had a positive stain in caseum, which was more pronounced in necrotic regions. (**D**) NOX2 was more enriched in the borders of necrotizing caseum. IF staining of MPO (green) and NE (red) in the caseum (**E**). Scale bar represents 100, 500, and 1 mm.

We have previously profiled proteomic signatures in cellular and necrotic regions of various granulomas obtained from TB patients who had undergone surgical lung resections (Marakalala et al, 2015). Using the archived MS-proteomics datasets, we analyzed the expression patterns of neutrophil-associated proteins in the entire proteome. The proteins were not reported in our previous work. A number of neutrophil-related proteins, including Neutrophil MPO, Elastase (ELANE/NE), Neutrophil cytosol factors (NCF) 1, 2 and 4; CYBB (NOX2) and CYBA; and MMP8 and 9; were highly abundant in the necrotic regions of caseous and cavitary granulomas (Fig. 1A). To validate the protein abundance predicted by the MS-proteomics analysis, we performed IHC staining of cellular and caseous lesions. From HnE staining analysis, neutrophils were present throughout solid and caseous TB granulomas (Fig. 1B i–iii, respectively). We found that MPO, NE, NCF1, and NOX2 were highly enriched in the caseum compared to cellular regions (Fig. 1C–F, ii–iii, respectively). MPO and NE were specifically more abundant in necrotic regions of the caseum, which was further validated by IF staining of the proteins (Fig. EV1A,B,E). NCF1 also localized in regions of caseation, although in smaller amounts compared to MPO and NE (Fig. EV1C). NOX2 was present in both cellular and caseous granulomas, but largely abundant in the neutrophil enriched regions of the caseum (Figs. 1F ii and EV1D), suggesting a role for NADPH oxidase in a neutrophil mediated granuloma progression. Our data reveals an association of neutrophil-related proteins with the TB granuloma caseum.

### The neutrophil marker, MPO, correlates with inflammatory cytokines in plasma and in the caseous granulomas

**Figure 2:**
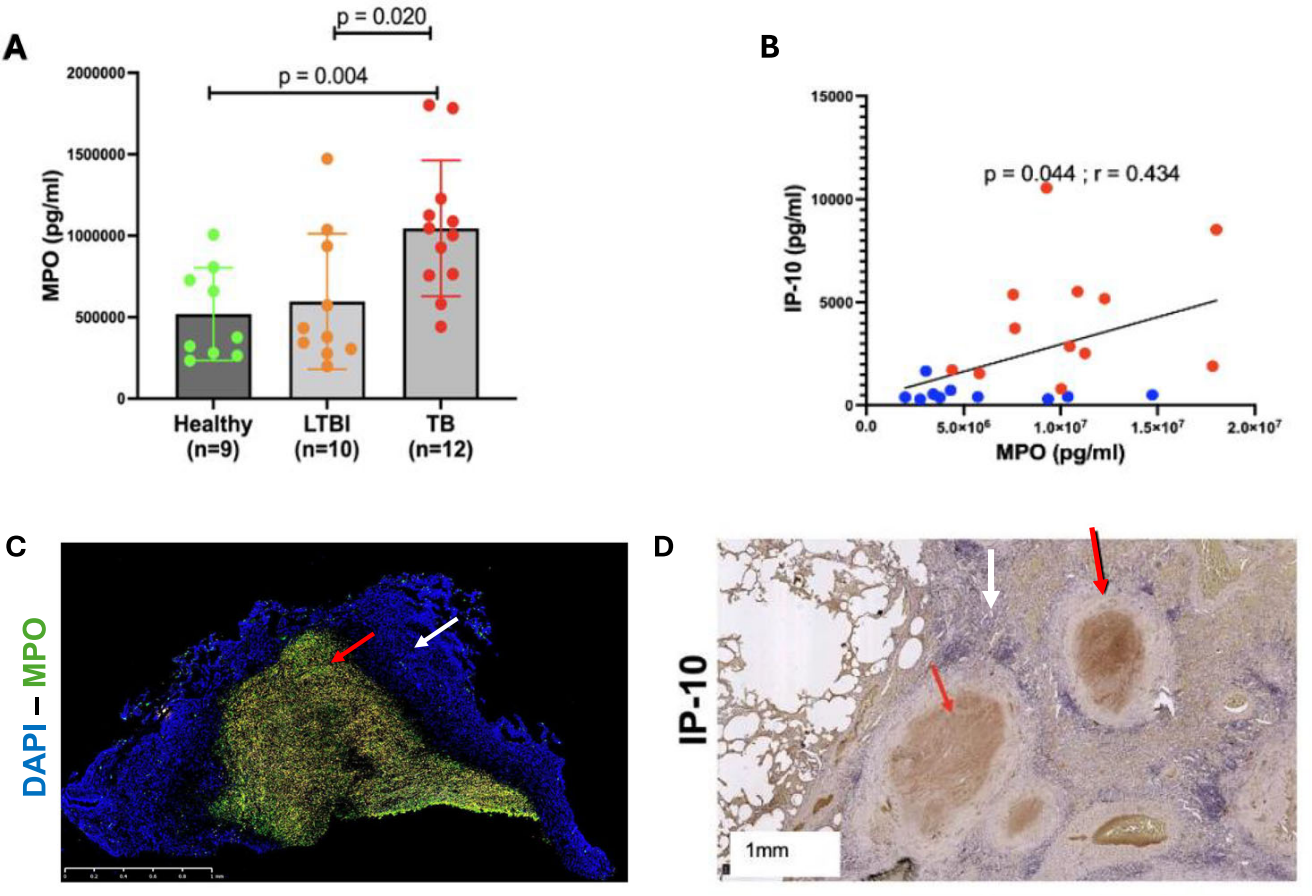
MPO correlation and colocalization with inflammatory cytokines in plasma and lung tissue. (**A**) MPO protein abundance in the healthy (*n* = 9), LTBI (*n* = 10), and TB (*n* = 12) plasma. Those in the TB arm had significantly higher inflammatory cytokine responses compared to those in the healthy arm (*p* = 0.004) and compared to those in the LTBI arm (*p* = 0.020). Data were analysed using unpaired *t*-tests and significant differences were defined as those values *p* < 0.05, with error bars representing the median and interquartile range. Post-hoc analysis was done using a Bonferroni adjustment to correct for multiple comparisons. (**B**) Direct correlation of MPO and IP-10 (Pearson correlation). Lung tissue sections were stained using IF and IHC to visualize the presence of (**C**) MPO and (**D**) IP-10, respectively, within granuloma regions, including cellular ring (white arrow) and necrotic core (red arrows). Scale bar for Fig. 2B, C represents 1 mm, as indicated on each image. Source data are available online for this figure.

Neutrophilia has been linked to TB disease severity and lung pathology (Lowe et al, 2013; Muefong and Sutherland, 2020). However, the role of neutrophils in TB granulomas remains poorly understood. Given the abundance of neutrophil-related proteins in the granuloma inflammatory regions, we sought to determine whether neutrophilia associates with established inflammatory markers of TB disease. Using ELISA, we measured MPO, a marker of neutrophil activation (Boettcher et al, 2020; Lau et al, 2005), in unstimulated plasma from healthy, LTBI and TB participants. MPO was significantly higher in TB patients compared to LTBI [TB (1045503 ±  417477 pg/ml) vs LTBI (595722 ± 416121 pg/ml), *p* = 0.020, Fig. 2], as well as in those with TB compared to those who were healthy [healthy (518505 ± 284226 pg/ml) vs TB (1045503 ± 417477 pg/ml), *p* = 0.004, Fig. 2A]. We then performed correlation analysis of MPO against 27 plasma immune mediators; cytokines, chemokines, and growth factors in the plasma of LTBI and TB individuals. We found that IP-10, an inflammatory chemokine that has been shown to be a biomarker of TB disease (Fisher et al, 2022a; Azzurri et al, 2005), directly correlated with MPO (*p* < 0.05, Fig. 2B), suggesting an association of neutrophilia with markers of TB-associated inflammation. To recapitulate this relationship in TB granulomas, we stained for IP-10 and analyzed its spatial abundance in the tissue. We found that IP-10 was abundant in the TB lung lesions and colocalized with MPO in the caseum (Fig. 2C,D). Our results suggest an association of neutrophil activation with TB pathogenesis.

### NETosis is more abundant in the caseum and localizes within necrotizing foci

**Figure 3:**
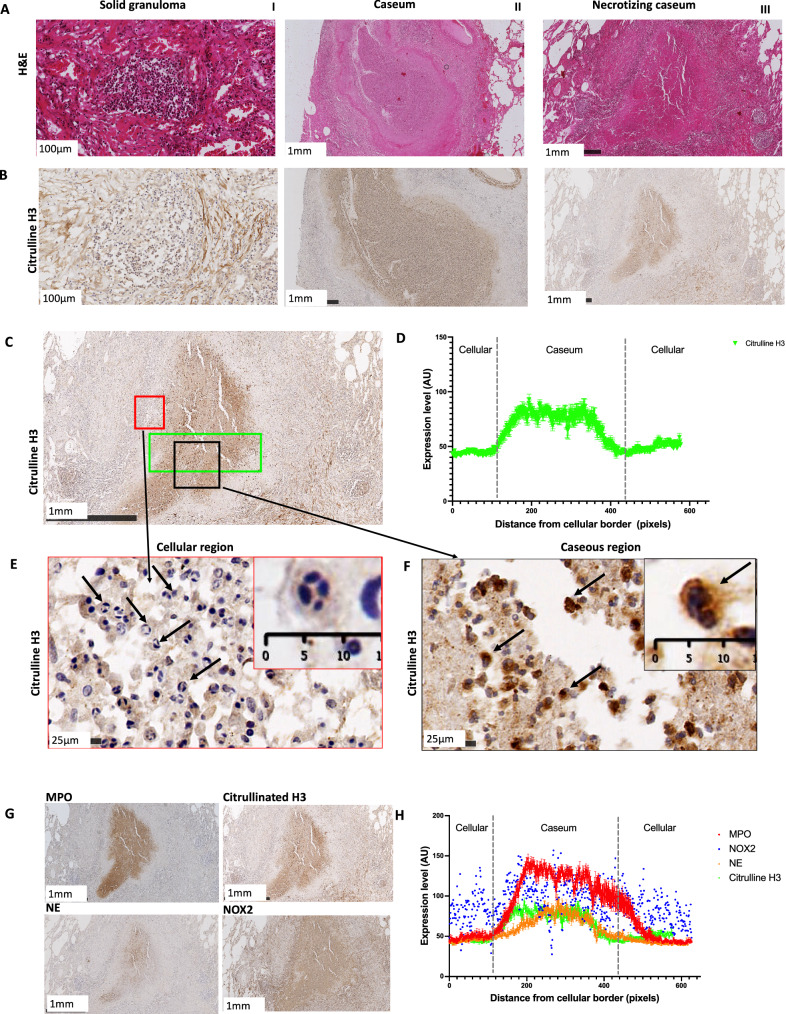
Immunohistochemistry (IHC) staining of NETosis proteins in human lung tissue microdissections. Lung tissue samples were stained with hemolysin and eosin (Fig. 3A i–iii, which is a reused panel from Fig. 1A), anti-citrulline H3 (Fig. 3B i–iii). Anti-citrulline H3 stained caseum with visible necrotic damage (Fig. 3C). Citrullinated H3 was abundant in the caseum compared to the cellular regions (Fig. 3C, green rectangle and Fig. 3D, green line, *n* = 3). Further magnification into the region outside the caseum illustrated by the red square (Fig. 3C) shows that in the cellular region neutrophils are intact and not extruding NETs, indicated by the black arrows and insert image (Fig. 3E which is a reused and magnified image of Fig. 3C). Citrullinated H3 is enriched in the necrotic region of the caseum (Fig. 3C, black square), with visible extrusion of NETs indicated as dark brown stain (Fig. 3F which is a reused and magnified image of Fig. 3C). Anti-MPO (red line), anti-NE (orange line), anti-citrullinated H3 (green line) and anti-NOX2 (blue line) show NETosis-specific proteins in necrotic granulomas and the abundance of these proteins in the necrotic caseum compared to cellular regions (Fig. 3G, H (*n* = 3)). Error bars represent the median and interquartile range. Figure 3G is reused from Fig. 1C, D, F. Scale bar represents 15 μm–1 mm, as indicated on each image. Source data are available online for this figure.

**Figure EV2:**
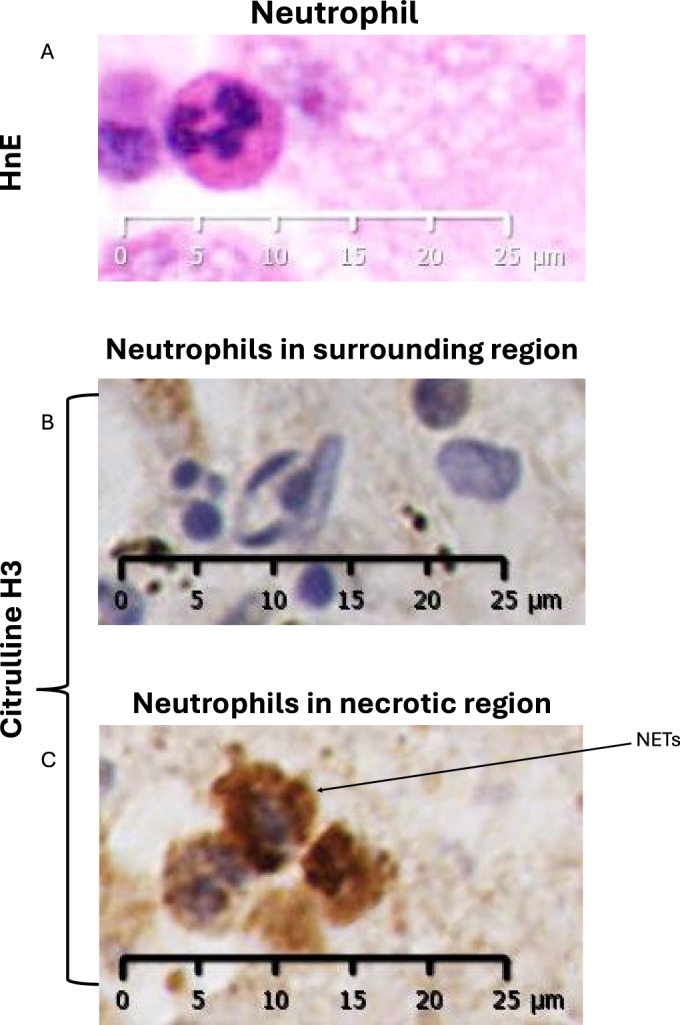
NETs are more abundant in the caseum than in cellular regions. Lung tissue samples were stained with hemolysin and eosin (**A**). IHC-stained tissue showing Citrulline H3-negative neutrophils in the cellular region of TB granuloma (**B**). IHC-stained tissue showing neutrophils extruding NETs in the caseum of TB granulomas (**C**). Scale bar = 25 μm.

Neutrophil degranulation proteins NE and MPO are known to be released with the extrusion of extracellular fibril matrix termed neutrophil extracellular traps (NETs)(Brinkmann et al, 2004; Zhu et al, 2021a). The abundance of these proteins in the TB granuloma caseum suggests enrichment of NETosis in these lesions. We stained various types of human TB granulomas, including solid granulomas with compact cellular clusters (Fig. 3A i), early caseous granulomas with karyorrhectic debris at the center (Fig. 3A ii), and necrotizing caseous granulomas displaying central necrosis (Fig. 3A iii) with an anti-citrulline H3 antibody, a marker of neutrophil extracellular trap (NET) formation(Mauracher et al, 2018). Our IHC analysis revealed a stronger staining intensity in caseum compared to solid granulomas (Fig. 3B), suggesting an association of NETosis with tissue destruction. Indeed, on higher magnification, NETosis was much more localized within necrotic regions of the caseum (Fig. 3C,D,F) than in neutrophils found on the cellular regions outside of the caseum (Fig. 3C–E). Interestingly, extrusion of citrulline H3-positive NETs was visible at the cellular scale among a number of individual neutrophils in the necrotizing caseum compared to individual citrulline H3-negative neutrophils in the cellular regions (Figs. 3E,F and EV2A–C). This suggests that in necrotizing tissue, as per the mechanism of action of NETosis, nuclear DNA is expelled out of the nucleus and into the extracellular regions. Both NE and MPO were more abundant in the region of the NETs in the caseous regions compared to cellular regions (Fig. 3G,H). Interestingly, NOX2 was also abundant in the regions of NETs release (Fig. 3G), suggesting that TB-induced NETosis in lung tissues may be NADPH oxidase dependent. Overall, these results suggest that NETosis associates with TB granuloma caseation.

### Neutrophil extracellular traps colocalize with neutrophil elastase in caseous granulomas

**Figure 4:**
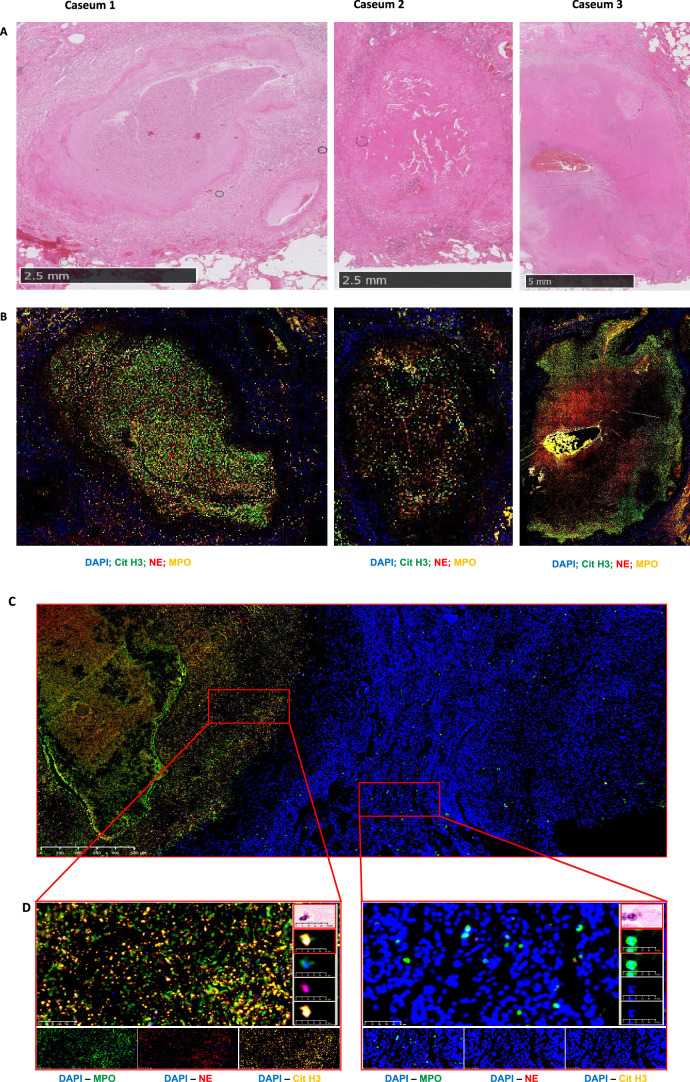
NE colocalizes with NETosis in multiple caseous granulomas. H&E staining of human TB-diseased lung illustrating various caseous granulomas, caseum 1, containing karyorrhectic debris at the center, and caseum 2 and 3, both containing acellular necrotic core. (**A**) Immunofluorescence staining showing citrulline H3 (green), NE (Red), and MPO (yellow) in various caseous granulomas. (**B**) Illustration of the spatial localization of citrullinated H3 (yellow), MPO (green), and NE (Red) within the necrotic center of a caseous granuloma. (**C**) Zoomed-in images showing IF colocalization of NE and citrulline H3 in the caseum and no colocalization in the cellular region of the same granuloma (Scale bars represent 25, 50, 500 μm, 2.5 mm and 5 mm. Source data are available online for this figure.

**Figure EV3:**
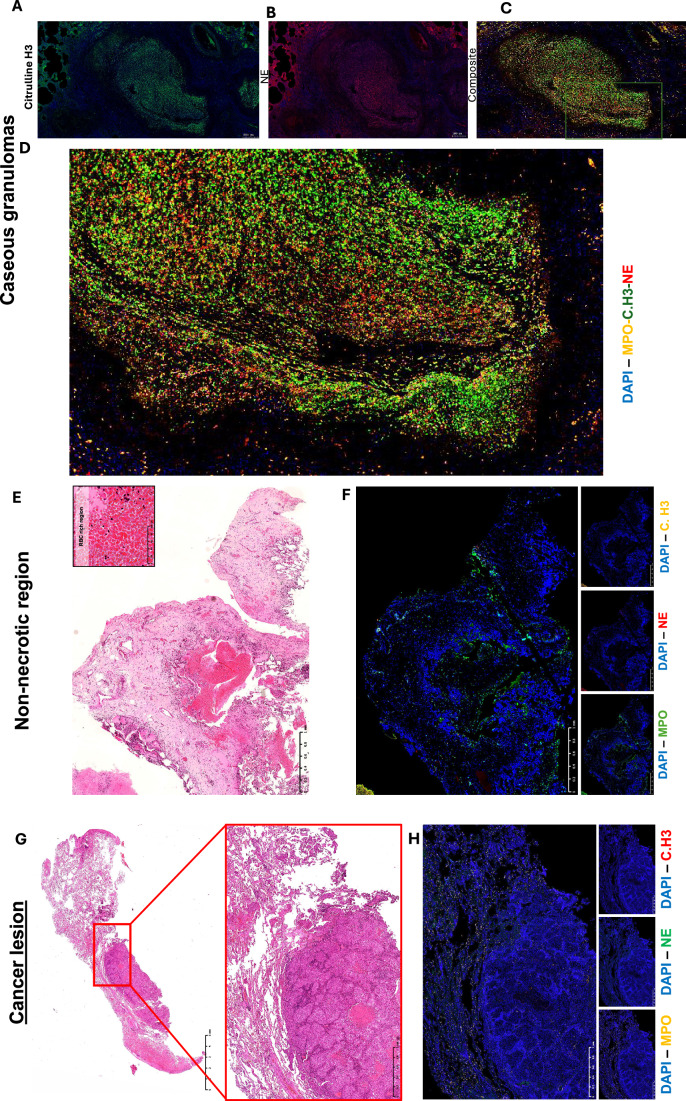
Neutrophil elastase colocalizes with NETs in the granuloma caseum. Immunofluorescence (IF) staining showing citrulline H3 (green) (**A**), NE (Red) (**B**), and composite citrulline H3, NE, and MPO (yellow) co-staining on the same caseous granuloma (**C**), which is zoomed in to show colocalization of NE and citrulline H3 (**D**). H&E staining of human TB lung illustrating a non-necrotic granuloma (**E**). IF staining of MPO (Green), NE (Red) and citrulline H3 (Yellow) on non-necrotic granuloma **(F)**. H&E staining of control human adenocarcinoma associated lung pathology (**G**). IF staining of the adenocarcinoma lesion showing MPO (Yellow), NE (Green), and citrulline H3 (Red). Scale bars represent 5 mm, 1 mm, 500 µm, and 50 µm as indicated on each image.

Neutrophil elastase (NE), known to possess proteolytic activity (Gramegna et al, 2017), is one of the major serine proteases released by neutrophils. In elevated amounts, NE is known to mediate extracellular matrix destruction by destroying the tight junctions between cells and increasing tissue permeability (Zhu et al, 2021b; Chua and Laurent, 2006). The abundance of NE with citrulline H3 (Figs. 3B and EV1E) in caseous granuloma regions suggests an interaction between NE and the released neutrophil chromatin. Potentially, NE could be involved in the degradation of the chromatin. To confirm this interaction, we sought to characterize the spatial association of NE and NETosis in the areas of tissue destruction in heterogeneous forms of TB caseous granulomas and non-necrotic lesions (Figs. 4A–C and EV3A,B,F). Using immunofluorescence imaging, we found that citrulline H3 was abundant in the caseous center of the granulomas, and consistently colocalized with NE in all the heterogeneous forms of the caseum we sampled (Figs. 4B,C and EV3C,D). In contrast, this colocalization was absent in non-necrotic granuloma (Fig. EV3E,F). The zoomed-in images indicated the striking association of the NE activity alongside NET release in the areas of tissue damage (Figs. 4C,D and EV3D). In contrast, only very little NE was found in the cellular region outside the caseum, with no evidence of colocalization with NETs (Figs. 4C,D and EV3D). Further cellular-level analysis showed that neutrophils (MPO-positive) within the caseous regions exhibited stronger co-localization of NE and citrulline H3 compared to those in the outer cellular areas (Fig. 4C,D), indicating increased NETosis activity in the caseous regions. Additionally, the co-localization of NE with citrullinated H3 was minimal in control cancer-associated necrotic lesions, suggesting that the heightened NETosis activity may be specifically driven by TB (Fig. EV3G,H). This data suggests that neutrophil-associated lung pathological damage in TB may be mediated by NETosis through NE, which is known to possesses a proteolytic activity linked to extracellular tissue matrix destruction (Chua and Laurent, 2006).

### TB-related stimuli induce NETosis in in vitro neutrophil model

**Figure EV4:**
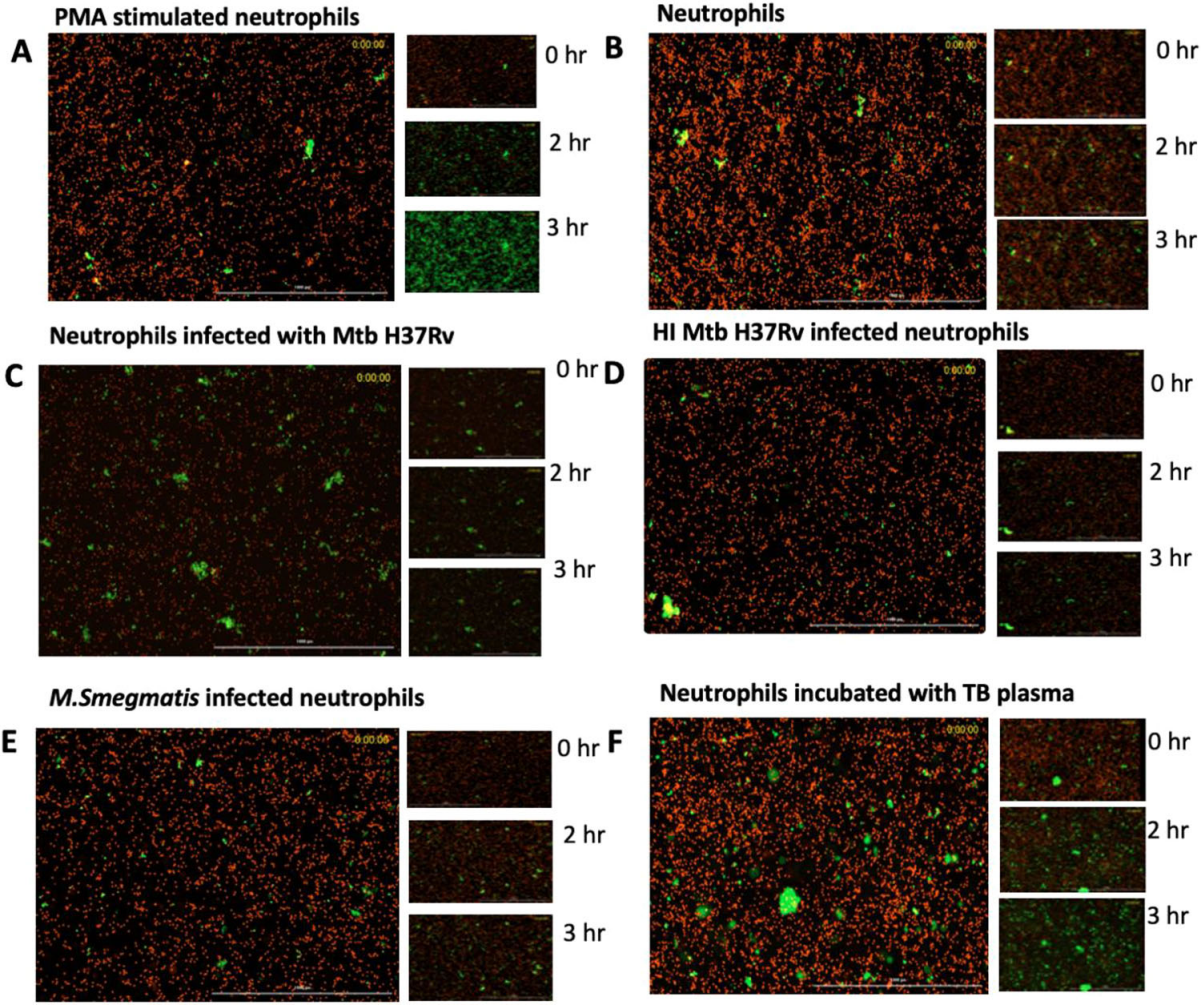
Pathogenicity and viability of Mtb H37RV impact NETosis. Neutrophils were stimulated/infected with (**A**) PMA stimulated neutrophils (*n* = 3), (**B**) neutrophils (*n* = 3), (**C**) neutrophils infected with Mtb H37Rv (*n* = 3), (**D**) neutrophils infected with Heat-Inactivated Mtb H37Rv (*n* = 3), (**E**) neutrophils infected with *M. smegmatis* (*n* = 3), and (**F**) Neutrophils incubated with TB plasma (*n* = 3). The indicated scale represents magnification with the Biotek® cytation 5 Gen 5 software at 1000 μm. Green fluorescence at an excitation/emission of 503/526 nm was detected with GFP filter, and Permeable nuclear red at 622/645 nm was detected with Cy5 filter using the Biotek® Cytation 5 cell imaging multi-mode reader. Images were captured every 30 min for 6 h and represent a single section within each well.

To evaluate the identified proteins as potential mediators of TB-induced NETosis, we sought to optimize a model that enables temporal characterization of the neutrophil cell death during the course of Mtb infection. We utilized live imaging microscopy in an in vitro model of neutrophil infection. Neutrophils were isolated from healthy participants and stimulated with various stimuli. We found that PMA, well known to be a strong inducer of NETosis, resulted in complete NETs formation over the 6 h incubation period (Fig. EV4A). Compared to unstimulated neutrophils that exhibited low levels of NETosis (Fig. EV4B), neutrophils stimulated with Mtb H37Rv extruded more NETs (Fig. EV4C). This is consistent with a previous report that showed Mtb as an inducer of NETosis (Dallenga et al, 2017). Stimulation with heat-killed/inactivated (HI) Mtb resulted in lower levels of NETs formation compared to the stimulation by live Mtb, suggesting the importance of viability in NETosis induction (Fig. EV4D). Similarly, we stimulated neutrophils with *M. smegmatis*, a rapidly growing mycobacterial bacilli not considered pathogenic in humans, and there was very little NETosis triggered by *M. smegmatis* stimulation compared to Mtb (Fig. EV4E), suggesting that mycobacterial pathogenicity is associated with pronounced neutrophilic cell death. Previous studies have shown that cytokines present in plasma may induce NETosis in diseases such as atherosclerosis and COVID-19 (Romano et al, 2022; An et al, 2019). Given the association of neutrophilia with plasma cytokines in TB participants (Fig. 2B), we hypothesized that the TB plasma could contain mediators of NETosis. Indeed, stimulation of neutrophils with TB plasma resulted in a high proportion of NETotic events (Fig. EV4F). These data show that NETosis is a TB-induced process in vitro and that mycobacterial pathogenicity and viability likely play an important role in the induction of the cell death. Our results also suggest that TB plasma inflammatory markers may be drivers of NETosis.

### Pharmaceutical inhibition of NE, MPO and NOX2/CYBB reduces Mtb-induced NETosis

**Figure 5:**
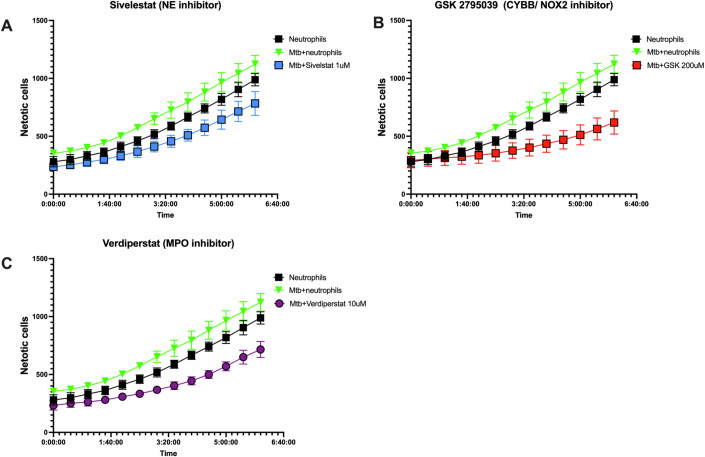
Interception of NE, MPO and NOX2 reduces NETosis induced by Mtb H37Rv infection of healthy neutrophils. Neutrophils were treated with specific pharmaceutical inhibitors at time point 0 to determine whether NETosis induced by Mtb infection could potentially be reduced by HDTs. In each condition, (**A**) Mtb H37Rv-infected neutrophils treated with neutrophil elastase inhibitor Sivelestat 1μM (blue square, *n* = 3), (**B**) NOX2/CYBB inhibitor GSK2795039 at 200 μM (red square, *n* = 3), and (**C**) MPO inhibitor Verdiperstat at 10 μM (purple circle, *n* = 3) significantly reduced NETosis at the 6 h mark, compared to the Mtb H37Rv-infected neutrophil groups (*p* < 0.05). The presence of NETosis was lower compared to those neutrophils that were not treated, from time point 0–6 h in each inhibitor condition. Kinetic imaging was performed for a duration of 6 h, at 30 min intervals. Images were captured at a 1000 μm scale and represent a single section within each well. Repeated measures ANOVA were used to compare each time point and each treatment condition and control; significant results were defined as those with *p* < 0.05, with error bars representing 95% confidence interval. Source data are available online for this figure.

Degranulation is a neutrophil cellular feature that marks early events leading to the extrusion of NETs. This process involves fusion of granules with the plasma membrane, resulting in extracellular release of granule proteins (Lacy, 2006; Othman et al, 2022). To determine whether TB-mediated NETosis could be pharmaceutically targeted, we utilized inhibitors of the degranulation proteins (Fig. 1A), that showed strong association with NETs formation in the necrotic caseum, namely NE, NOX2/CYBB, and MPO. Using automated microscopy, we quantified NETosis in Mtb-infected neutrophils using kinetic imaging over a period of 6 h, for each specific pharmaceutical inhibitor. Compared to untreated Mtb-infected neutrophils, treatment with Sivelestat (NE inhibitor) at 1 mM (Fig. 5A), GSK2795039 (NOX2/CYBB inhibitor) at 200 μM (Fig. 5B) and Verdiperstat (MPO inhibitor) at 10 μM (Fig. 5C) significantly reduced NETosis after 6 h of incubation with each inhibitor (*p* < 0.05). Overall, these results indicate that NE, CYBB and MPO are mediators of TB-induced NETosis and that their downregulation may provide a therapeutic strategy to ameliorate neutrophil-associated pathology.

### Neutrophilic gene signatures, including drivers of NETosis, are upregulated in TB patients

**Figure EV5:**
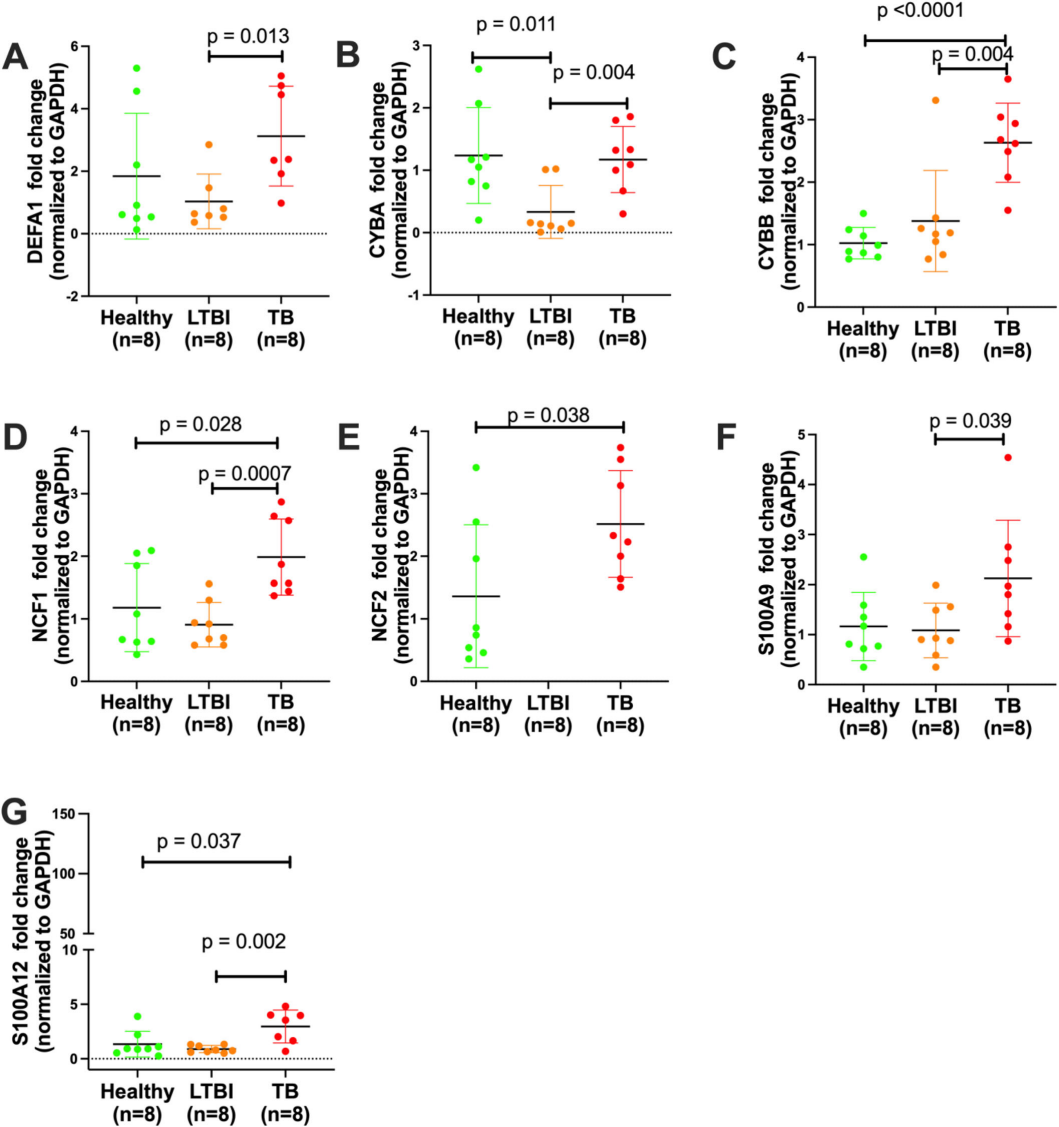
Gene expression analysis of candidate genes in healthy (*n* = 8), LTBI (*n* = 8), and TB (*n* = 8) participants. (**A**) DEFA1 was significantly upregulated in the TB arm compared to the LTBI arm (*p* = 0.013), (**B**) CYBA was more upregulated in the healthy arm compared to the LTBI arm (*p* = 0.011) and in the TB arm compared to the LTBI arm (*p* = 0.004), (**C**) CYBB was upregulated in the TB arm compared to the healthy arm (*p* < 0.0001) and LTBI arm (*p* = 0.004), (**D**) NCF1 was upregulated in the TB arm compared to the healthy arm (*p* = 0.028) and the LTBI arm (*p* = 0.0007), (**E**) NCF2 was more upregulated in the TB arm compared to the healthy arm (*p* = 0.038), (**F**) S100A9 was more upregulated in the TB arm compared to the LTBI arm (*p* = 0.039) and (**G**) S100A12 was upregulated in the TB arm compared to the healthy arm (*p* = 0.037) arm and LTBI arm (*p* = 0.002). All gene expression values were normalized to GAPDH using the 2ΔΔ^ct^ method. Unpaired *t*-tests were used to determine significant (*p* < 0.05) differences between groups.

**Figure 6:**
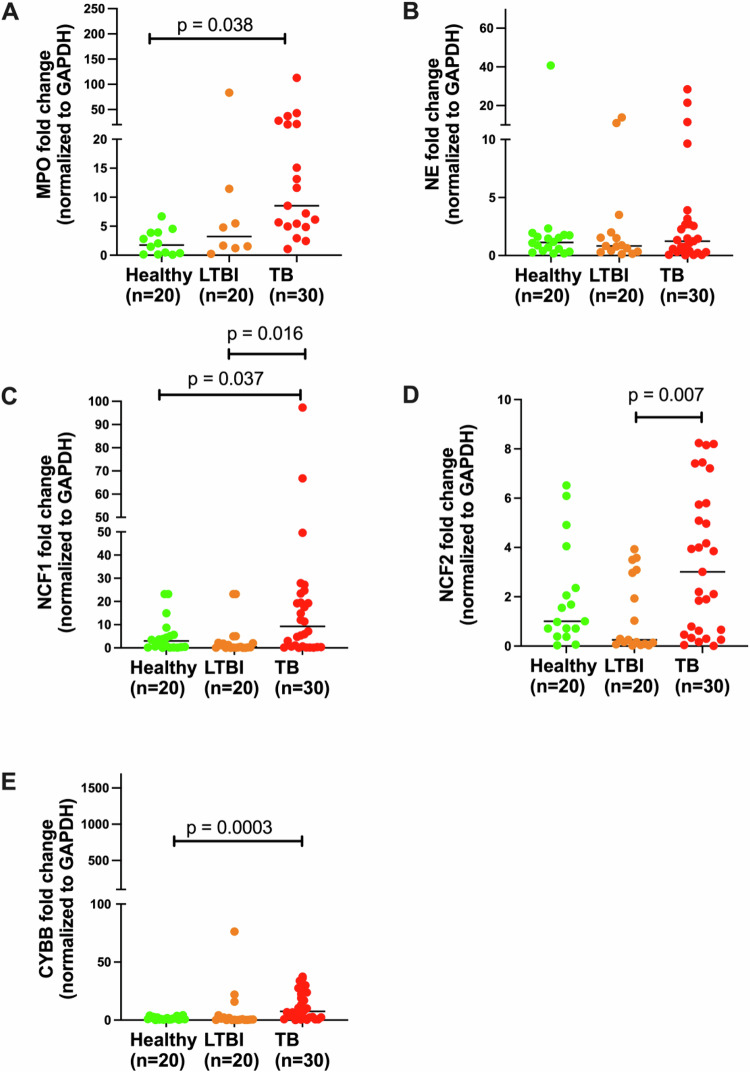
Gene expression of NETosis-associated genes. Gene expression of (**A**) Myeloperoxidase (MPO), (**B**) NE, (**C**) NCF1, (**D**) NCF2, and (**E**) CYBB were analysed in the TB group (*n* = 30), LTBI (*n* = 20) and the healthy controls (*n* = 20). The data were normalized to GAPDH, and the fold change was calculated using the 2ΔΔ^ct^ method. Data were analysed using unpaired *t*-tests. Significant results were defined as those *p* < 0.05. Source data are available online for this figure.

To determine the relevance of the NETosis mediated pathological damage in TB clinical disease, we investigated mRNA expression of the 18 neutrophil-associated genes (Table EV2), consistent with the protein signatures from our granuloma discovery proteomics (Fig. 1A). We performed qPCR using whole blood RNA from healthy (IGRA-), LTBI (IGRA+), and TB (GeneXpert+) participants (see Table 1 for details of participants). In our pilot screening using 24 participants, we found that DEFA1, CYBB, CYBA, NCF1, S100A9, and S100A12 were upregulated in the TB group compared to the LTBI group (*p* < 0.05, Fig. EV5A–D,F,G, respectively). In addition, CYBB, NCF1, NCF2, and S100A12 were upregulated in the TB group compared to the healthy group (*p* < 0.05, Fig. EV5C–E,G). To determine if this was true in a larger study group, we increased the sample size to 70 participants (20 healthy, 20 LTBI and 30 TB participants) and repeated the gene expression analysis on specific genes that were associated with NETosis-inducing pathways. Our data shows that MPO, CYBB and NCF1 were upregulated in the TB group compared to the healthy group (*p* < 0.05, Fig. 6A,C,E). NCF1 and NCF2 were upregulated in the TB group compared to the LTBI group (Fig. 6C,D). NE also appeared to be upregulated in the TB group, although the increasing trend was not significant (Fig. 6B). These results suggest that NETosis-associated genes are more upregulated in individuals with TB compared to those without the disease.

**Table 1:** Clinical characteristics of *n* = 70 participants used in this study.

Characteristics	Healthy (IGRA-)	LTBI (IGRA + )	TB (GeneXpert + )
*n* (70)	20	20	30
**Age (mean and SD)**	30.90 ± 12.59	32.00 ± 11.95	35.37 ± 10.93
**Sex**			
Male	35.% (7)	45% (9)	73.33% (22)
Female	65% (13)	55% (11)	23.33% (7)
Not disclosed			3.33% (1)
**Assays**			
plasma - Bio-Plex	9	10	12
qPCR on whole blood RNA	20	20	30
MPO ELISA	9	10	12

### NETosis-associated genes are upregulated in TB compared to LTBI in a human standardized antigen challenge model

**Figure 7:**
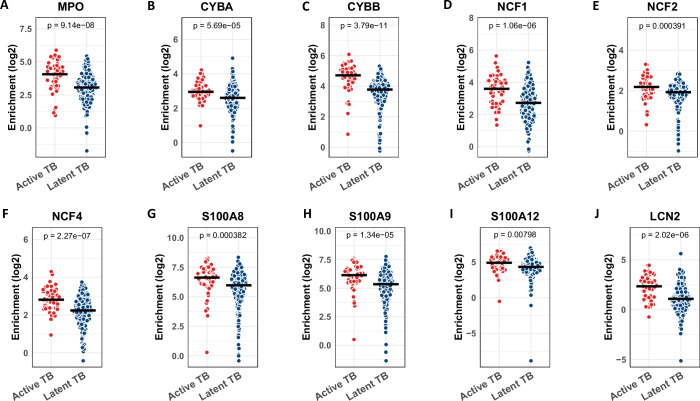
NETosis-associated genes are upregulated in response to TST challenge in TB patients. Gene expression of several netosis-specific genes were significantly upregulated in the active TB (*n* = 48) compared to latent TB (*n* = 191); (**A**) MPO (*p* = 9.14e^−08^), (**B**) CYBA (*p* = 5.69e^−05^), (**C**) CYBB (*p* = 3.79e^−11^), (**D**) NCF1(*p* = 1.06e^−06^), (**E**) NCF2 (*p* = 0.000391), (**F**) NCF4 (*p* = 2.27e^−07^), (**G**) S100A8 (*p* < 0.000382), (**H**) S100A9 (*p* = 1.34e^−05^), (**I**) S100A12 (*p* < 0.00798), and (**J**) LCN2 (*p* = 2.02e^−06^. Data were analysed using Mann–Whitney tests, and horizontal lines on scatter dot plots represent medians with 95% confidence interval. Significant results were defined as those with *p* < 0.05. Each dot represents one participant, with the TB group represented by red dots and the LTBI group represented by blue dots. Source data are available online for this figure.

Cross-sectional assessment of unstimulated blood, as well as comparisons between diseased and normal lung, is intrinsically confounded by the presence and duration of active TB. Therefore, these approaches alone cannot distinguish whether increased expression of NETosis-associated genes is a primary event in driving TB pathology or occurring solely in response to established disease. To address this issue, we made use of the human in vivo tuberculin skin test (TST) challenge model. Transcriptomic assessments of the site of TST permit standardized assessments between groups following tissue mycobacterial antigen stimulation (Bell et al, 2016; Pollara et al, 2021). Neutrophil infiltration is a feature of the TST response (Pollara et al, 2021), and we have extended this work by focusing on neutrophil NETosis-associated genes (Table EV2; Fig. 1A) that are induced in a TST response relative to control saline injection. Strikingly, many of these genes, including MPO, CYBA, CYBB, NCF1, NCF2, NCF4, S100A8, S100A9, S100A12, and LCN2, showed elevated expression in patients with active TB compared to LTBI controls (Fig. 7A–J). We conclude from these data that greater induction of neutrophil-associated NETosis genes is an intrinsic property of host-pathogen interactions in active TB.

## Discussion

In this study, we sought to investigate NETosis, as a contributor to pathological damage associated with TB. We found that NETosis-associated proteins are enriched in necrotizing caseum of human TB granulomas. In addition, targeting NETosis-associated genes reduced neutrophil cell death in in vitro infection model. Our study demonstrates clinical relevance as NETosis-specific genes were upregulated in the blood of individuals with active TB, and in response to TST challenge in TB patients.

Neutrophilia is well known to be associated with clinical TB disease severity (Lowe et al, 2012; Muefong and Sutherland, 2020). We show that the abundance of MPO protein in plasma was recapitulated in tissue pathology, confirming the role of neutrophils in clinical disease. IHC staining showed an abundance of NCF1, NOX2/CYBB, and NE (NETosis protein markers) in necrotizing granuloma and in the periphery of caseous granulomas, which validated neutrophil-associated protein signatures previously predicted to be enriched in caseous granulomas (Marakalala et al, 2015). Furthermore, the presence of NOX2 suggests involvement of NADPH oxidase in TB-induced NETotic events.

NETs serve as damage-associated molecular patterns and induce the production of proinflammatory cytokines (Warnatsch et al, 2015). We recently showed that IP-10 may serve as a biomarker of pulmonary TB disease (Fisher et al, 2022a). IP-10, which is an inflammatory chemokine that is secreted by monocytes (Liu et al, 2011), colocalized with MPO in the caseum and correlated with MPO protein abundance in TB plasma. These inflammatory cues within different regions of the caseum further support the cellular diversity observed within granulomas.

Interestingly, our data also shows that neutrophils that localize in the cellular ring exhibit an intact structure. This corroborates recent studies emphasizing the tissue heterogeneity observed in granuloma structures (Sawyer et al, 2023). We found that citrullinated H3 and NE were present in necrotizing granulomas. Citrullinated H3 is a potent antimicrobial agent that induces a caspase inflammasome response, resulting in tissue injury (Tian et al, 2021). In addition, both NE and citrullinated histones activate TLR4 and may lead to phagocytosis of the trapped bacteria via post-cytotoxic NETotic activity (Fuchs et al, 2007). Mtb induces NETosis through virulence factors located in the region of difference (RD1), which encodes the ESX-1 type VII secretion system. This system produces ESAT-6, a leukocidin that cripples the NADPH oxidase pathway, and causes calcium influx, thus triggering NETosis (Francis et al, 2014; Corleis et al, 2012). In a zebrafish model, neutrophils were shown to kill Mtb during the early stages of infection within the granuloma through the NADPH oxidase pathway, suggesting that the timing of these interactions is important (Yang et al, 2012). Our findings of NETosis protein markers in TB granulomas corroborate those recently reported, indicating the presence of citrullinated H3 in human and mouse granulomas (Moreira-Teixeira et al, 2020a). Our study differs in that we show localization of NETosis components within necrotizing caseum and its association with human TB pathogenesis in multiple disease compartments. In addition, we show a potential mechanism of cell death responsible for mediating tissue damage associated with TB pathology.

Our in vitro model of neutrophil infection indicated that NETosis is a TB-induced parameter, corroborating previous studies illustrating that Mtb induces NET formation (Ramos-Kichik et al, 2009; Filio-Rodríguez et al, 2017). Our data revealed that bacterial viability and strain pathogenicity are important factors in the induction of NETosis (Branzk et al, 2014). This differs from a guinea pig study, which showed that heat-killed Mtb-induced NETs in skin (Filio-Rodríguez et al, 2017). NETosis was also triggered by plasma from TB patients, suggesting a role for circulatory cytokines (Romano et al, 2022; An et al, 2019). For example, IL-8, a neutrophil chemoattractant known to be associated with TB (Ameixa and Friedland, 2002), induces NETosis by mobilizing calcium and activating the PKC pathway through interaction with G-coupled protein receptor (Gupta et al, 2005; Bréchard et al, 2005; Schorr et al, 1999).

Pharmacological inhibition of NETosis markers reduced the amount of NETosis induced by Mtb infection. NOX2/CYBB is a part of the NADPH complex, and inhibiting the gene with GSK2795039 did not completely prevent NET formation. This may imply involvement of other NADPH oxidase-independent mechanisms, such as MPO and NE (Fuchs et al, 2007; Papayannopoulos et al, 2010). In other lung infections, NE has been shown to impact disease outcome due to its tissue-destroying proteolytic activity (Fujie et al, 1999; Sahoo et al, 2014). Therapeutic agents currently undergoing investigation, like Sivelestat which targets NE, reduces ARDS associated pathology, and may require further investigation for its use in TB-associated pathology (Sahebnasagh et al, 2020).

Recent work has shown that the accumulation of NETs carrying NE and MPO is associated with acute alveolar injury in the lower respiratory tracts of severely ill COVID-19 patients (Ackermann et al, 2021; Ouwendijk et al, 2021; Wang et al, 2023).

NE-mediated proteolysis of ECM releases elastin degradative products (EDP) that are known to be potent mediators of inflammation and interstitial fibrosis (Starcher and Peterson, 1999). HDTs that target NE and MPO, may reduce TB pathological damage at multiple levels. This could be either by reducing NETosis through inhibition of neutrophil degranulation, or via the breakdown of elastin, a major component of ECM, and the preferential substrate of NE (Narasaraju et al, 2024). A recent report by Moreira-Texeira demonstrated that increased type I IFN signaling induces NETosis, promoting mycobacterial growth in the absence of GM-CSF. The type I IFN-induced NETosis was also associated with disease severity in TB-susceptible C3HeB/FeJ mice (Moreira-Teixeira et al, 2020a). This is consistent with a recent work by Chowdhury et al, which demonstrated that Mtb induces a type I IFN and PAD4-dependent NET release in mouse neutrophils, resulting in bacterial replication (Chowdhury et al, 2024). Interestingly, the NET release was associated with necrotic regions in granulomas obtained from non-human primates (Chowdhury et al, 2024), which is in agreement with our observation of NETosis localization with pathological damage in human TB granulomas.

Neutrophilic transcriptome signatures have been associated with active tuberculosis and were suggested as biomarkers for poor immune control of Mtb (Moreira-Teixeira et al, 2020b; Berry et al, 2010). We show that in clinical disease, NETosis-specific genes such as MPO, CYBB, NCF1 and NCF2 were upregulated in the blood of TB patients compared to healthy controls. Interestingly, the elevated expression of NETosis proteins were recapitulated in a human TST challenge model, confirming the important role of the neutrophil cell death in TB pathogenesis.

Future work could consider testing NETosis inhibitors as HDTs targeting lung pathological damage, and simultaneous inhibition of the multiple mediators of NETosis may result in enhanced reduction of Mtb-induced NET formation. However, currently, there are no in vitro models that recapitulate the complete cellular interaction that occurs within a granuloma environment. A limitation of our study is the lack of a broader non-TB disease comparator group. Including a disease control such as *Streptococcus pneumoniae*, where NETosis has been characterized (Domon and Terao, 2021), would have strengthened our understanding of neutrophil driven disease pathogenesis. For future studies, such broader disease controls should be considered for understanding of neutrophil-driven mechanisms in TB pathogenesis.

Overall, our data indicate that NETosis may contribute to tissue destruction in necrotizing caseum. In addition, we show that NETosis-specific proteins localize in regions of necrotic damage and that pharmacologically inhibiting these specific pathways reduces Mtb-induced NETs formation. Our study provides evidence suggesting NETosis as a potential mechanism driving TB pathology, which may be targeted for HDTs targeting TB-associated tissue destruction.

## Methods

**Table:** Reagents and tools table

Reagent/resource	Reference or source	Identifier or catalog number
**Clinical samples**		
Human lung samples	This study	Table EV1
Human blood samples	This study	Table 1
**Kits**		
Opal 6-Plex Manual Detection Kit	Akoya Biosciences	NEL811001KT
Bio-Plex Pro Human Cytokine 27-plex Assay	Bio-Rad	#M500KCAF0Y
PAXgene Blood RNA Kit	Qiagen	762174
NETosis imaging kit	Cayman Chemical	601750
iScript™ cDNA Synthesis Kit	Bio-Rad	1708890
**Antibodies**		
anti-Citrulline	Abcam	ab5103
anti-IP-10	Abcam	ab8098
anti-NE	Abcam	ab68672
anti-NOX2 (CYBB)	Abcam	ab80897
anti-NCF1	Abcam	ab111855
anti-MPO	Abcam	ab9535
**Oligonucleotides**		
PCR Primers	This study (ordered at IDT)	Table EV2
**Chemicals, Enzymes and other reagents**		
Sivelestat	MedchemExpress	Table 2
Verdiperstat	MedchemExpress	Table 2
GSK2795039	MedchemExpress	Table 2
**Software**		
NDP software (NDP view.2)	Hammamatsu	
ImageJ software	Fiji	
GraphPad Prism 9	GraphPad	
**Key equipment**		
Hammamatsu Zoomer RS2	Hammamatsu	Model C10730-12
Hamamatsu NanoZoomer S60	Hammamatsu	
Bio-Plex 200 plate reader	Bio-Rad	
CFX 96 Thermocycler	Bio-Rad	
Biotek®Cytation 5	Agilent	

### Study setting and sample collection

This study recruited participants from the KwaDabeka clinic and Prince Cyril Zulu Communicable Disease Center, in the eThekwini district of Durban in KwaZulu-Natal (KZN), South Africa. This study was approved by the Biomedical Research Ethics Committee (BREC) at the University of KwaZulu-Natal (BE022/13/BE0000365/2021). We confirm that all research was performed in accordance with relevant guidelines/regulations. Informed consent was obtained from all participants and/or their legal guardians, and experiments conformed to the principles set out by the VMA Declaration of Helsinki and the Department of Health and Human Services Belmont Report. We recruited TB patients who were newly diagnosed as GeneXpert positive (*n* = 30), and LTBI (*n* = 20) who were QuantiFERON (QFT) positive and healthy individuals (*n* = 20) (QFT negative). All participants were treatment naïve at the time of sample collection. Clinical characteristics of all 70 participants used in this study are listed in Table 1. Whole blood samples were collected and processed as previously described (Fisher et al, 2022b). Lung samples were obtained from individuals undergoing elective pneumonectomy at King Dinizulu Hospital Complex and were collected through an ongoing study at the Africa Health Research Institute (BE019/13).

Samples were obtained from thirty-nine participants (*n* = 39) presenting with a range of TB- associated lung pathological complications, at a collaborating hospital called King Dinizulu Hospital Complex, based in Durban, South Africa. Demographics and clinical characteristics of the study participants are listed in Table EV1. The study was carried out at Africa Health Research Institute, under an approved lung study program through the Biomedical Research Ethics Committee (BREC) at the University of KwaZulu-Natal (BE019/13). Each consenting participant was allocated a personal identifying data (PID). Lung tissue biopsies ranging from non-affected (no pathological damage) to mild and severely damaged, were carefully isolated and preserved in 4% formaldehyde solution.

### Proteomic analysis of human lung tissues

Proteomic analyses of human lung tissues were performed on our previously generated proteomics datasets that have been deposited into the PRIDE partner repository with the dataset identifier PXD003646, which can be accessed from the Proteome Xchange Consortium (http://proteomecentral.proteomexchange.org) (Marakalala et al, 2015). Histologically distinct compartments, including necrotic (caseum) and cellular (cell) regions, had been dissected from solid, caseous and cavitary granulomas with the Laser Pressure Catapulting (LPC) Palm instrument (Zeiss, Göttingen, Germany). The dissected tissues were lysed and subjected to in-gel digestion (Lys-C protease and trypsin) and peptide extraction. Liquid chromatography-tandem mass spectrometry (LC-MS-MS) analysis of resulting peptides was performed as single-shot runs, using a Q-Exactive mass spectrometer (Thermo Fisher Scientific) coupled online with a nanoflow ultra-high-pressure liquid chromatography (UHPLC) instrument (Easy nLC; Thermo Fisher Scientific). Mass spectra were analyzed using the MaxQuant computational platform version 1.3.0.5 and Andromeda against the UniProt FASTA human database, and proteome quantification was performed in MaxQuant using the XIC-based inbuilt label-free quantification (LFQ) algorithm as previously described(Marakalala et al, 2015). We analyzed neutrophil-associated proteins from these publicly available proteomics data. The heatmap plot was constructed from Z-scored normalized and log2-transformed LFQ protein intensities.

### Lung tissue histopathology

Lung samples were collected in 10% neutral buffered formalin and stored for a period of no less than 14 days before being processed as previously described (Wells et al, 2021). IHC was done following deparaffinization using a xylene free isopropanol to fix the tissue, the tissue was then embedded according to standard procedures before it was sectioned to 4 μm using a rotary microtome. Sections were then baked at 60 °C for 15 min, dewaxed twice with different xylenes and rehydrated using decreasing grades of alcohol and then washed with water. Pathology was determined with hematoxylin and eosin (HnE) staining using standard procedures. The tissue was dehydrated through increasing grades of alcohol. Slides were cleared with xylene and mounted with a mixture of distyrene, plasticizer and xylene (DPX).

### Immunohistochemistry staining on pathological lung tissue sections

Lung tissue containing samples of various granulomas were processed as previously described (Wells et al, 2021). A few lung samples that represented the observed pathology in this cohort (Table EV1) were selected for further use in this study. Briefly, lung tissue was mounted on slides after being cut 2–5-μM thick. The slides were baked at 56 °C for 15 min and then dewaxed in xylene before being rinsed in 100% ethanol. Slides were washed in water for 2 min and then heat induced antigen retrieval was performed for 30 min in Tris-sodium chloride (pH6.0). Slides were left to cool for 15 min and then washed under running water for 2 min.

Peroxide activity was blocked for 10 min using 3% hydrogen peroxide at room temperature. Phosphate-buffered saline-tween (PBS-T) was used to wash the slides before they were blocked with Novolink for 5 min. Primary antibodies anti-Citrulline H3(1:500) (ab5103), anti-IP-10 (1:500) (ab8098), anti-NE (1:500) (ab68672), anti-NOX2 (CYBB) (1:500) (ab80897), anti-NCF1 (1:500) (ab111855), and anti-MPO (1:100) (ab9535) antibodies (Abcam, CA, USA) were used to stain the tissue. Following incubation, the tissue was washed with PBS-T. Goat anti-rabbit horse radish peroxidase (HRP)-conjugated secondary antibodies (Abcam, CA, USA, 1:500) were diluted with blocking buffer, and added to the section and incubated at RT for 30 min. PBS-T was used to wash the section three times before the 3,3’ diaminobenzidine (DAB) staining for 5 min under running water and counterstained for 2 min with hematoxylin. Following staining, the sections were washed with distilled water, allowed to blue in 3% ammonium water for 30 s and washed with distilled water, dehydrated in a series of graded ethanol and mounted on slides using DPX. Slides were left to fix overnight before the images were acquired on the Hammamatsu NDP viewer slide (Hammamatsu Zoomer RS2, Model C10730-12) and viewed using NDP software (NDP view.2). Images were analysed using ImageJ software.

### Immunofluorescence (IF) staining

Tissue sections were first deparaffinized and underwent antigen retrieval using the PT Link system with EnVision™ Flex target retrieval solution high pH (Agilent, Denmark). The samples were then washed in 1x EnVision™ Flex wash buffer (DM831, ref.: K8000/K8002, Agilent, Denmark) for 10 min (2 × 5-min washes). A hydrophobic pen was used to encircle the tissue of interest to facilitate staining. The sections were blocked for endogenous peroxidase activity with blocking buffer (S202386-2, Agilent, Denmark) for 10 min (2 × 5-min washes) at room temperature (RT), followed by another 10-min wash in the wash buffer.

A second blocking step was performed using 0.05 g BSA, 0.5 ml goat serum in 4.5 ml wash buffer for 20 min, followed by a wash. The primary antibody was then added and incubated for 40 min at RT, after which the slides were washed (2 × 5-min washes). The antibody was conjugated to the secondary Opal Polymer antibody (HRP: Ms + Rb) and incubated for 20 min at RT. A fluorescent dye with a wavelength of 494/525 nm (FITC: OPAL 520 reagent, AKOYA Biosciences, USA) was applied at a 1:200 dilution in 1x Plus Amplification buffer and incubated for 10 min.

For the second antibody, tissue antigens were exposed by boiling the slides in a TSC solution (10% Antigen Retrieval 6 buffer, K800421-2, Agilent, Denmark, diluted in distilled water). This was done by heating the slides in the microwave under three conditions: 2 min on high, 5 min on medium, and 10 min on low. The slides were then cooled by placing the TSC solution under running tap water for 20 min. After cooling, the tissue was equilibrated by rinsing the slides in 1x EnVision™ Flex wash buffer for 5 min at RT. The second primary antibody was added following the same steps as the first, with a secondary Opal Polymer antibody and a second fluorochrome with a wavelength of 550/570 nm (TRITC: OPAL 570 reagent, AKOYA Biosciences, USA).

The process was repeated for the third primary antibody, which was labeled with a fluorochrome of wavelength 676/694 nm (Cy5: OPAL 620 reagent, AKOYA Biosciences, USA). To complete the staining, the tissue samples were stained with DAPI for 5 min at RT. Afterward, the slides were washed and mounted using Dako fluorescent mounting media and covered with coverslips. Finally, the slides were scanned and analyzed using the Hamamatsu NanoZoomer S60 (Japan).

### Measurement of plasma cytokines by multiplex ELISA

A multiplex Luminex assay was performed using the Bio-Plex 27 human cytokine screening panel, a 27-plex kit from Bio-Rad (Hercules, CA, USA) to measure interleukin (IL)-1β, IL-1 receptor antagonist (IL-1RA), IL-2, IL-4, IL-5, IL-6, IL-7, IL-8, IL-9, IL-10, IL-12, IL-13, IL-15, IL-17, Eotaxin, fibroblast growth factor (FGF) basic, granulocyte colony stimulating factor (G-CSF), granulocyte macrophage colony stimulating factor (GM-CSF), interferon-γ (IFN- γ), interferon gamma inducible protein (IP-10), monocyte chemoattractant protein-1 (MCP-1), macrophage inflammatory protein alpha (MIP-1α), platelet derived growth factor (PDGF-bb), MIP-1β, regulated on activation, normal T cell expressed and secreted (RANTES), tumor necrosis factor (TNF-α) and vascular endothelial growth factor (VEGF). Assays were performed as per the manufacturer’s instructions, and qualitative values were obtained with the Bio-Plex 200 plate reader (Hercules, CA, USA). The sensitivity of the kit was 0.2–45.6 pg/ml for each of the 27-cytokines measured. Samples and standards were run in duplicate. The Bio-Plex-manager software version 6 was used to collect the data, and a 5PL regression formula was used to generate the standard curves for each cytokine to interpolate the concentration of cytokines in the samples. The midpoint between zero and the lowest measured expression for a specific cytokine was used to quantify cytokines that were expressed lower than the lower limit of detection. The samples were diluted according to the manufacturer’s instructions for plasma samples.

### RNA isolation and cDNA synthesis

RNA was isolated and cDNA synthesis performed as previously described (Fisher et al, 2022b). Briefly, 2.5 ml of whole blood was collected in PAXgene™ tubes, and RNA was isolated using the Paxgene™ kit (PreAnalytix, Hombrechtikon, Switzerland). Complementary DNA (cDNA) was synthesized from isolated RNA as per the manufacturer’s instructions (Bio-Rad, Hercules, CA). Briefly, RNA was adjusted to a concentration of 500 ng for cDNA synthesis, and an appropriate volume of the reaction mix containing 5–20 µl of nuclease-free water, 1 µl of RNA, and 4 µl of iScript 5× was added to each sample for a total reaction volume of 20 µl. The T100 thermocycler from Bio-Rad (Hercules, CA, USA) was set to 5 min at 25 °C, 30 min at 42 °C, 5 min at 85 °C.

### Real-time quantitative polymerase chain reaction for gene expression analysis

Real-time quantitative polymerase chain reaction (RT-qPCR) was done to determine the expression of neutrophil-specific genes as per the manufacturer’s instructions pertaining to the iTaq™ Universal SYBR green supermix (Bio-Rad, Hercules, CA, USA). The total reaction volume was 10 µl. Briefly, 1 µl of cDNA was added to each well with 9 µl of mastermix consisting of the relevant forward and reverse primer at 0,5 µl each, 5 µl of iTaq™ Universal SYBR green supermix (Bio-Rad, Hercules, CA, USA) and 3 µl of nuclease-free water. The CFX 96 thermocycler (Bio-Rad, Hercules, CA) was set to the following protocol: 30 s at 95 °C, 5 s at 95 °C, 30 s at 56 °C for 39 cycles. The melt curve analysis was done at 65–95 °C with 0.5 °C increments. Glyceraldehyde 3-phosphate dehydrogenase (GAPDH) was used as the housekeeping gene, and all qPCR data were normalized to GAPDH expression (Barber et al, 2005).

### Primer design

Primers were designed using the IDT primer design tool, PrimerQuest Tool and sequences were blasted using the BlastN tool on NCBI. Primers were designed for MPO, Azu1, CYBB, CYBA, NCF1, NCF2, NCF4, LCN2, NE, DEFA1, S100A8, S100A9, S100A12, MMP8, DEFA1, DEFA4, MMP9, and CD117 (see Table EV2).

### Transcriptional profiling of neutrophil-related genes in the human TST challenge model

Transcriptional profiling of neutrophil-related genes was analyzed using our previously published dataset (Pollara et al, 2021), obtained from a human TST challenge model, in which individuals with LTBI or TB were exposed to Mtb-specific antigen, via a tuberculin skin test (TST), and the immune response was analyzed at the site of TST challenge (Pollara et al, 2021). Assessment of the TST transcriptome was performed by RNA-Seq as previously reported, and comparison was made between TST transcripts from LTBI and TB samples (Pollara et al, 2021; Bell et al, 2016). The cDNA libraries had been generated using the KAPA Hyperprep kit (Roche), and sequencing had been performed on the Illumina Nextseq using the Nextseq 500 High Output 75 cycle kit (Illumina) according to the manufacturer’s instructions. The R/Bioconductor package tximport was used to import the mapped counts data and summarize the transcript-level data into gene-level data.

### NETosis kinetics and live imaging

A NETosis imaging kit (Cayman Chemical, Ann Arbor, Michigan, USA) was used to quantify the amount of NETosis induced by Mtb-infected healthy neutrophils. The assay was performed as per the manufacturer’s instructions and as previously described (Fisher et al, 2022b) with the following modifications. When using gene inhibitors, the neutrophils were incubated with various concentrations of inhibitors for 30 min before any stimulation or infections occurred. About 100 μl of PMA, a most frequently used stimulus with a 100% success rate for inducing NETosis (Hoppenbrouwers et al, 2017), was used as a positive control at a concentration of 200 nM and 10% Triton X-100, which causes cell death release through non-netotic means such as necrosis (Gupta et al, 2017), was used as an absolute negative control. In addition, neutrophils that were untreated were also used as a negative control. To determine various inducers of NETosis, heat-killed (HI) Mtb H37Rv, *Mycobacterium smegmatis* (M.smeg) and live Mtb were added to the appropriate wells at an MOI of 10:1, and 100 μl of TB plasma was added to the appropriate wells, while unstimulated neutrophils were used as an uninduced control. About 50,000 cells/well were seeded into the wells in a volume of 100 µl in a flat-bottom 96-well plate. About 100 µl of stimuli or NETosis Imaging Buffer (1x) was added to each well for a final volume of 200 µl. The plate was centrifuged at 250×*g* to pellet the cells at the bottom of the plate. The Biotek®Cytation 5 (Biotek, Winooski, Vermont, USA), which uses brightfield and fluorescence microscopy, was used to image activity every 30 min for 6–12 h at a temperature of 37 °C and 5% CO_2_. Green fluorescence indicates extracellular DNA and thus NETosis activity, and red fluorescence is a marker for an intact cell. The Biotek® cytation 5 counts the number of cells that are fluorescing green and the number of cells that are red. The Gen5 software then creates a Microsoft Excel spreadsheet with these numbers according to time and plate layout for further analysis.

### *Mycobacterium tuberculosis* culturing

7H9 broth media was made by dissolving 4.79 mg of 7H9 Middlebrook powder (BD, Franklin Lakes, NJ, USA) in a total of 1000 ml deionized water containing 0.2% glycerol, 10% OADC (BD, Franklin Lakes, NJ, USA), and 0.05% Tween 80 (BD, Franklin Lakes, NJ, USA). The media was filter sterilized using a 1 L Corning filtering system with a 2-um membrane. The media was kept at 4 °C. Mtb H37Rv was cultured in media, and the OD was read to determine the quantity of bacilli in the media. Starter cultures were prepared by adding 1 ml of Mtb H37Rv to 5 ml of media and sub-cultured on day 5. 1 ml was removed from the starter culture and added to 9 ml of media, and incubated at 37 °C in a shaking incubator at 150 rpm. M.smeg was cultured in 7H9 media overnight at 37 °C in a shaking incubator at 150 rpm. M.smeg was heat-inactivated at 121 °C for 45 min before neutrophils were infected. We used an MOI of 10 and 1 based on previous literature (Braian et al, 2013; Dallenga et al, 2017). Mtb was heat-inactivated at 121 °C for 10 min.

### Pharmaceutical inhibitors

The gene inhibitors used are listed in Table 2 and were purchased from MedChemExpress (Monmouth Junction, NJ, USA).

**Table 2:** Drugs used to target NETosis-specific pathways.

Drug name	Target	Clinical trial	Trial number	
Sivelestat	Neutrophil elastase	Phase II	NCT00036062	(Tagami et al, 2014)
Verdiperstat	myeloperoxidase	Phase II	NCT02388295	(Ren et al, 2022)
GSK2795039	CYBB/NOX2	n/a	n/a	(Hirano et al, 2015)

### Statistical analysis

Data were analysed using Microsoft Excel and GraphPad Prism 9. Images were analyzed by NDP.view2 and ImageJ. Healthy, LTBI, and TB groups were compared using an unpaired *t*-test to determine whether differences were significant. Significant difference was defined as *p* < 0.05.

### Graphics

The figure for the synopsis used in this manuscript was created using BioRender.com

### Ethics statement

This study was approved by the Biomedical Research and Ethics Committee, at the University of KwaZulu-Natal. The blood (BE022/13/BE0000365/2021) and lung tissue (BE019/13) samples were obtained through an ongoing study at the Africa Health Research Institute.

## Supplementary information

Table EV1

Table EV2

Peer Review File

Source data Fig. 1

Source data Fig. 2

Source data Fig. 3

Source data Fig. 4

Source data Fig. 5

Source data Fig. 6

Source data Fig. 7

Expanded View Figures

## Data Availability

This study generated no data that requires deposition in a public database. The source data of this paper are collected in the following database record: biostudies:S-SCDT-10_1038-S44321-026-00435-3.
