# Supplementary material for: NETosis associates with human TB lung tissue destruction and disease pathogenesis
Source: EMBO Mol Med. 2026 Jun 2;18(7):2547–72. doi: 10.1038/s44321-026-00435-3 (PMC13365388; doi:10.1038/s44321-026-00435-3)
Supplement: Supplementary file 2 — Table EV2 [file 44321_2026_435_MOESM2_ESM.docx]

| **Table EV2: Primer sequences designed for gene expression of neutrophil specific genes** | | |
| --- | --- | --- |
| **Target gene** | **Forward /Reverse Primer sequence** | **NM accession number** |
| GAPDH | F: 5’-AAG GTC GGA GTC AAC GGA TT-3’ | 001357943.2 |
|  | R: 5’-CTC CTG GAA GAT GGT GAT GG-3’ |  |
| MPO | F: 5’-CCA TGG TCC AGA TCA TCA CTT AC-3’ | 000250.2 |
|  | R: 5’-CAT CAG TTT CCT CGC CAA TTT C-3’ |  |
| NE | F: 5’-ATC GTG ATT CTC CAG CTC AAC-3’ | 001972.4 |
|  | R: 5’-GGA GGC AAT TCC GTG GAT TA-3’ |  |
| CYBA | F: 5’-TTG GTG CCT ACT CCA TTG TG-3’ | 000101.4 |
|  | R: 5’-TTG GTG CCT ACT CCA TTG TG-3’ |  |
| CYBB | F: 5’-ACC CTC CTA TGA CTT GGA AAT G-3’ | 000397.4 |
|  | R: 5’-GCC AGT GAG GTA GAT GTT GTA G-3’ |  |
| NCF1 | F: 5’-ACA CCT TCA TCC GTC ACA TC-3’ | 000265.6 |
|  | R: 5’-GTA TGG CTC ACC TGC ATA GTT-3’ |  |
| NCF2 | F: 5’-CAG AGA AGG CCT TTA CCA GAA G-3’ | 001190789.1 |
|  | R: 5’-TCT CAG GCA CAA ACC CAA ATA-3’ |  |
| NCF4 | F: 5’-CGA GAG CAG AGG CTC TAT TTG-3’ | 000631.5 |
|  | R: 5’-GCT GAT GGT GTC TTC GTA GTA G-3’ |  |
| S100A8 | F: 5’-GAA GAC CTG AAG GTT CTG TTT-3’ | 001319196.1 |
|  | R: 5’-GCC ACG CCC ATC TTT ATC-3’ |  |
| S100A9 | F: 5’-GCT CCT CGG CTT TGA CAG-3’ | 002965.4 |
|  | R: 5’-CAG CTG CTT GTC TGC ATT TG-3’ |  |
| S100A12 | F: 5’-TGC TGT AGC TCC ACA TTC C-3’ | 005621.2 |
|  | R: 5’-CTT TGA TAT TCT TGA TGG TGT TTGC-3’ |  |
| DEFA1 | F: 5’-GCC TAG CTA GAG GAT CTG TGA-3’ | 004084.3 |
|  | R: 5’-CTG GTA GAT GCA GGT TCC ATA G-3’ |  |
| DEFA4 | F: 5’-TGG CTG CTC TTG CTA CAT AAG-3’ | 001925.3 |
|  | R: 5’-GTT GAG CCT GAA ACC TGA AGA-3’ |  |
| MMP8 | F: 5’-ACC AAC ACC TCC GCA AAT TA-3’ | 002424.3 |
|  | R: 5’-TTC ACG GAG GAC AGG TAG AA-3’ |  |
| LCN2 | F: 5’-ACT TCC AGG ACA ACC AAT TCC-3’ | 005564.5 |
|  | R: 5’-GAG ATT TGG AGA AGC GGA TGA A-3’ |  |
| CD177 | F: 5’-CTT GTC TAT GGA AGG CTG TCT G-3’ | XM017027022.2 |
|  | R: 5’-TCC TCT CCT CTG TTC TGT AGT G-3’ |  |
| AZU1 | F: 5’-CTG CTT CCA AAG CCA GAA C-3’ | 001700.5 |
|  | R: 5’-GAG GAG AGA TCG GCT TCT TTA T-3’ |  |
