# Supplementary material for: NETosis associates with human TB lung tissue destruction and disease pathogenesis
Source: EMBO Mol Med. 2026 Jun 2;18(7):2547–72. doi: 10.1038/s44321-026-00435-3 (PMC13365388; doi:10.1038/s44321-026-00435-3)
Supplement: Supplementary file 4 — Source data Fig. 1 [file 44321_2026_435_MOESM4_ESM.zip › Figure 1/Figure 1A/Source of data for Heatmap in Figure 1A.docx]

The Heatmap in **Figure 1A** (as below) was generated using previously generated

proteomics datasets that have been deposited into the PRIDE partner repository with the dataset identifier *PXD003646,* which can be accessed from the Proteome Xchange Consortium (<http://proteomecentral.proteomexchange.org>)


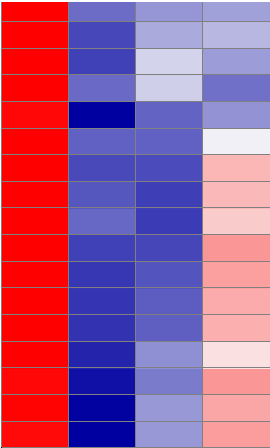

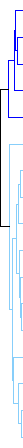

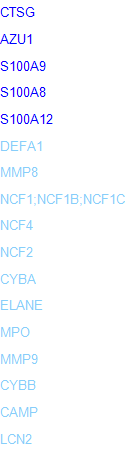

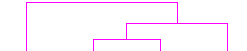


Cavitary

Granuloma,

Caseum

Solid

Granuloma,

Cellular

Cavitary

Granuloma,

Cellular

Caseous

Granuloma,

Caseum


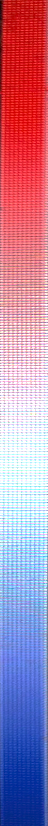


0

0.5

-0.5

-1

1
