# Supplementary figures and images for: NETosis associates with human TB lung tissue destruction and disease pathogenesis
Source: EMBO Mol Med. 2026 Jun 2;18(7):2547–72. doi: 10.1038/s44321-026-00435-3 (PMC13365388; doi:10.1038/s44321-026-00435-3)

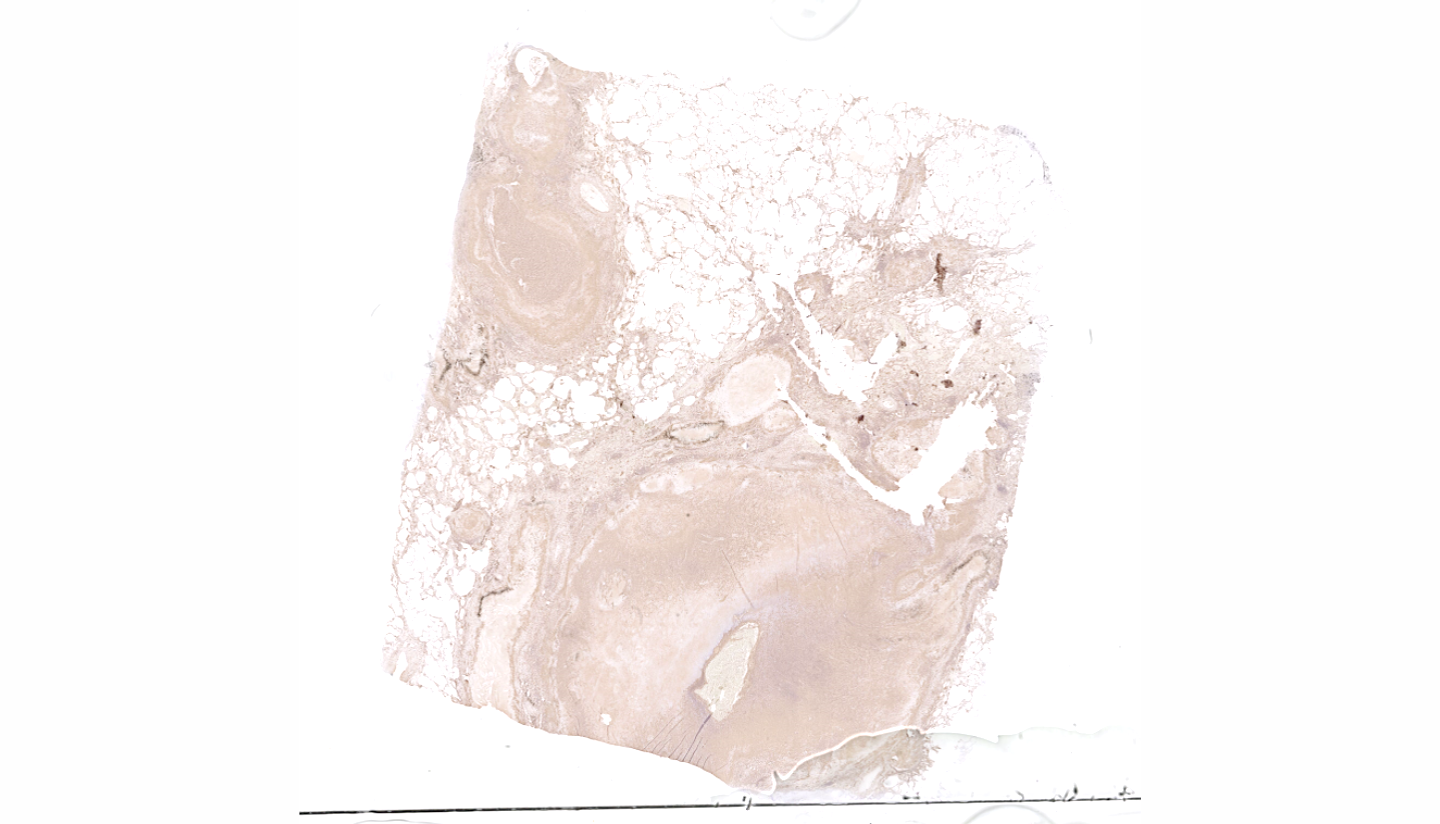

Supplement: Supplementary file 4 — Source data Fig. 1 [file 44321_2026_435_MOESM4_ESM.zip › Figure 1/Figure 1F/20314 NOX 2 III.tif]

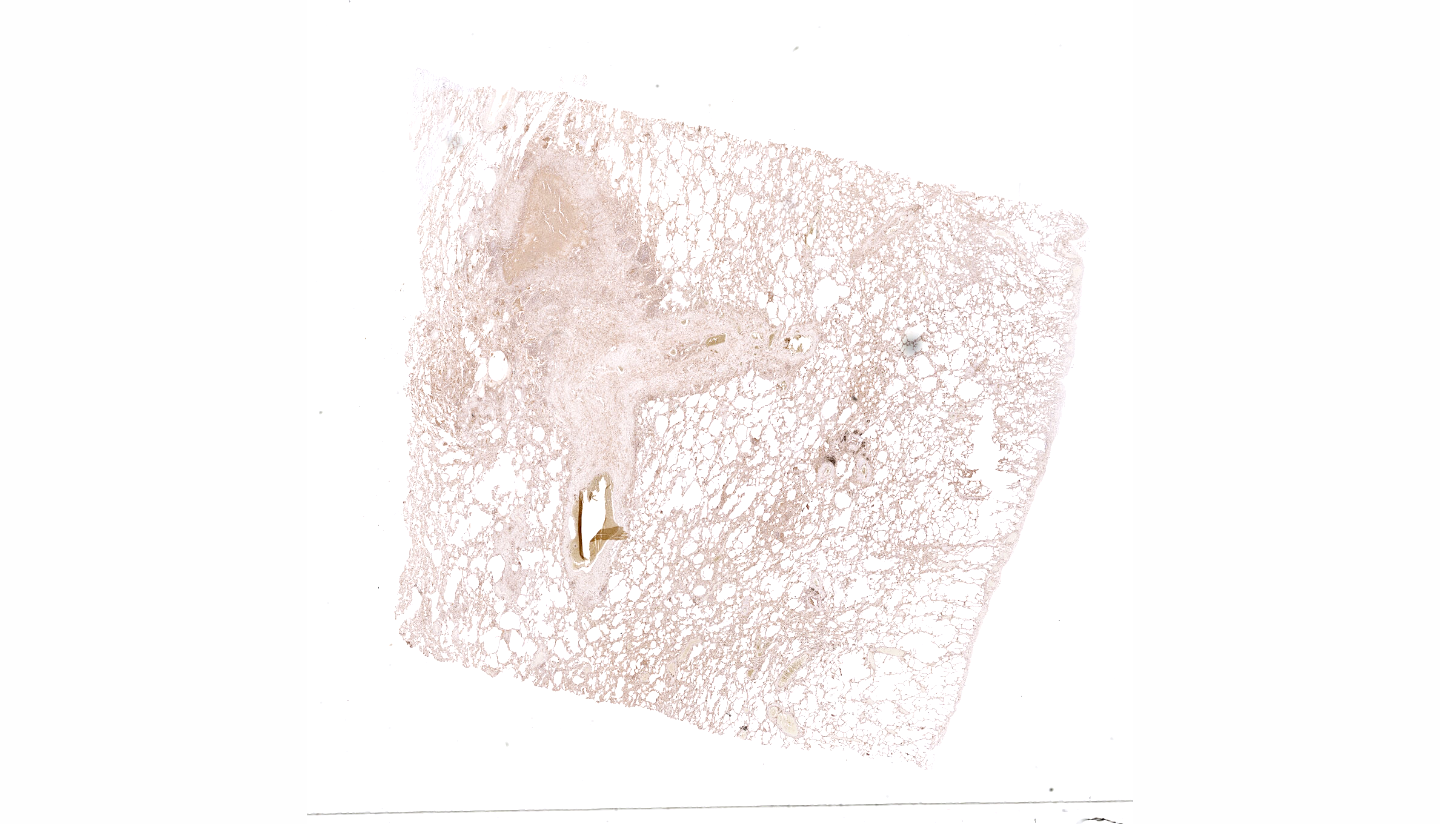

Supplement: Supplementary file 4 — Source data Fig. 1 [file 44321_2026_435_MOESM4_ESM.zip › Figure 1/Figure 1F/13462 NOX 2 I and III.tif]

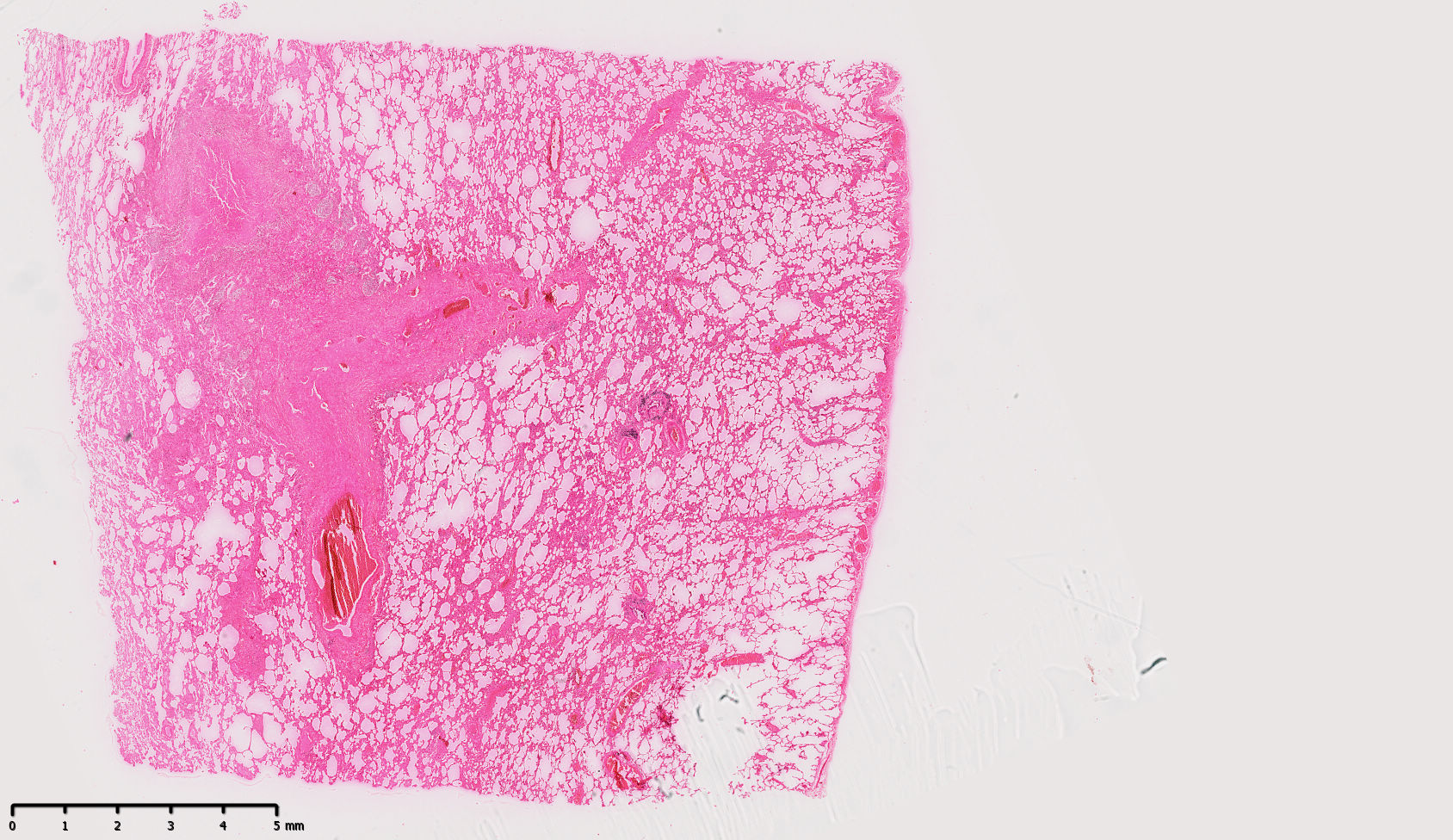

Supplement: Supplementary file 4 — Source data Fig. 1 [file 44321_2026_435_MOESM4_ESM.zip › Figure 1/Figure 1B/H&E x0.54jpg.jpg]

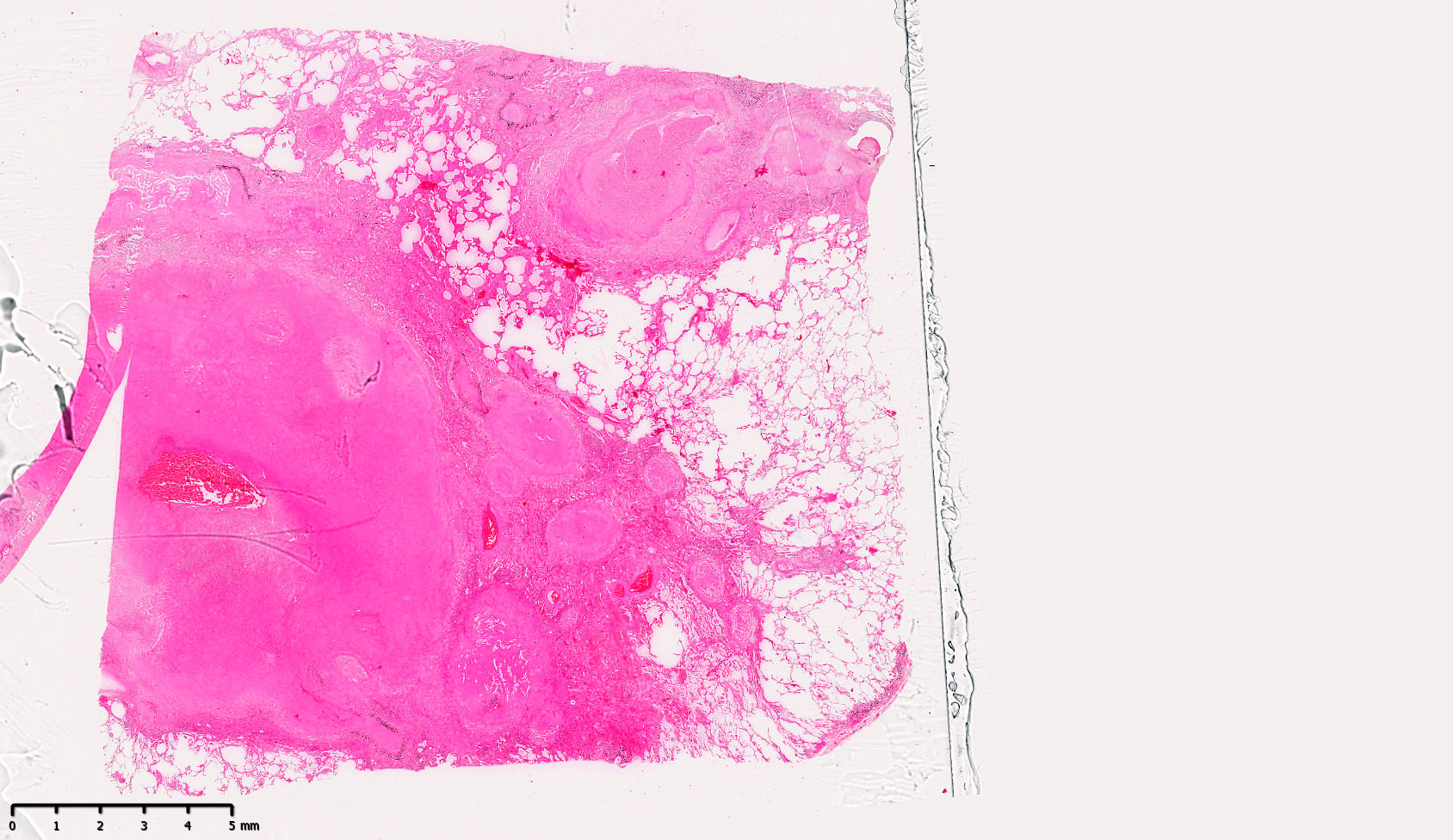

Supplement: Supplementary file 4 — Source data Fig. 1 [file 44321_2026_435_MOESM4_ESM.zip › Figure 1/Figure 1B/H&E x0.45jpg.jpg]

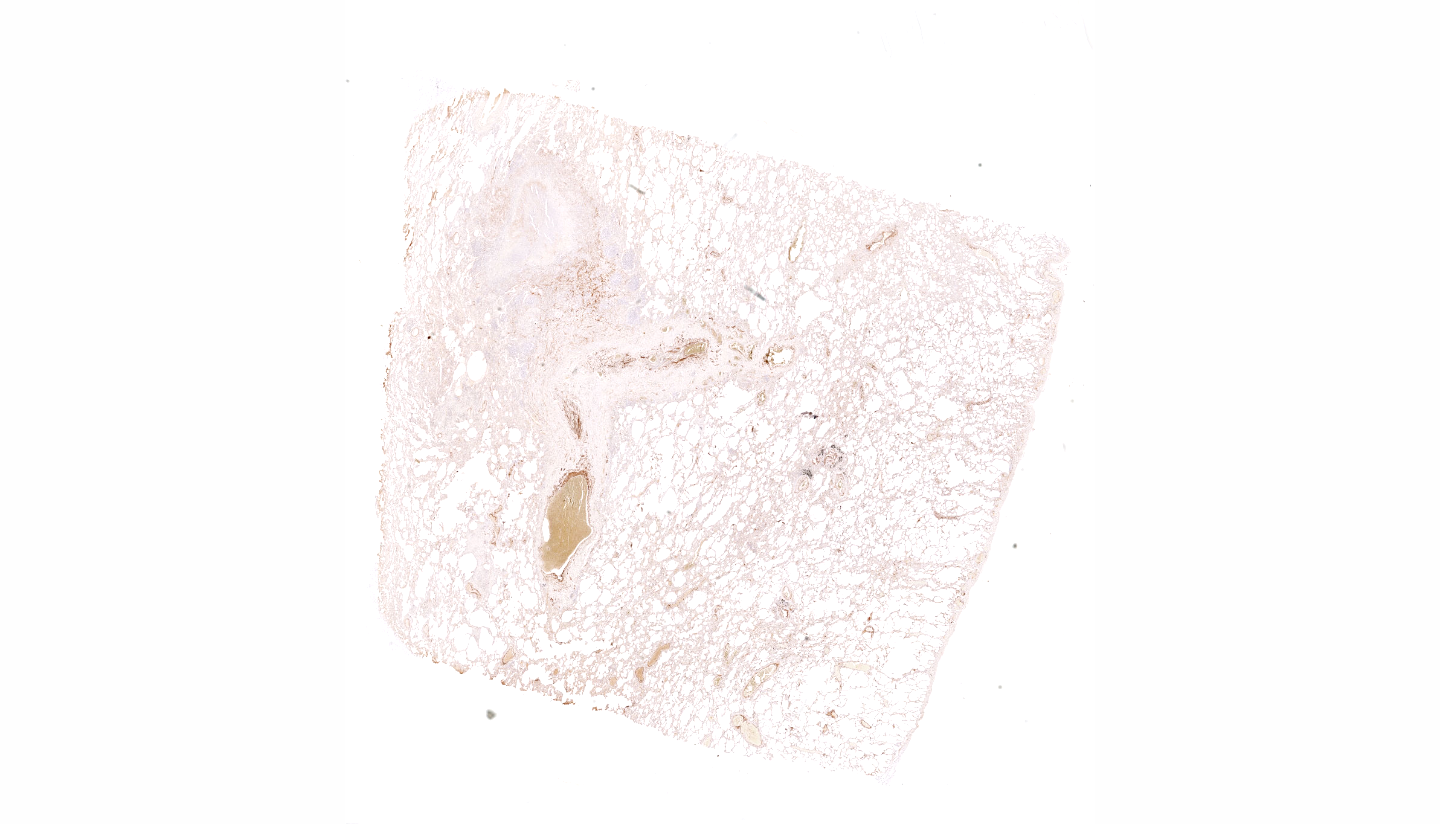

Supplement: Supplementary file 4 — Source data Fig. 1 [file 44321_2026_435_MOESM4_ESM.zip › Figure 1/Figure 1E/13462 NCF-1 -I and III.tif]

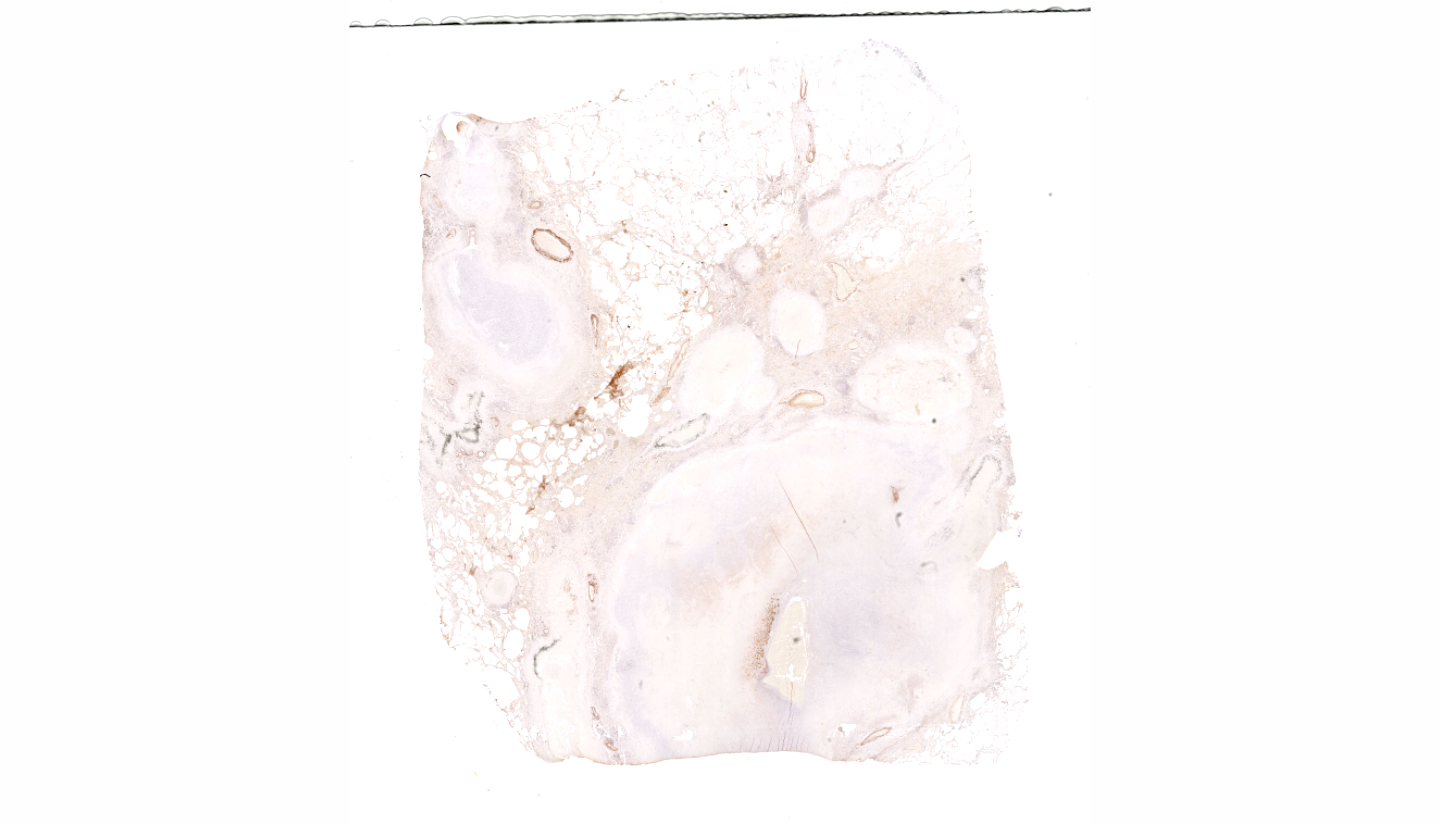

Supplement: Supplementary file 4 — Source data Fig. 1 [file 44321_2026_435_MOESM4_ESM.zip › Figure 1/Figure 1E/20314 NCF-1 - III.tif]

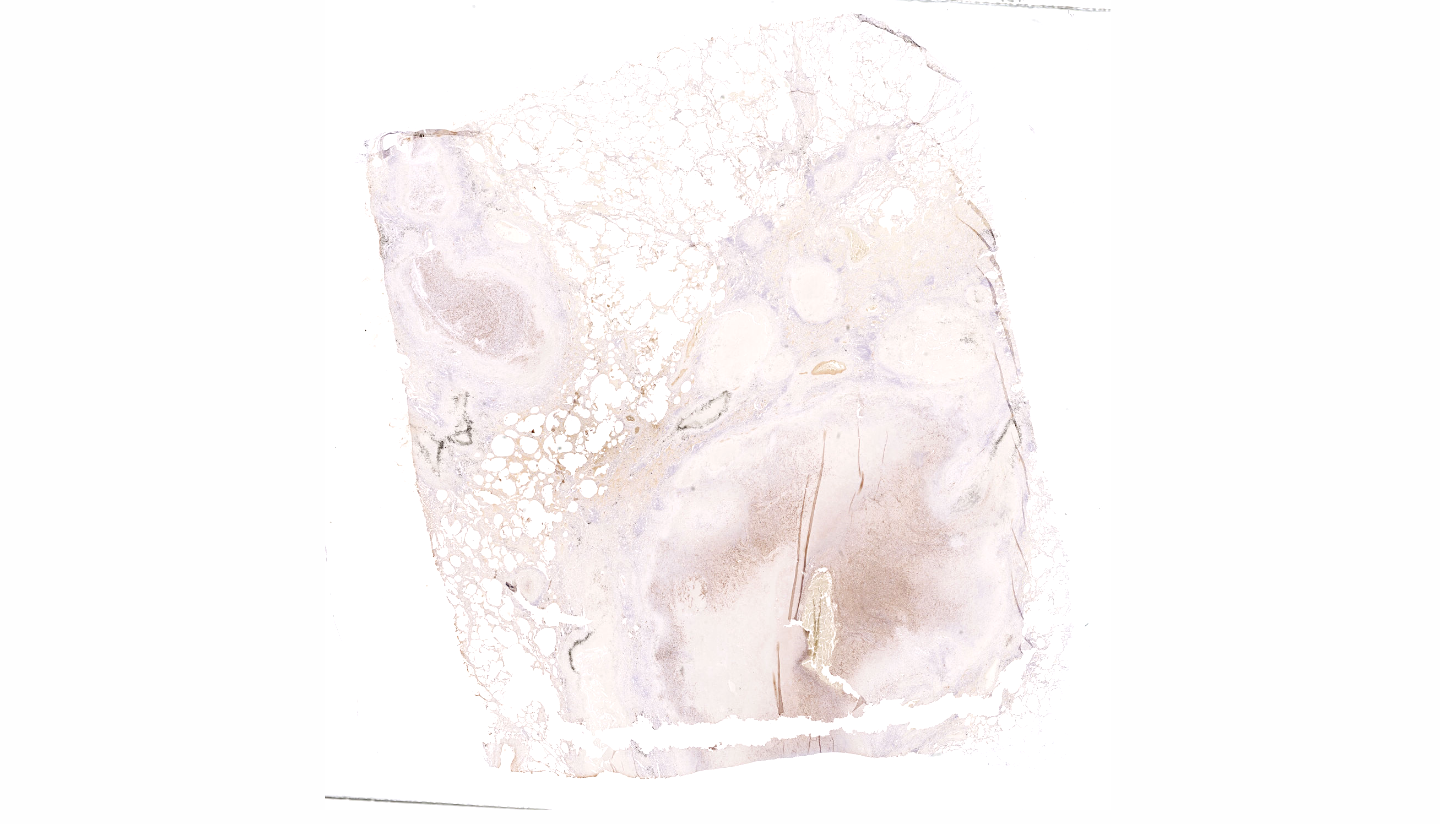

Supplement: Supplementary file 4 — Source data Fig. 1 [file 44321_2026_435_MOESM4_ESM.zip › Figure 1/Figure 1D/20314 09 E NE II.tif]

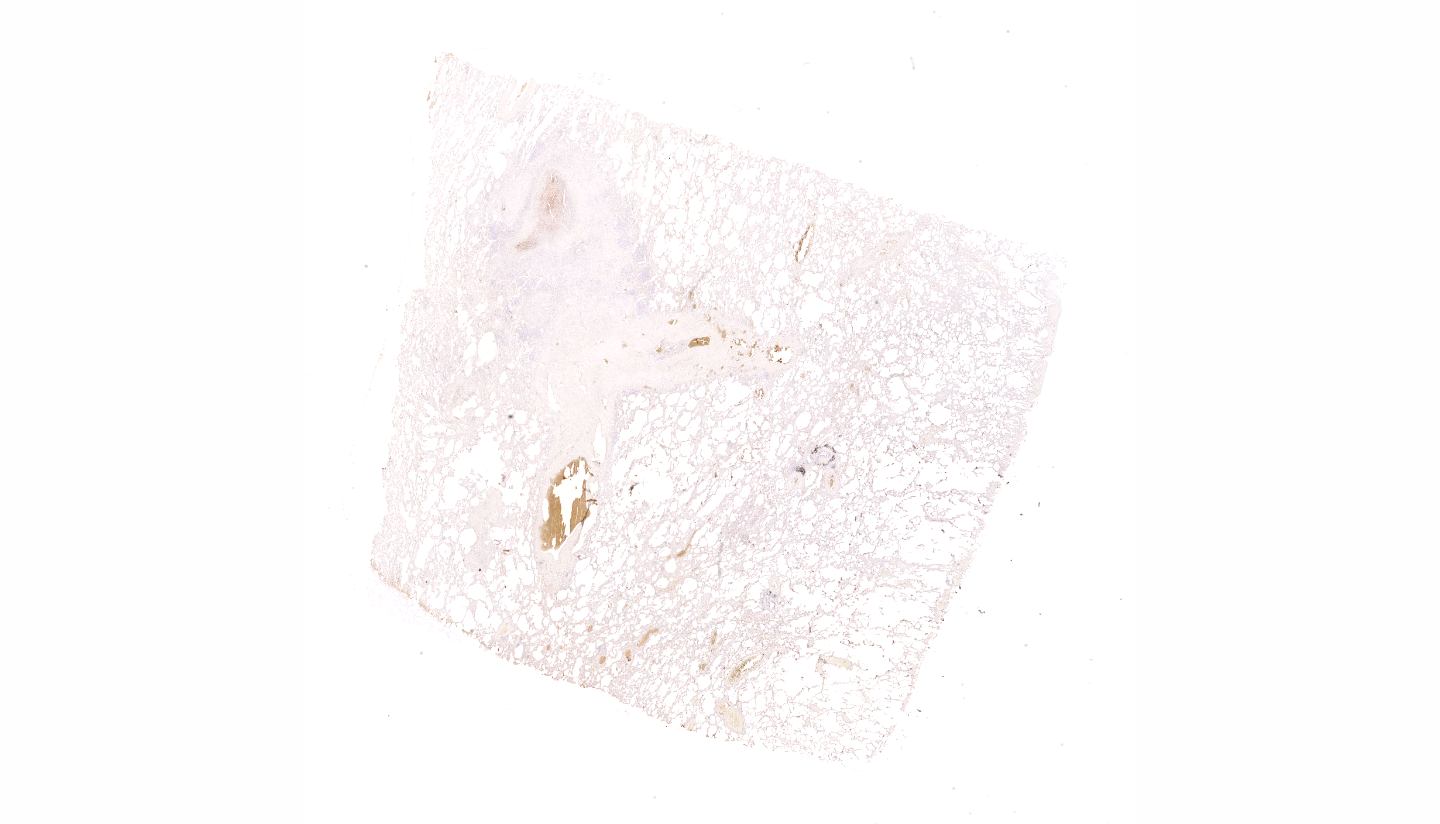

Supplement: Supplementary file 4 — Source data Fig. 1 [file 44321_2026_435_MOESM4_ESM.zip › Figure 1/Figure 1D/13462 09 NE I and III.tif]

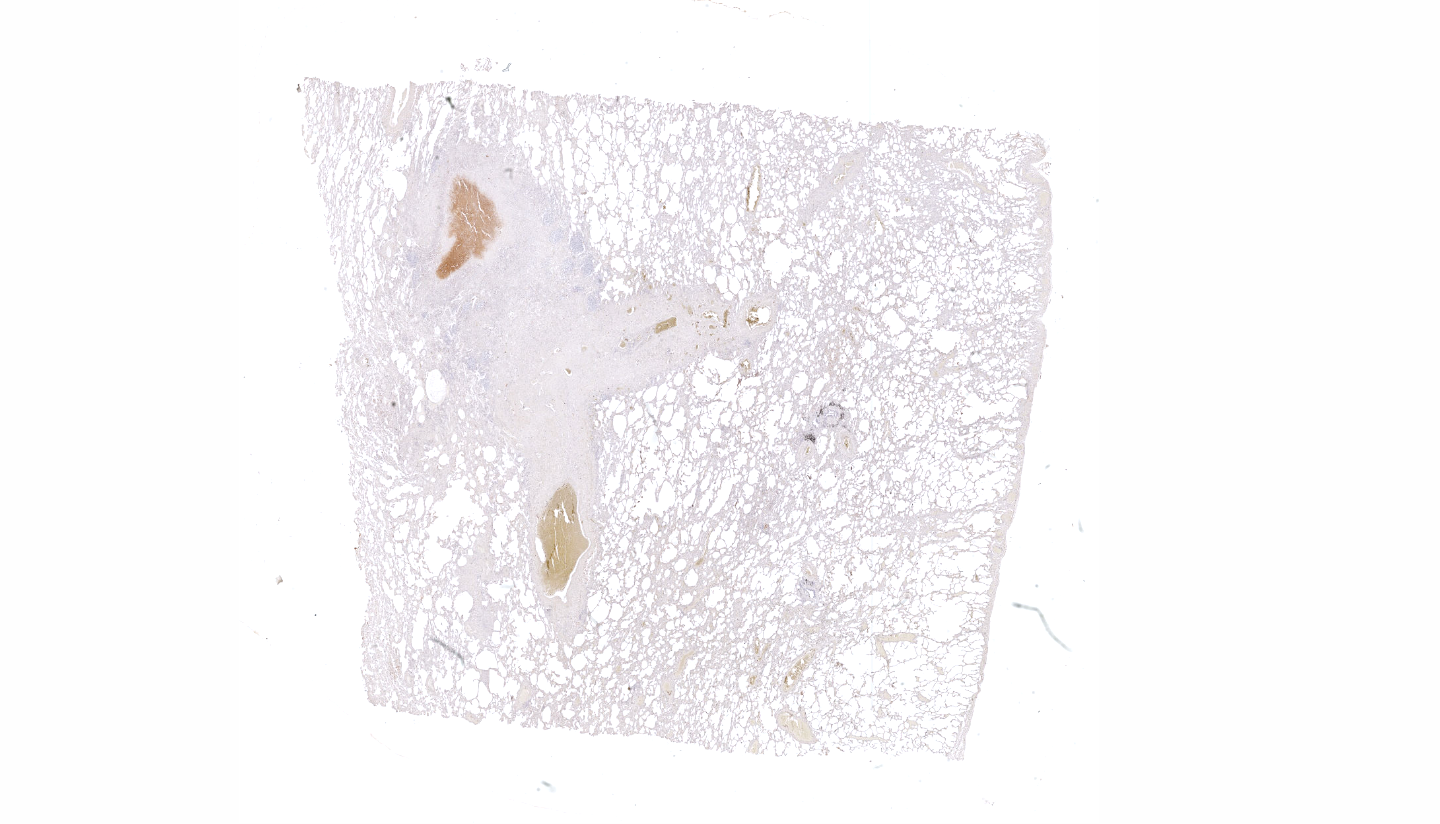

Supplement: Supplementary file 4 — Source data Fig. 1 [file 44321_2026_435_MOESM4_ESM.zip › Figure 1/Figure 1C/MPO - 13462 I and III.tif]

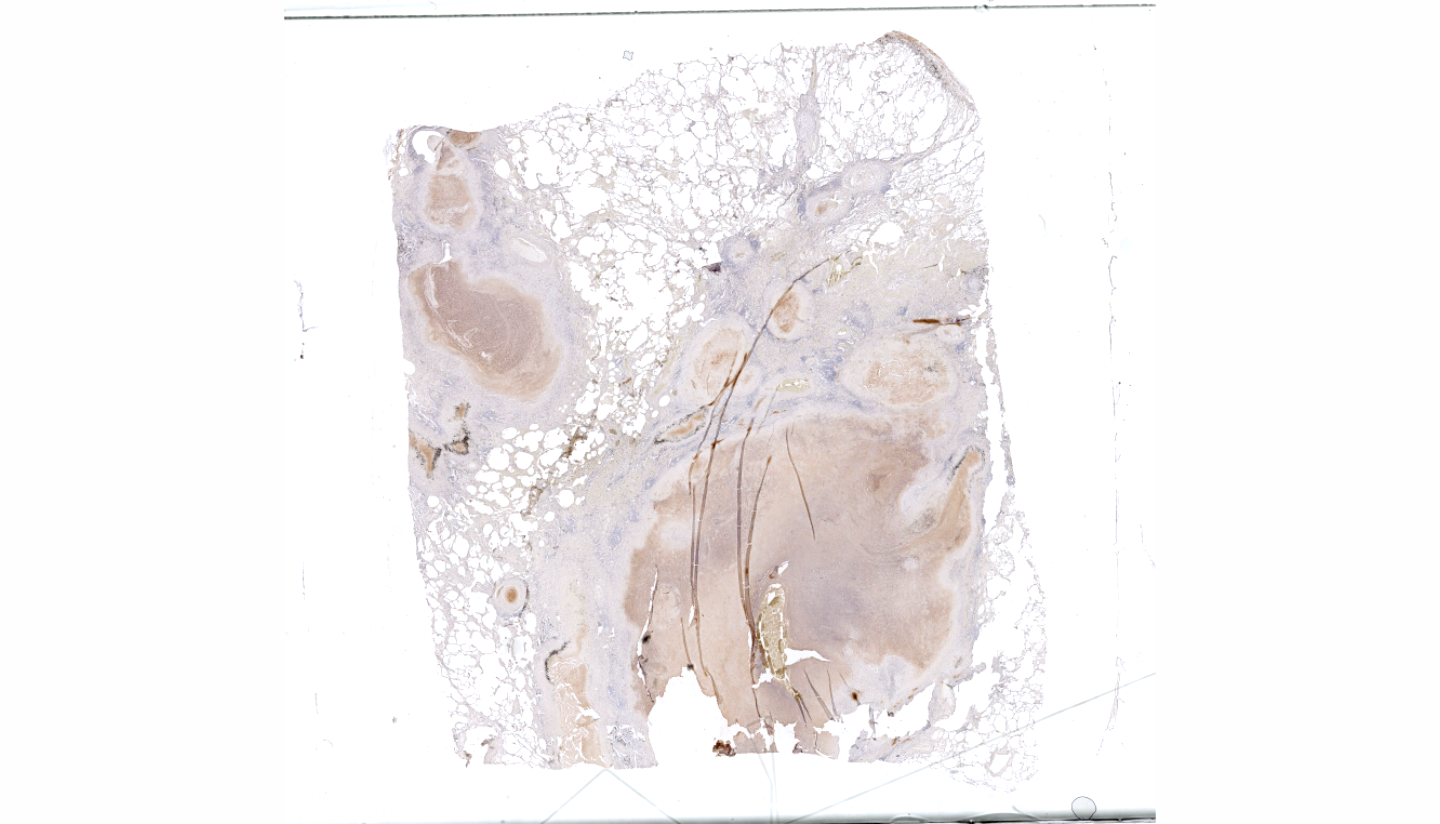

Supplement: Supplementary file 4 — Source data Fig. 1 [file 44321_2026_435_MOESM4_ESM.zip › Figure 1/Figure 1C/MPO - 20314 II.tif]

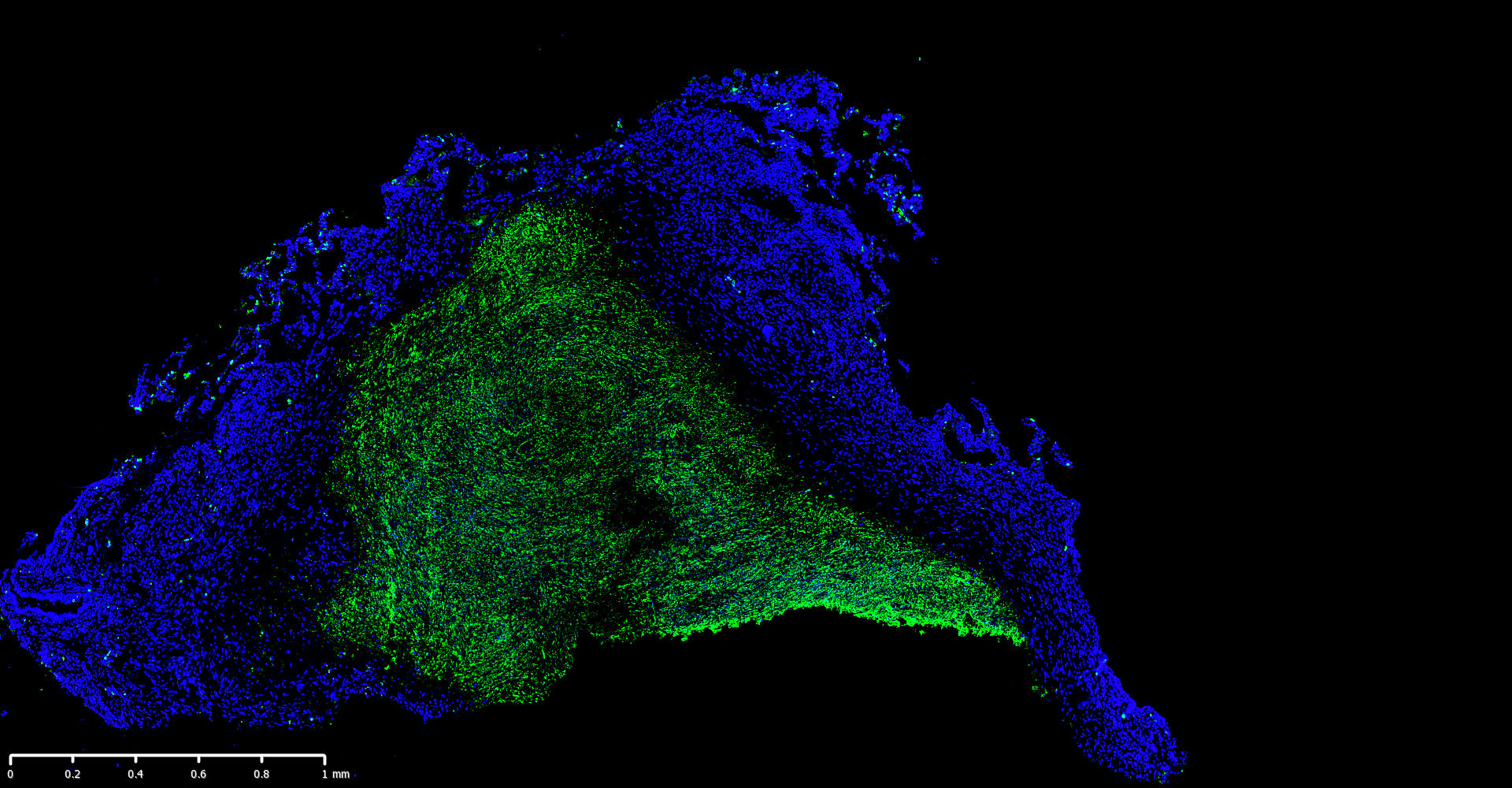

Supplement: Supplementary file 5 — Source data Fig. 2 [file 44321_2026_435_MOESM5_ESM.zip › Figure 2/Figure 2C/MPO green _ DAPI blue x3.53.jpg]

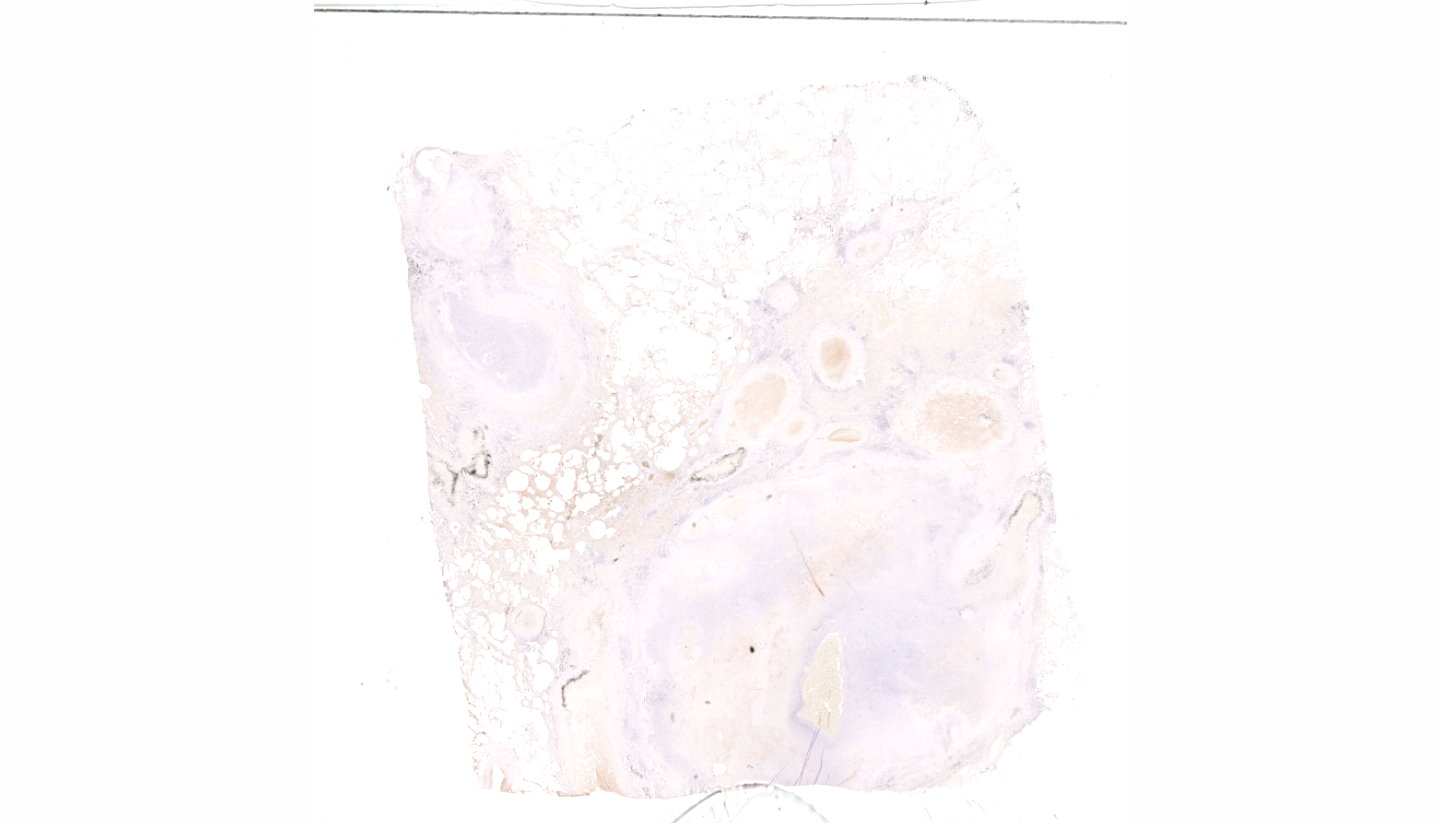

Supplement: Supplementary file 5 — Source data Fig. 2 [file 44321_2026_435_MOESM5_ESM.zip › Figure 2/Figure 2D/20314 09 E IP-10 - 2022-03-10 15.52.55.tif]

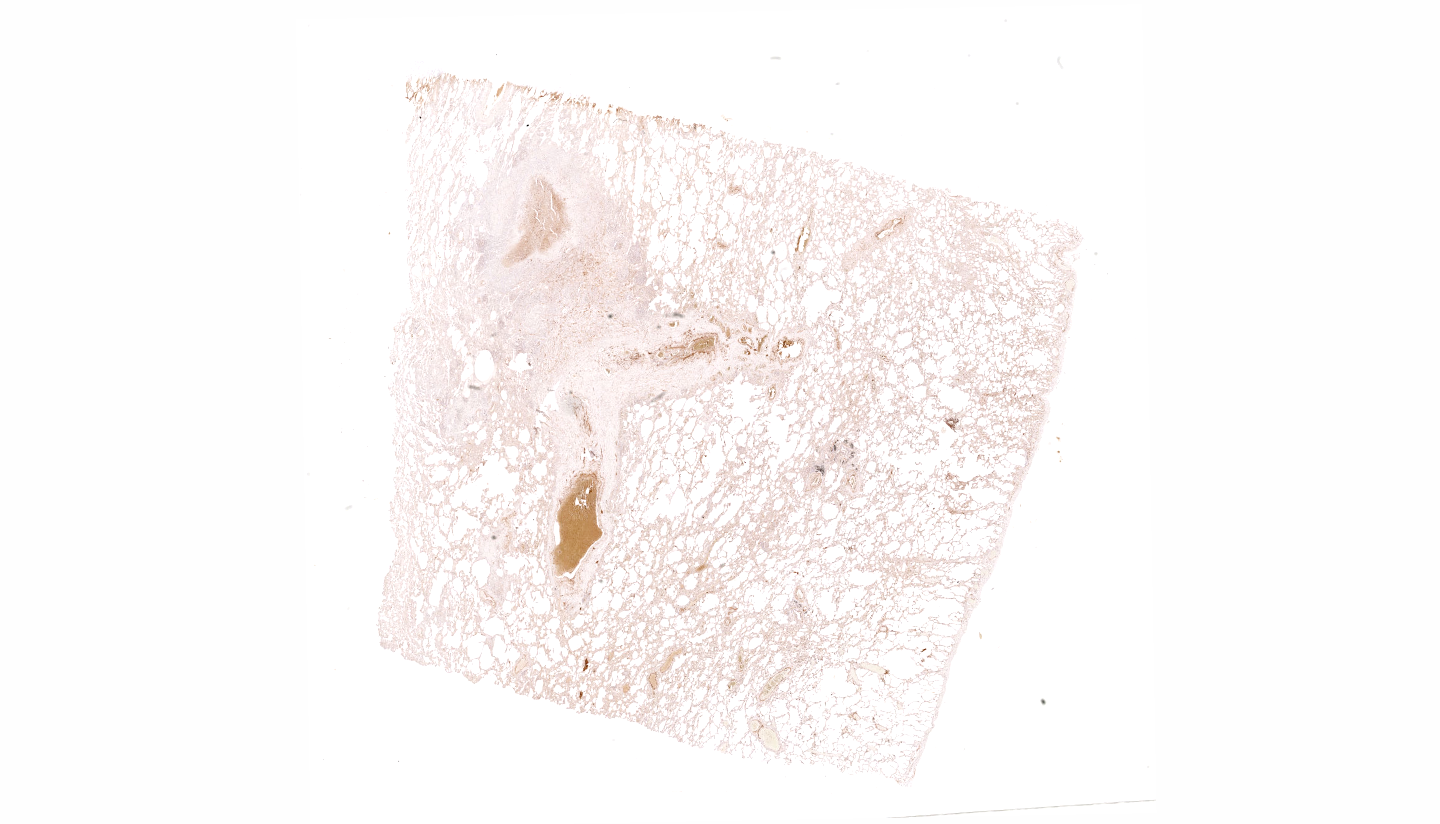

Supplement: Supplementary file 6 — Source data Fig. 3 [file 44321_2026_435_MOESM6_ESM.zip › Figure 3/Figure 3C and D/13462 Citruline H3.tif]

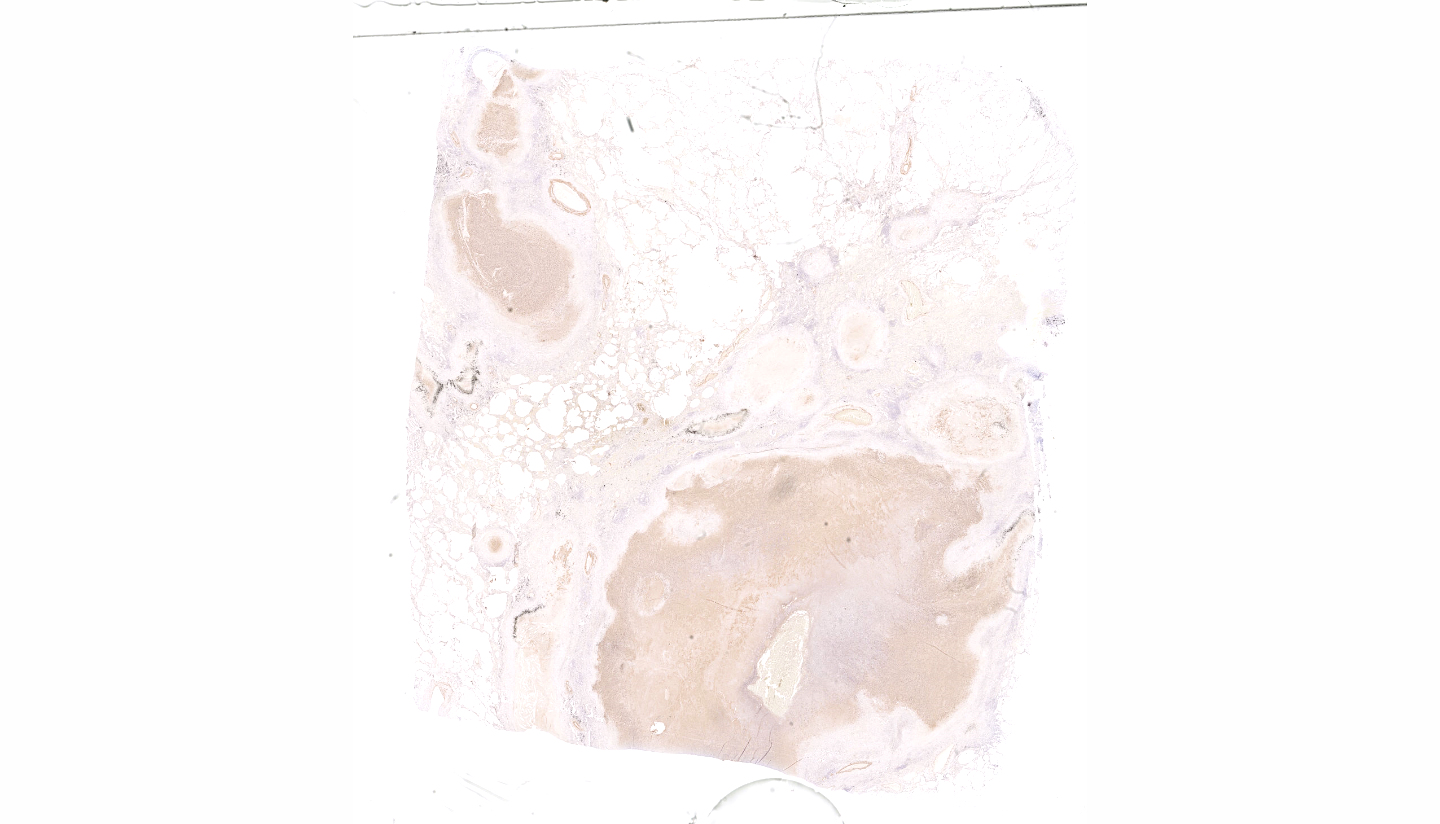

Supplement: Supplementary file 6 — Source data Fig. 3 [file 44321_2026_435_MOESM6_ESM.zip › Figure 3/Figure 3B/20314 09 F Citruline H3 - II.tif]

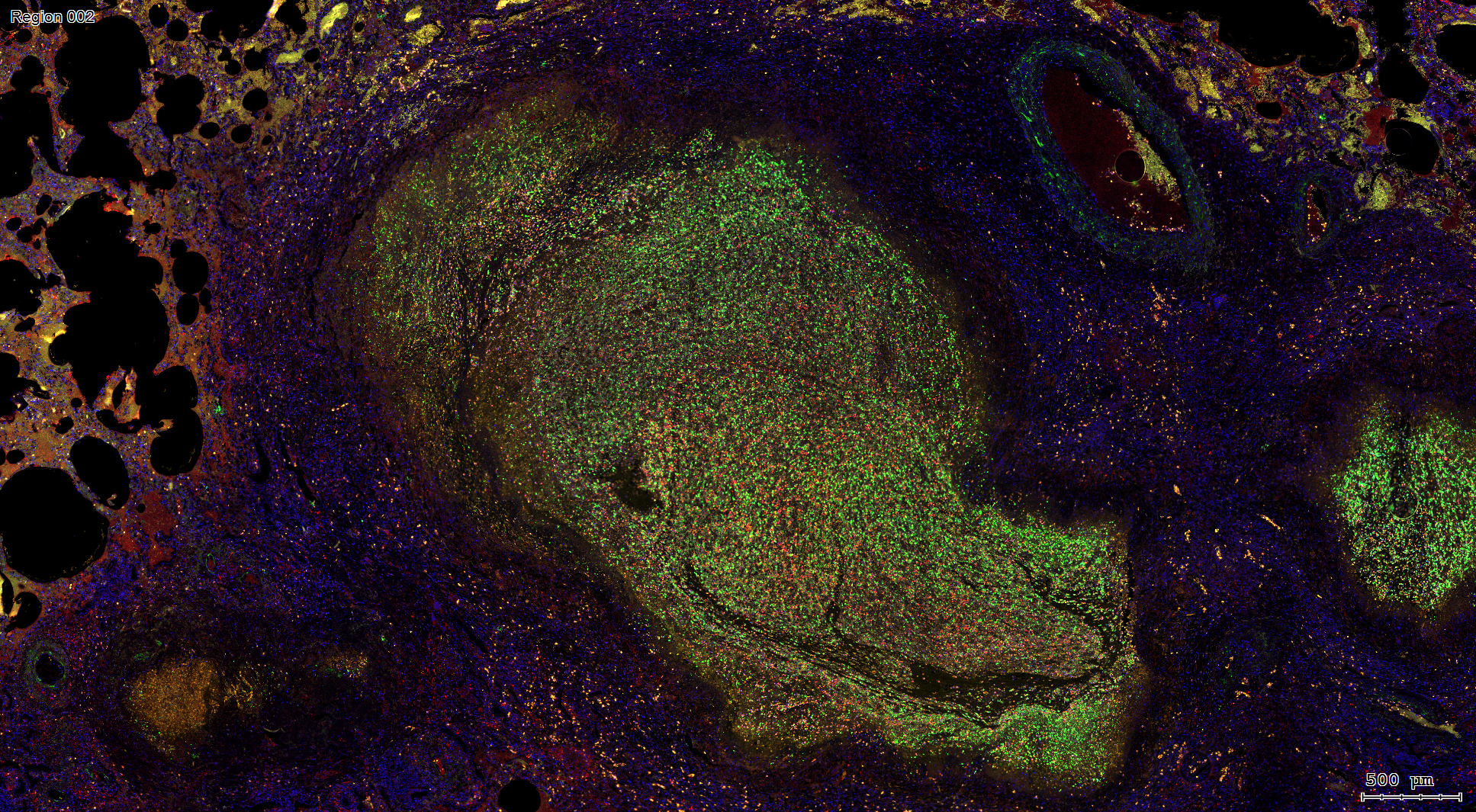

Supplement: Supplementary file 7 — Source data Fig. 4 [file 44321_2026_435_MOESM7_ESM.zip › Figure 4/Figure 4B/CH3 green_ NE red _ MPO yellow_ DAPI blue 22 SEPT 22 COMPOSIT.TIFF]

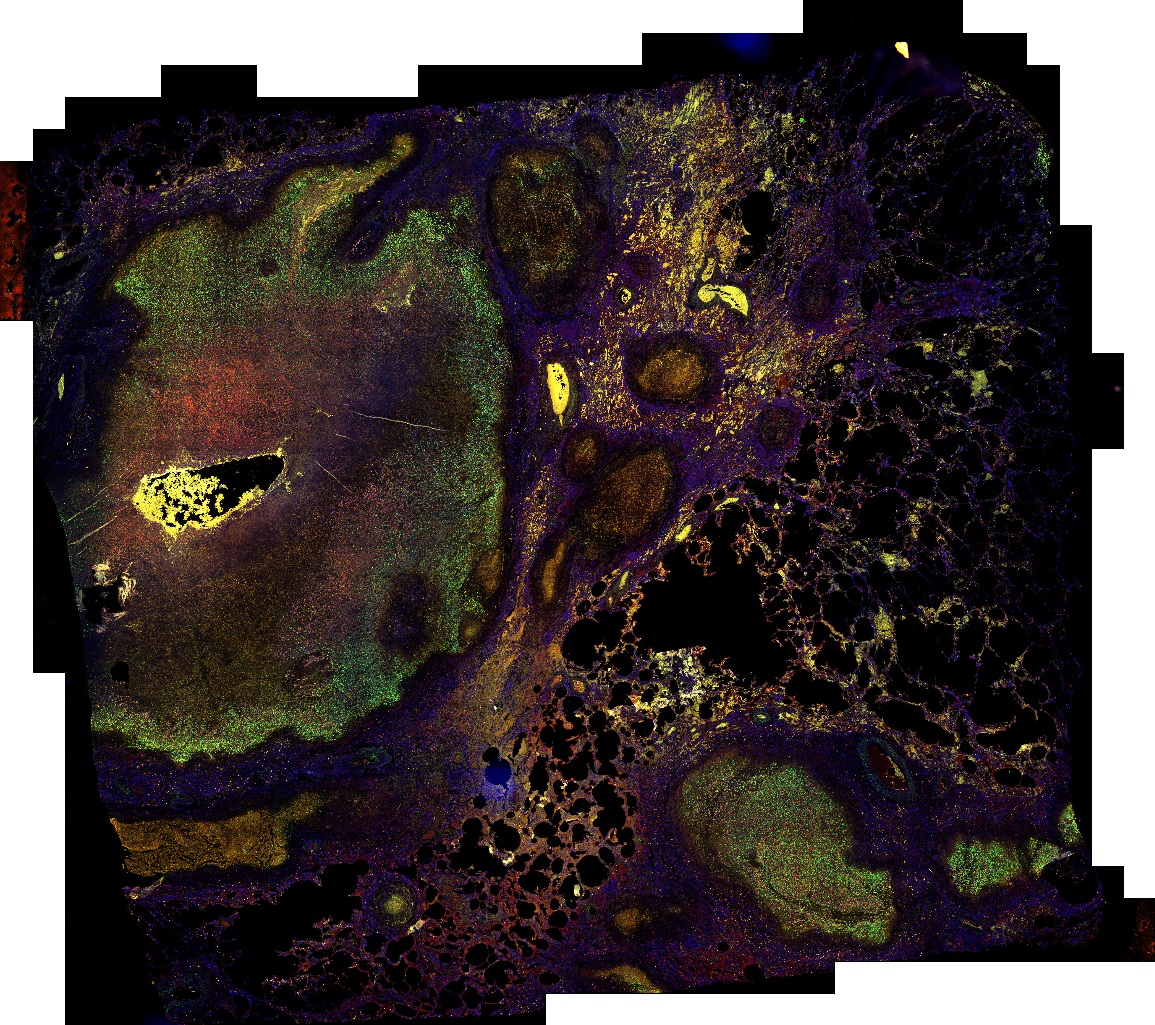

Supplement: Supplementary file 7 — Source data Fig. 4 [file 44321_2026_435_MOESM7_ESM.zip › Figure 4/Figure 4B/Figure 4B _CH3 green_ NE red_ MPO yellow _ DAPI BlueFULL.tiff]

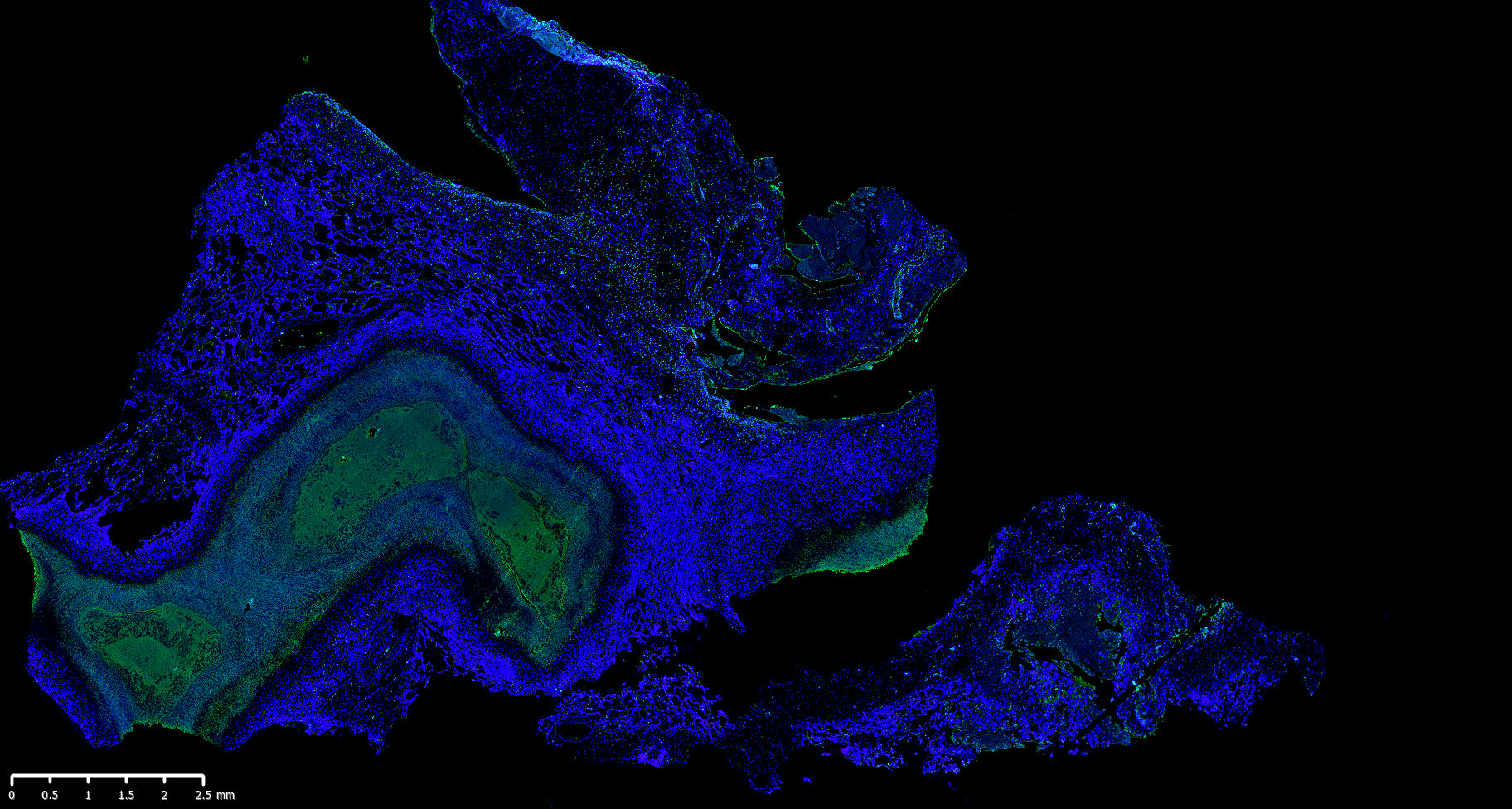

Supplement: Supplementary file 7 — Source data Fig. 4 [file 44321_2026_435_MOESM7_ESM.zip › Figure 4/Figure 4C/Overview pathology_MPO Green_DAPI Blue x0.82.jpg]

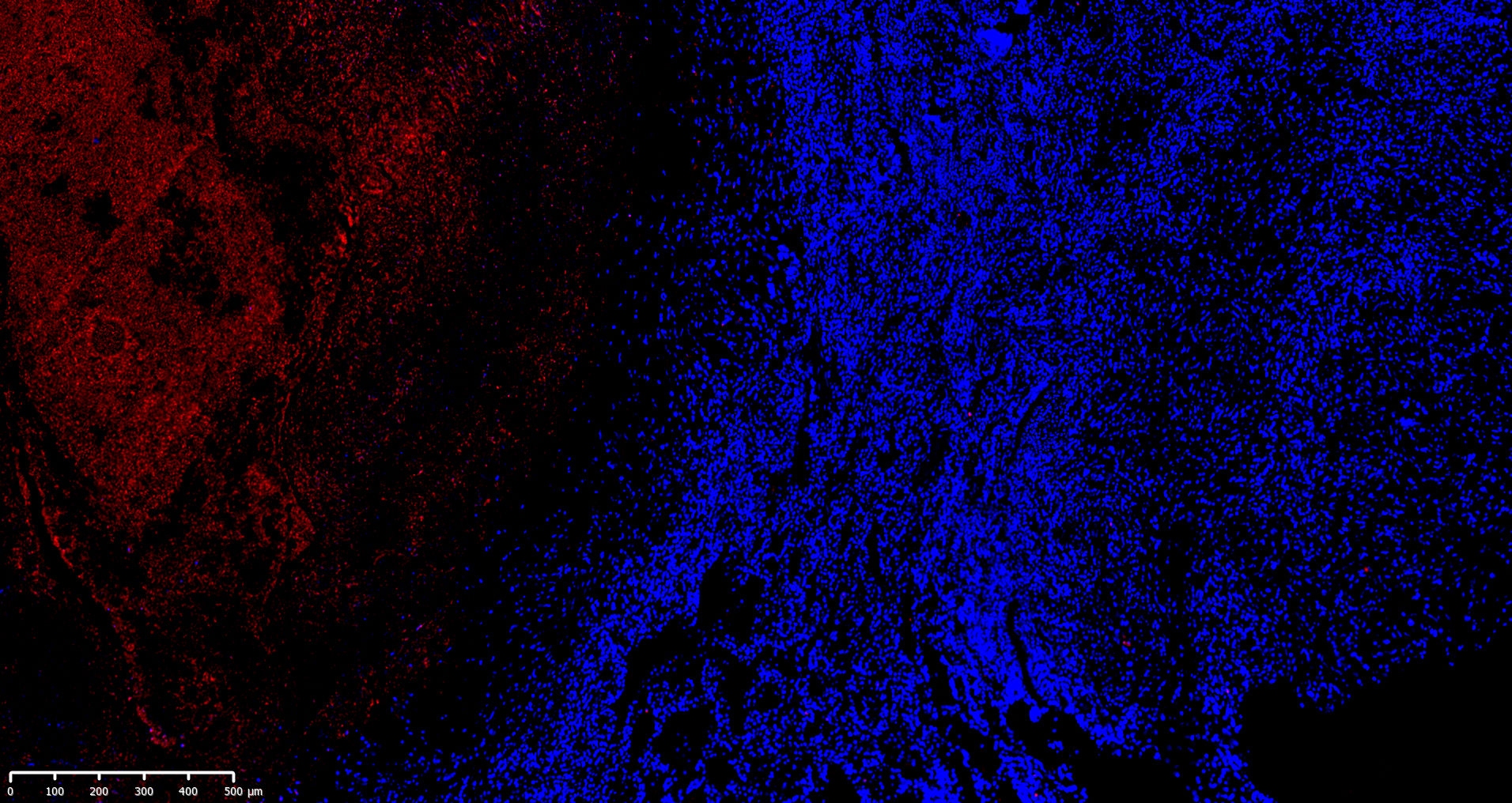

Supplement: Supplementary file 7 — Source data Fig. 4 [file 44321_2026_435_MOESM7_ESM.zip › Figure 4/Figure 4C/NE red _ DAPI blue x5.jpg]

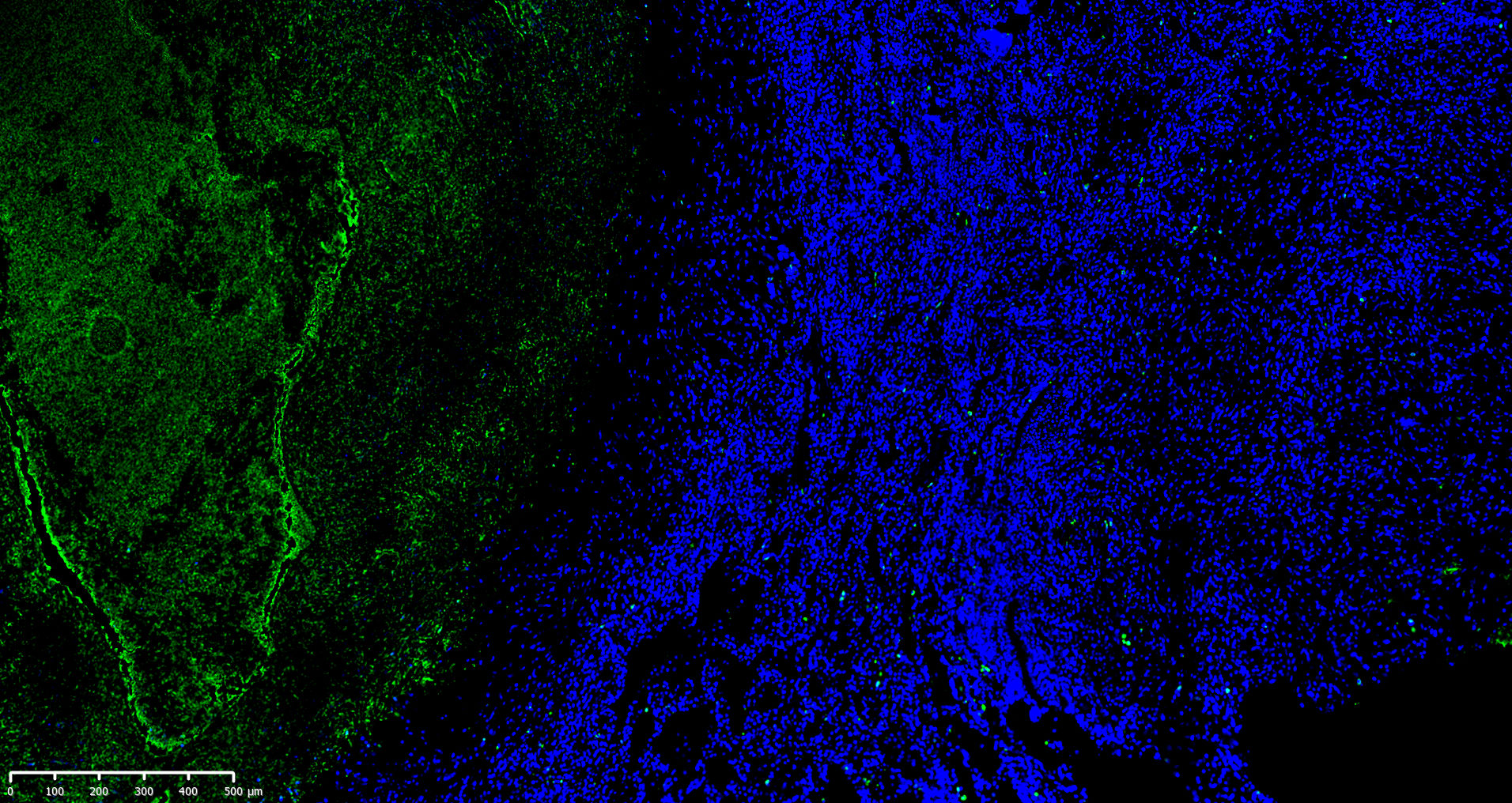

Supplement: Supplementary file 7 — Source data Fig. 4 [file 44321_2026_435_MOESM7_ESM.zip › Figure 4/Figure 4C/MPO green _ DAPI blue x5.jpg]

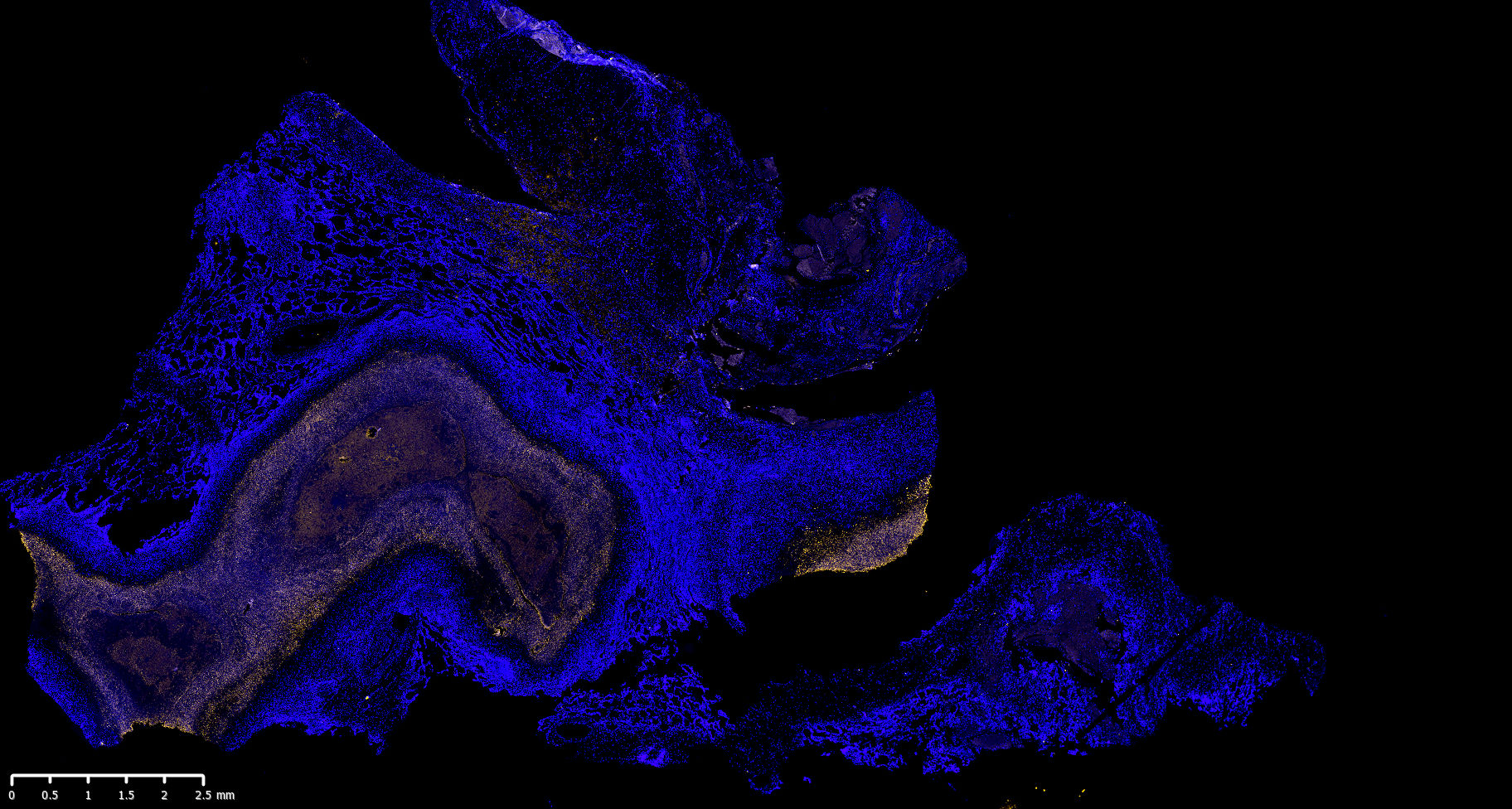

Supplement: Supplementary file 7 — Source data Fig. 4 [file 44321_2026_435_MOESM7_ESM.zip › Figure 4/Figure 4C/Overview pathology_Citruline Yellow_DAPI Blue x0.82.jpg]

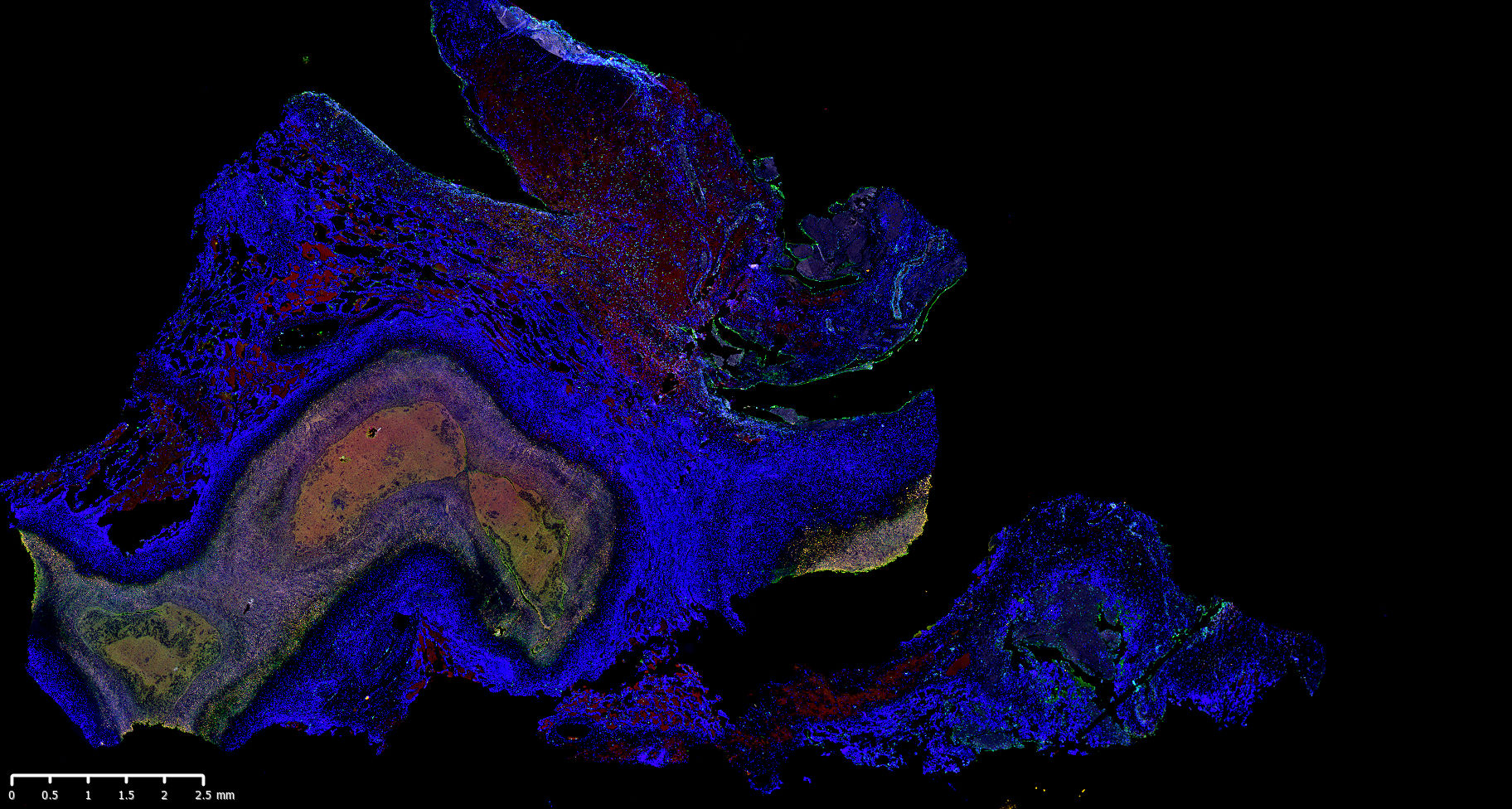

Supplement: Supplementary file 7 — Source data Fig. 4 [file 44321_2026_435_MOESM7_ESM.zip › Figure 4/Figure 4C/Overview pathology_MPO Green_Citruline Yellow_NE red_DAPI Blue x0.82.jpg]

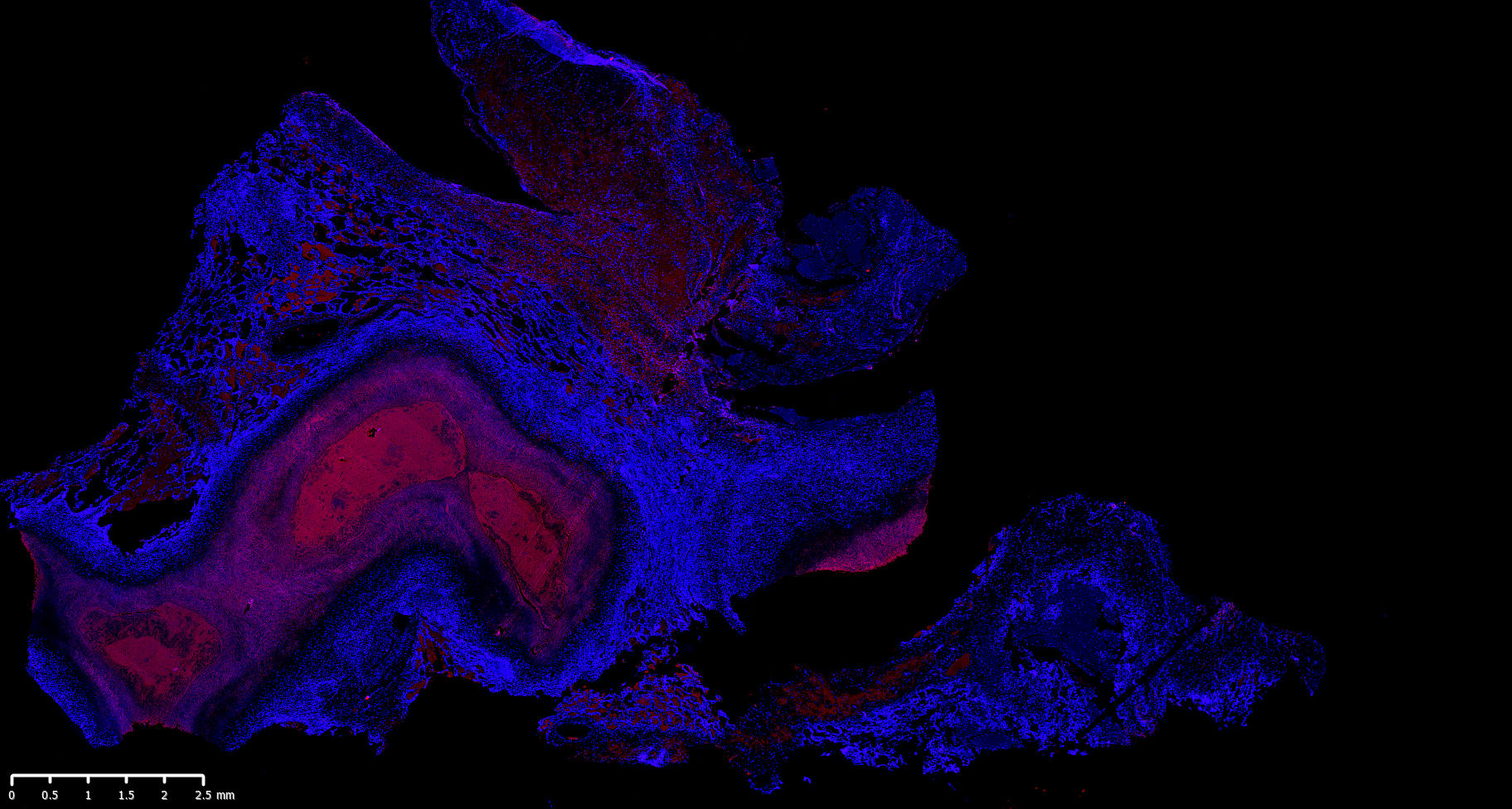

Supplement: Supplementary file 7 — Source data Fig. 4 [file 44321_2026_435_MOESM7_ESM.zip › Figure 4/Figure 4C/Overview pathology_NE red_DAPI Blue x0.82.jpg]

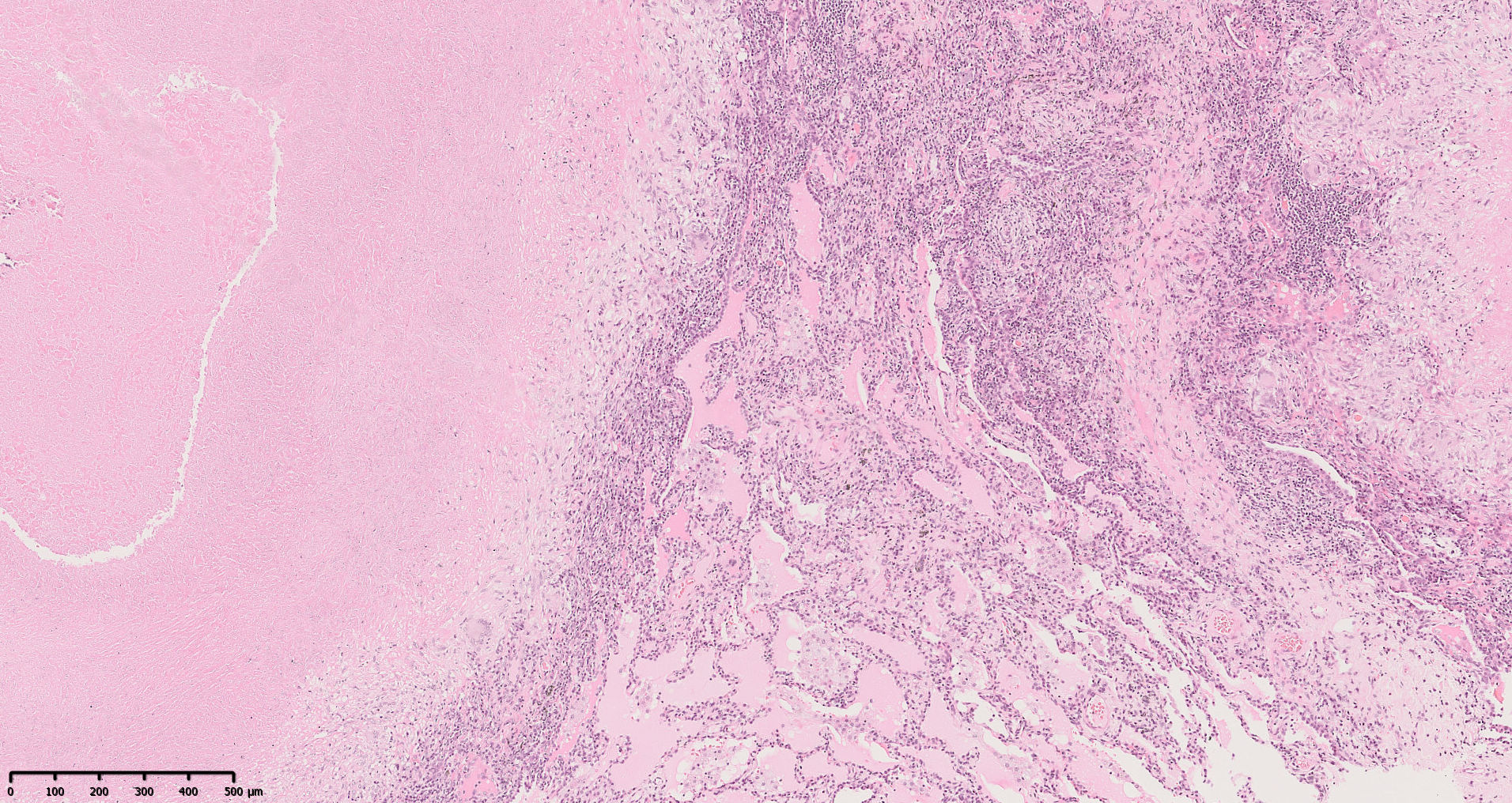

Supplement: Supplementary file 7 — Source data Fig. 4 [file 44321_2026_435_MOESM7_ESM.zip › Figure 4/Figure 4C/H&E zoomed lesion 1 x5.jpg]

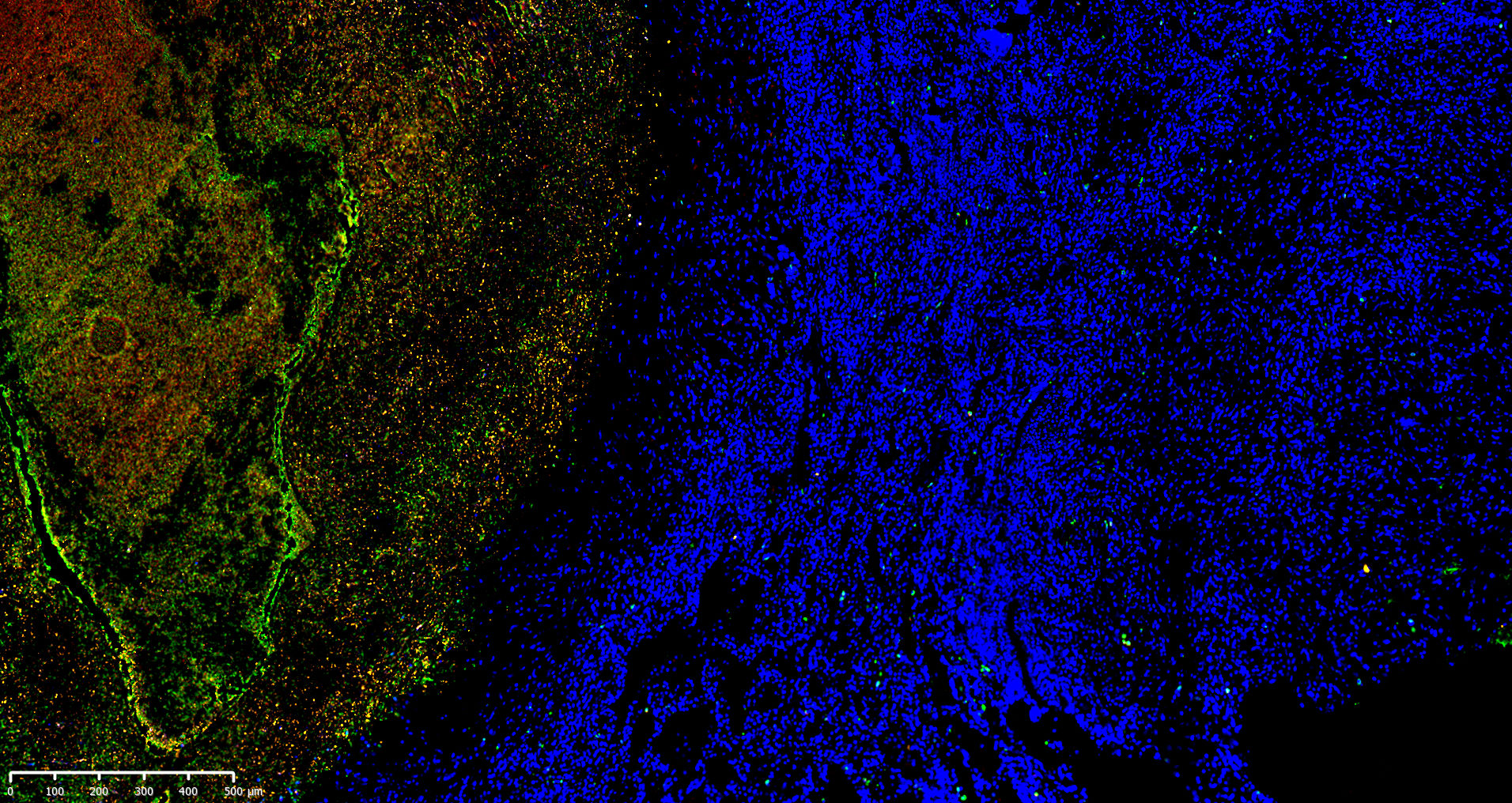

Supplement: Supplementary file 7 — Source data Fig. 4 [file 44321_2026_435_MOESM7_ESM.zip › Figure 4/Figure 4C/MPO green_ C.H3 Yellow_ NE red _ DAPI blue x5.jpg]

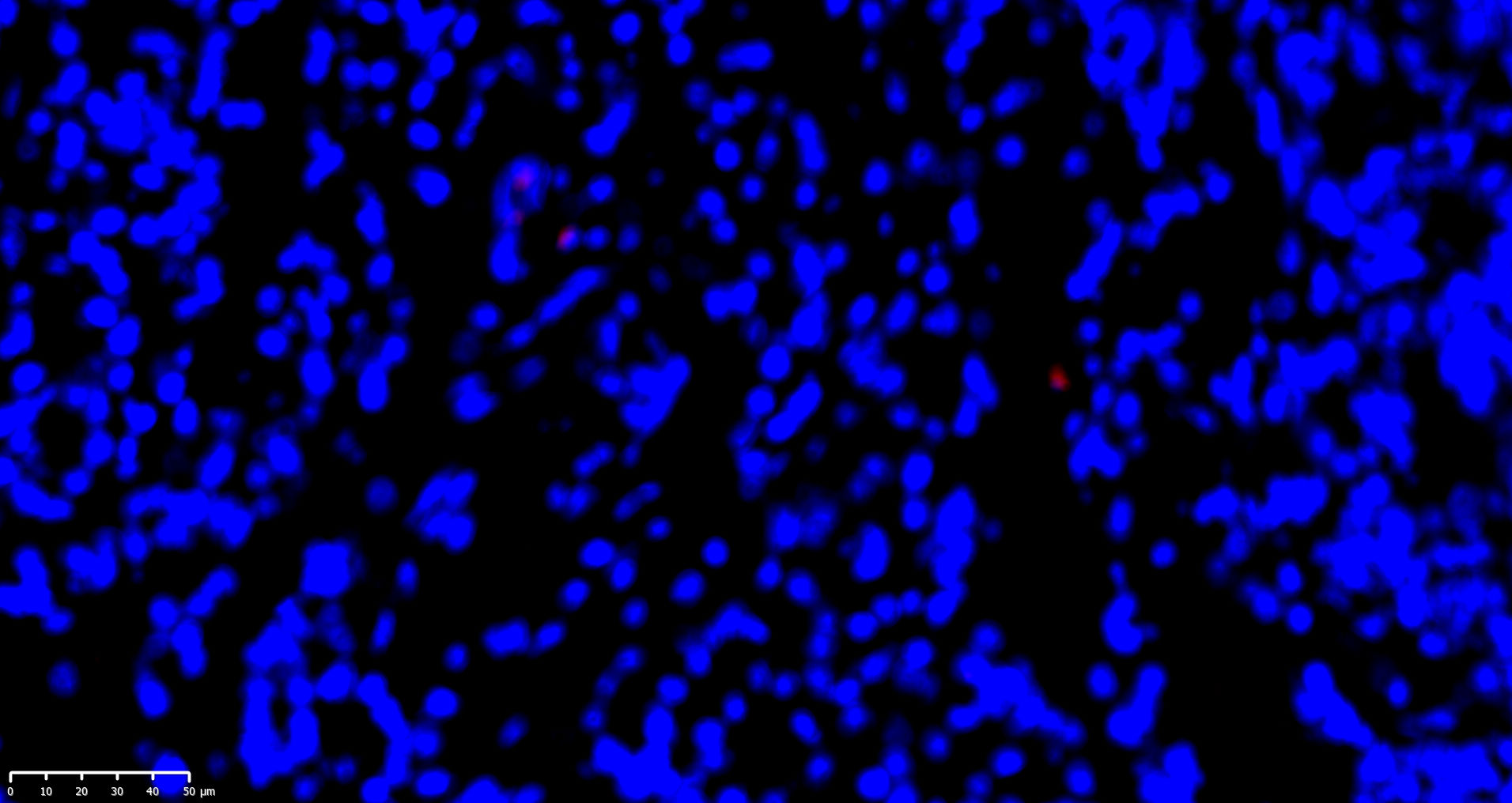

Supplement: Supplementary file 7 — Source data Fig. 4 [file 44321_2026_435_MOESM7_ESM.zip › Figure 4/Figure 4D/Alveolar space/1. AS _ NE red _ DAPI blue x40.jpg]

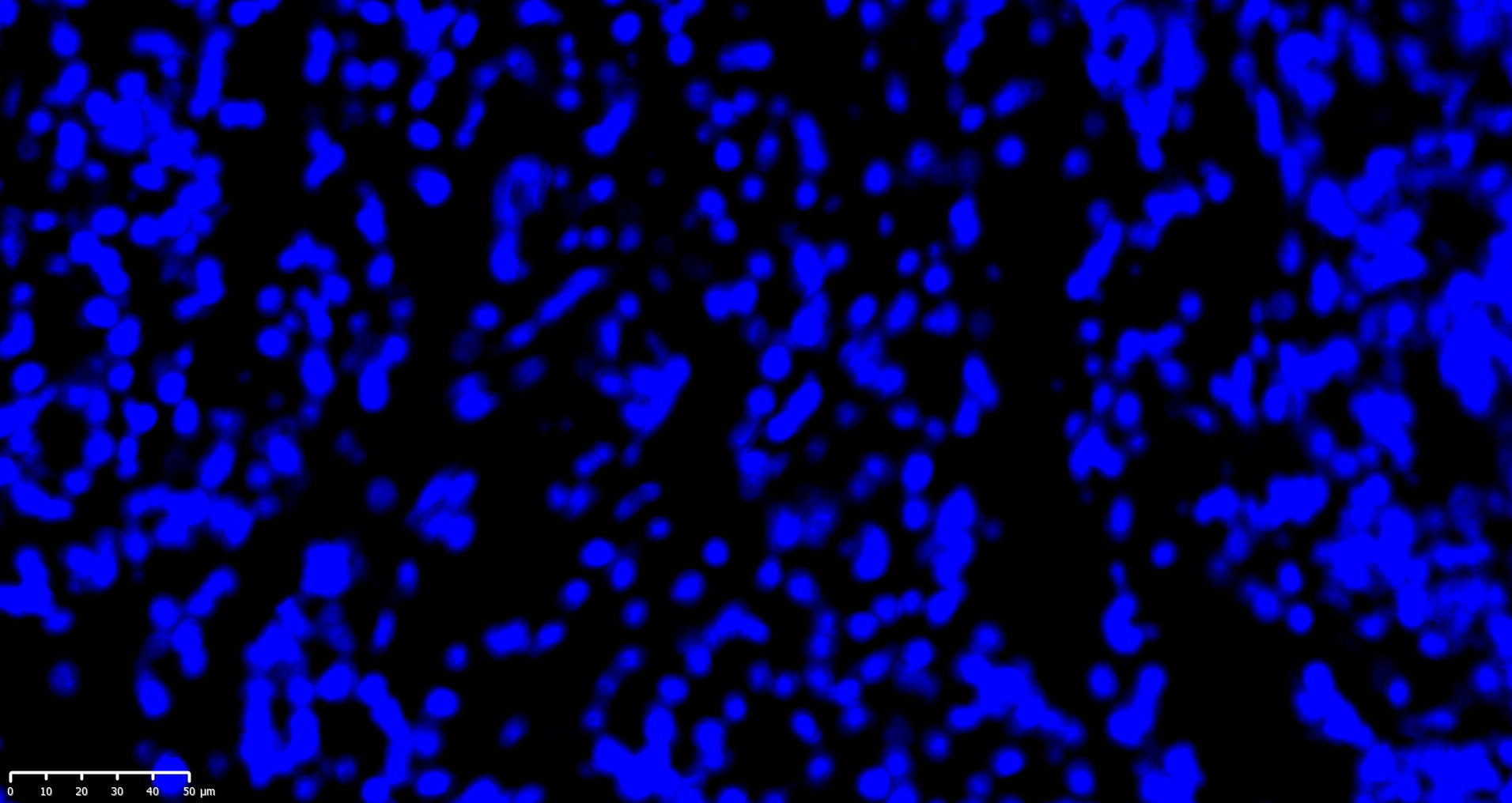

Supplement: Supplementary file 7 — Source data Fig. 4 [file 44321_2026_435_MOESM7_ESM.zip › Figure 4/Figure 4D/Alveolar space/1. AS _ C.H3 Yellow_ DAPI blue x40.jpg]

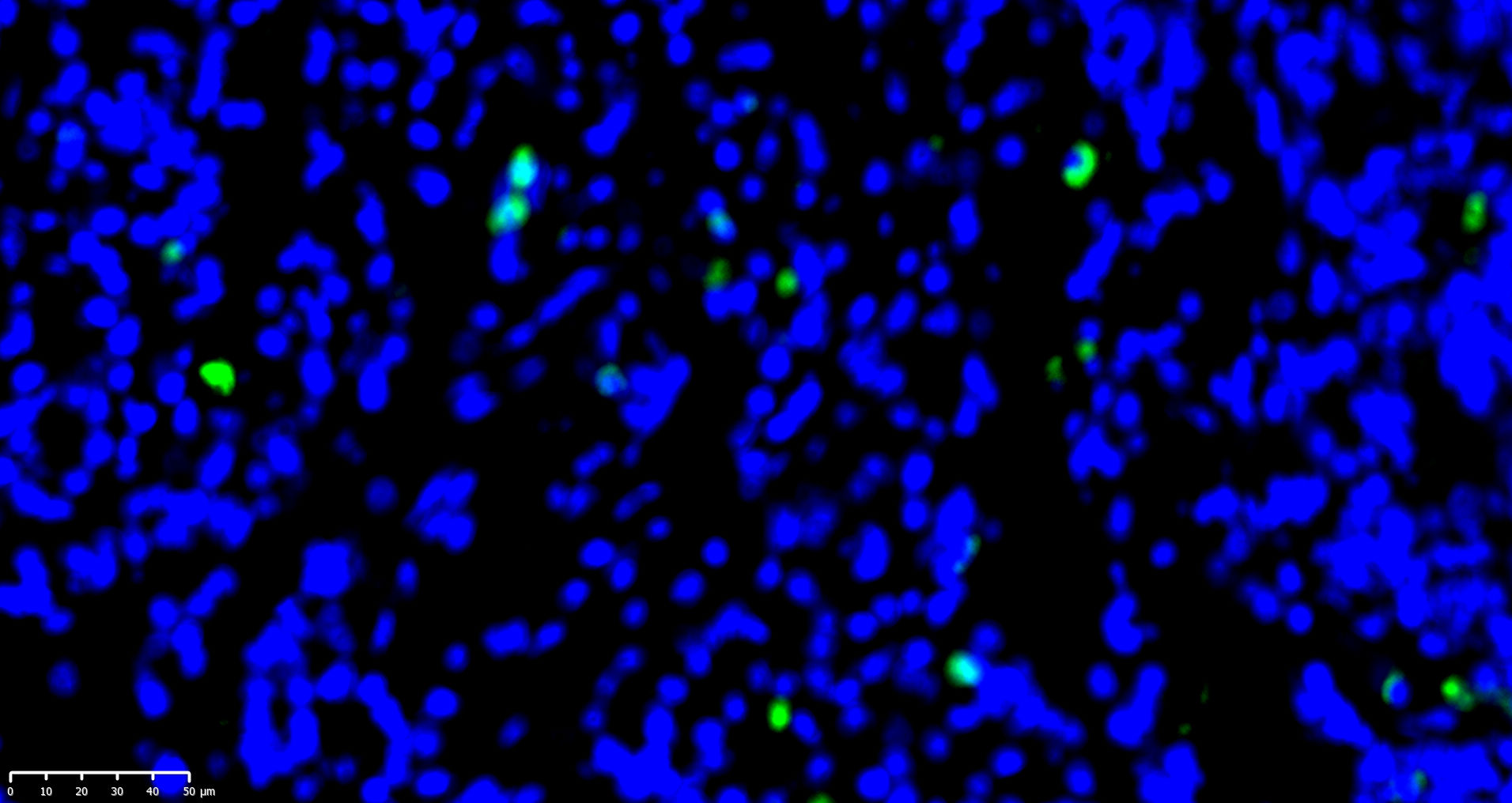

Supplement: Supplementary file 7 — Source data Fig. 4 [file 44321_2026_435_MOESM7_ESM.zip › Figure 4/Figure 4D/Alveolar space/1. AS _ MPO green _ DAPI blue x40.jpg]

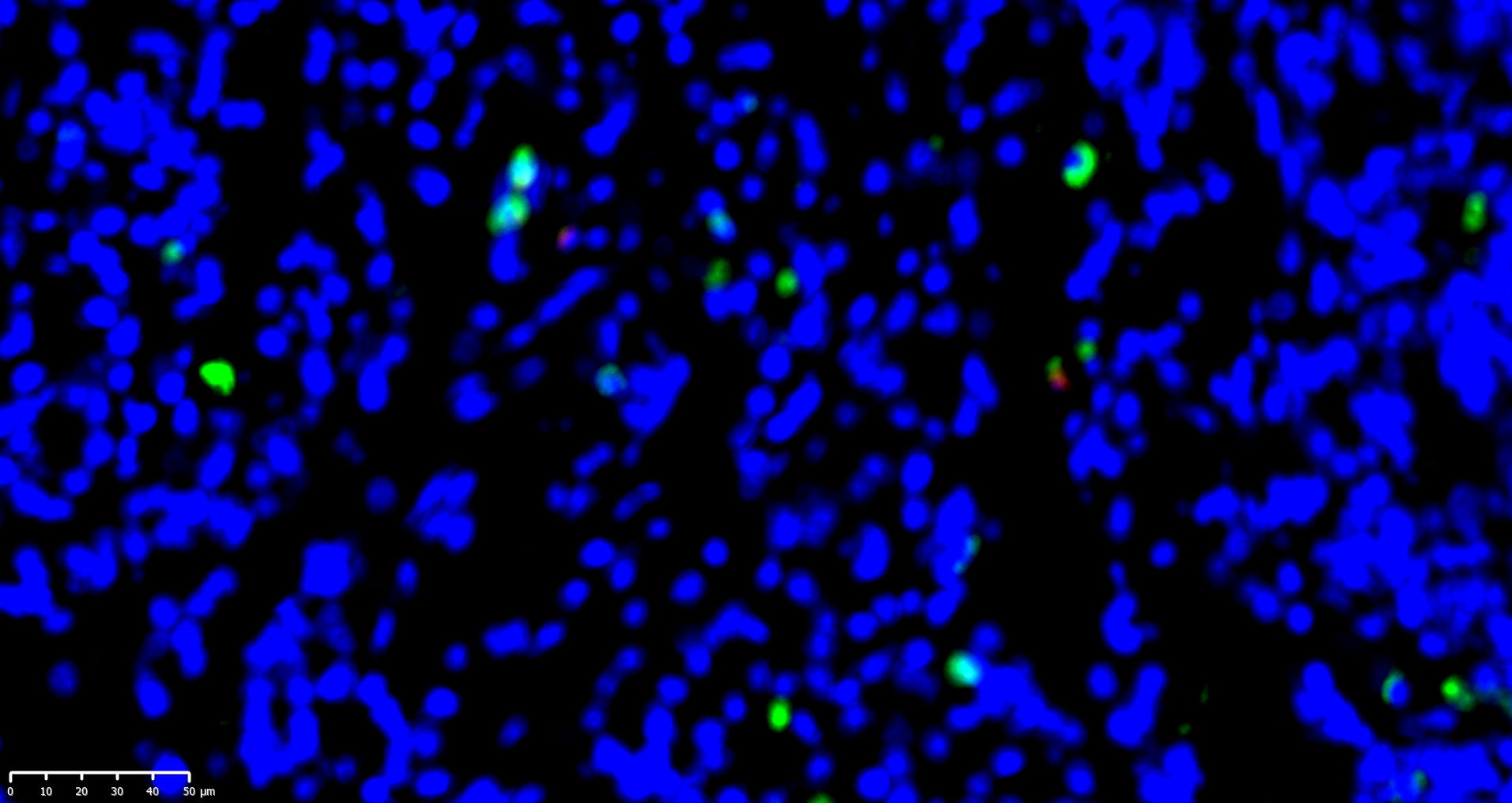

Supplement: Supplementary file 7 — Source data Fig. 4 [file 44321_2026_435_MOESM7_ESM.zip › Figure 4/Figure 4D/Alveolar space/1. AS _ MPO green_ C.H3 Yellow_ NE red _ DAPI blue x40.jpg]

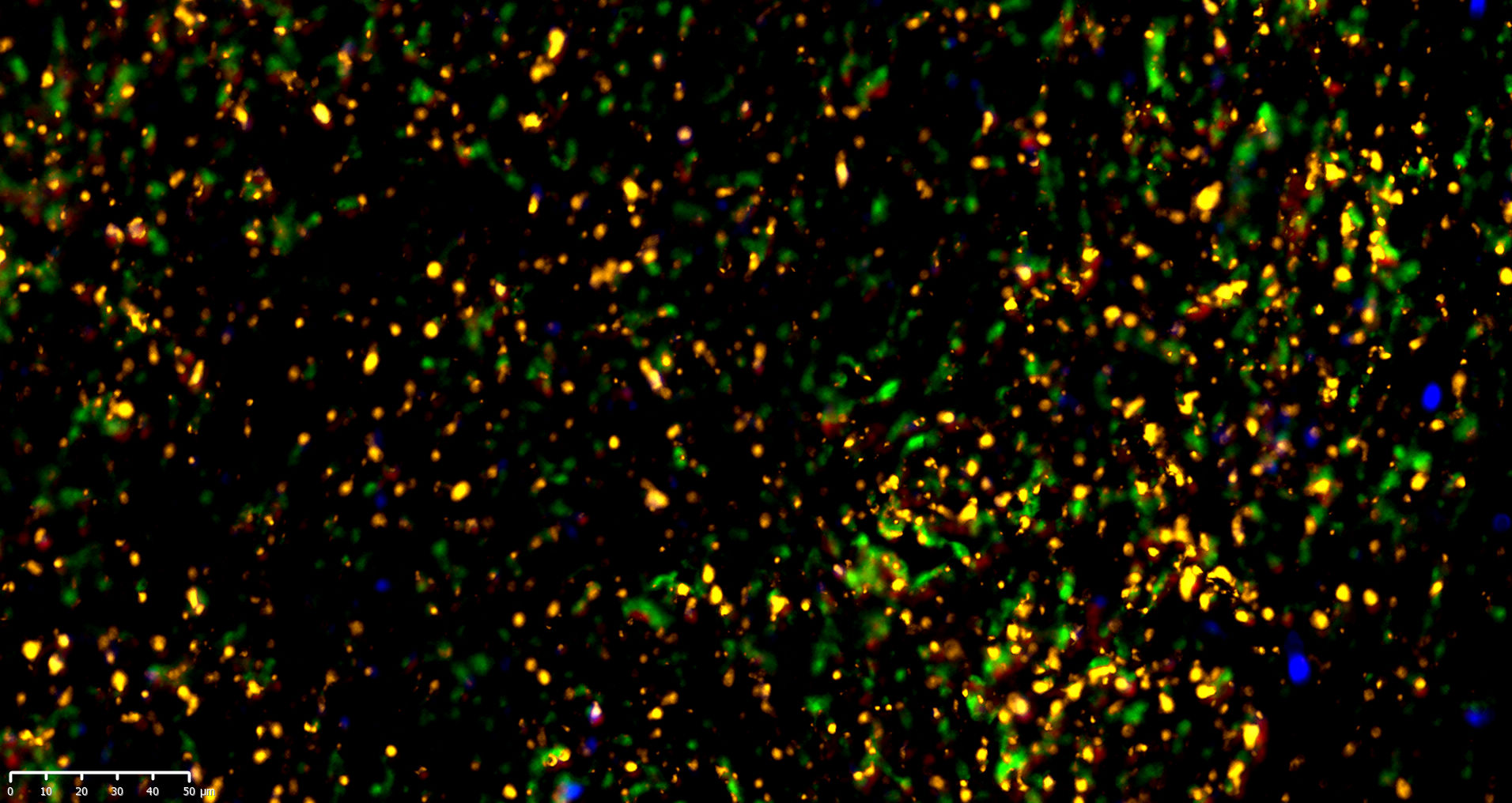

Supplement: Supplementary file 7 — Source data Fig. 4 [file 44321_2026_435_MOESM7_ESM.zip › Figure 4/Figure 4D/Necrotic region/NR _ MPO green_ C.H3 Yellow_ NE red _ DAPI blue x40.jpg]

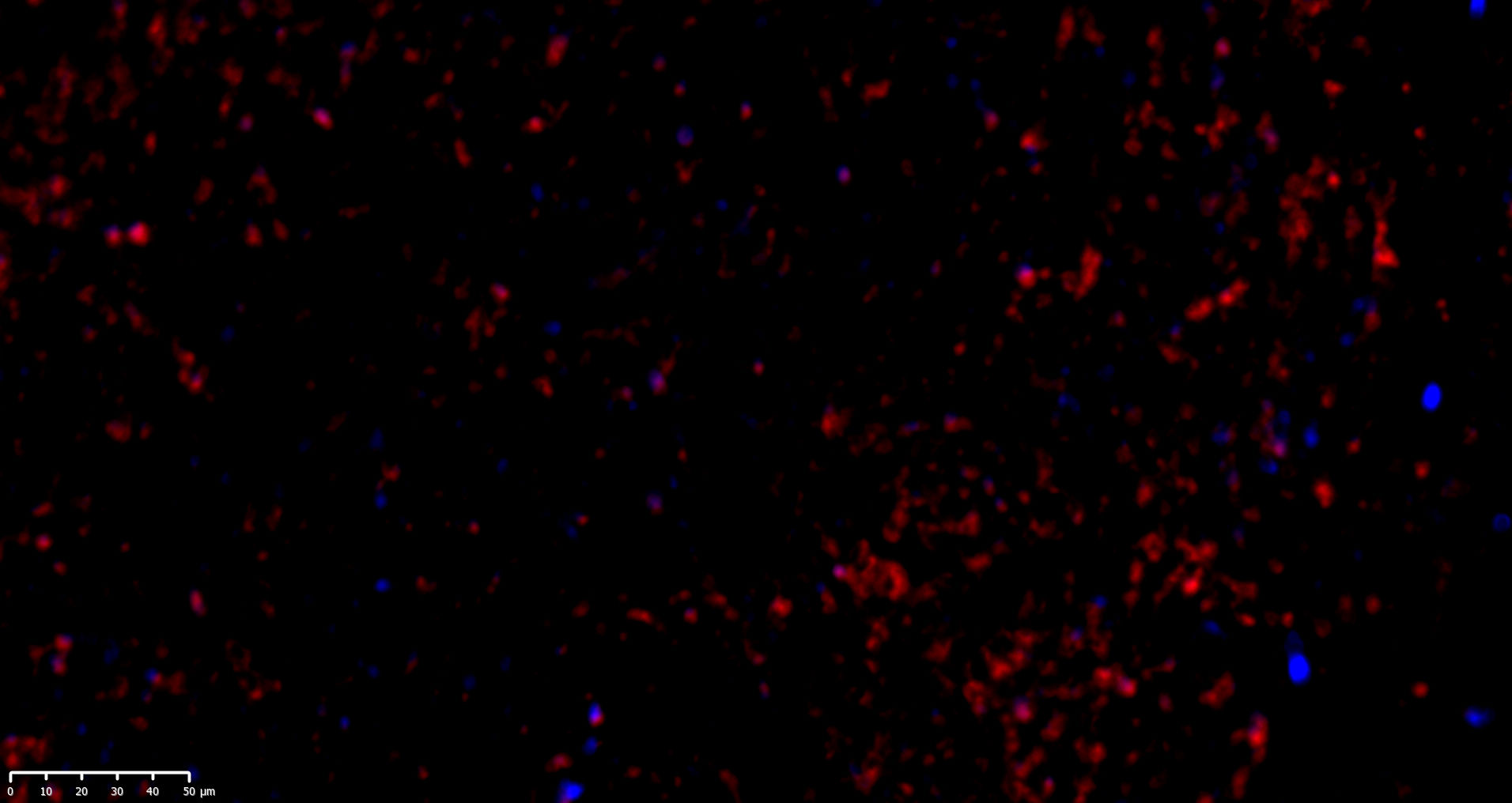

Supplement: Supplementary file 7 — Source data Fig. 4 [file 44321_2026_435_MOESM7_ESM.zip › Figure 4/Figure 4D/Necrotic region/NR _ NE red _ DAPI blue x40.jpg]

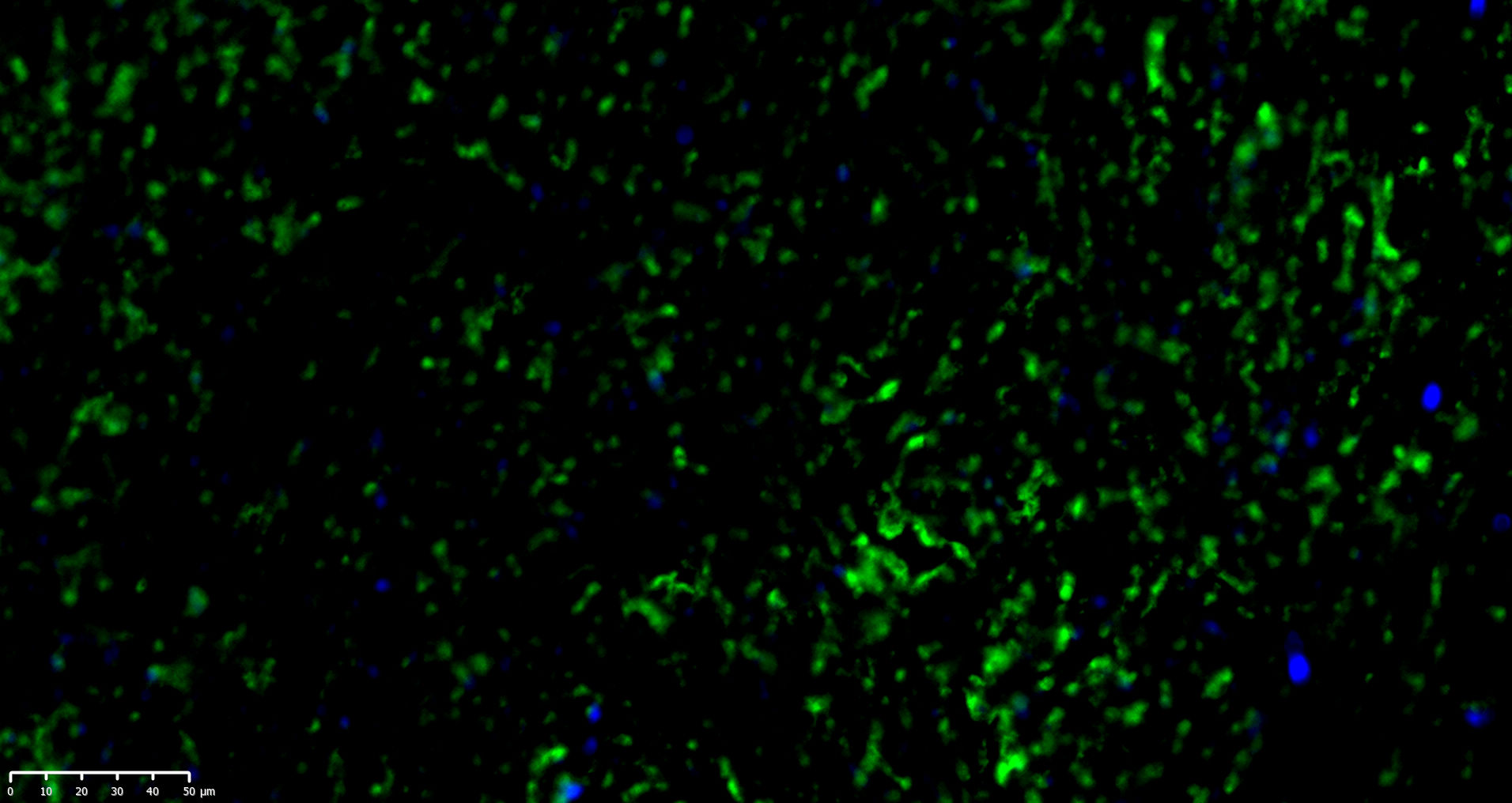

Supplement: Supplementary file 7 — Source data Fig. 4 [file 44321_2026_435_MOESM7_ESM.zip › Figure 4/Figure 4D/Necrotic region/NR _ MPO green_ DAPI blue x40.jpg]

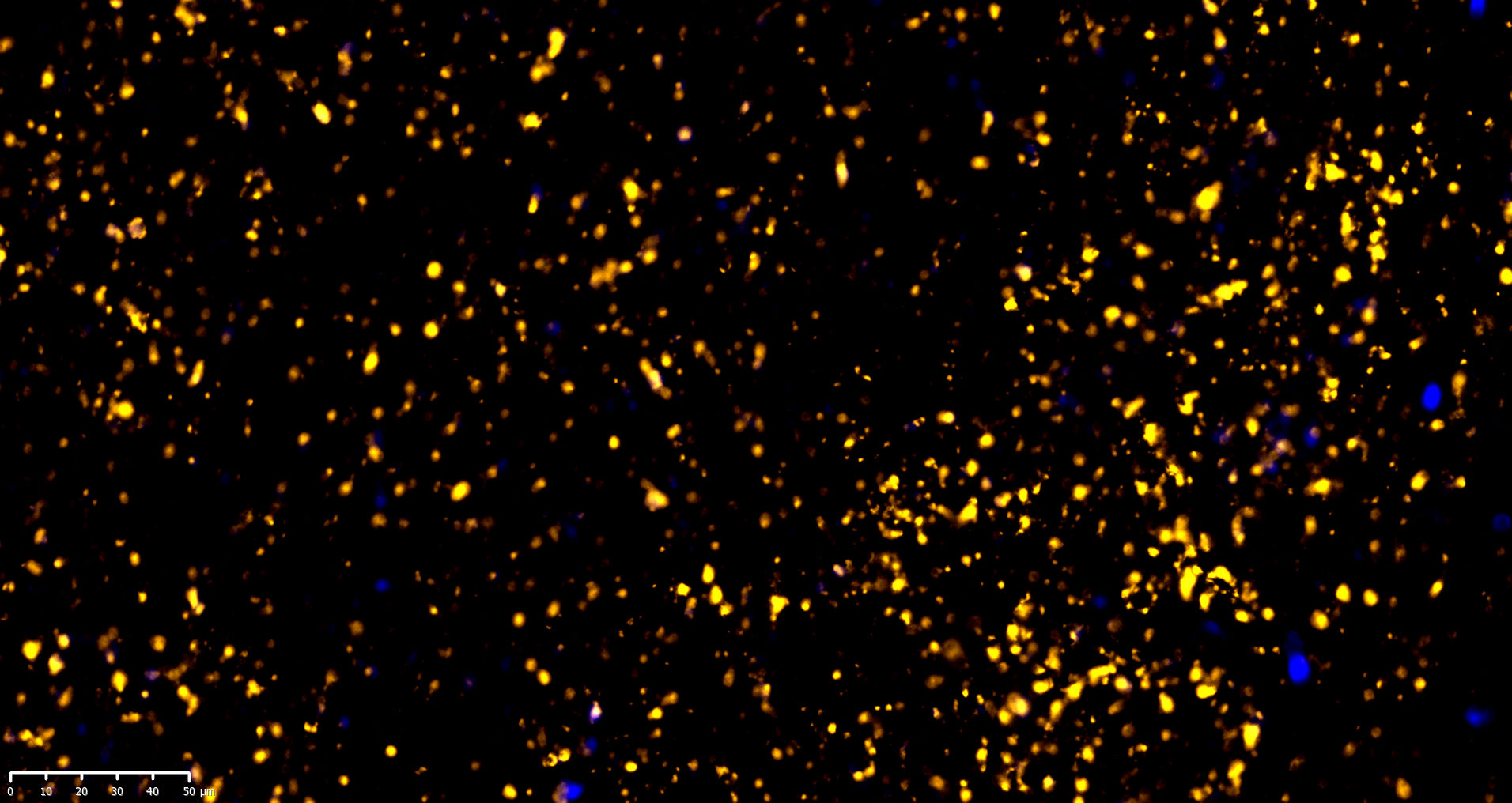

Supplement: Supplementary file 7 — Source data Fig. 4 [file 44321_2026_435_MOESM7_ESM.zip › Figure 4/Figure 4D/Necrotic region/NR _ C.H3 Yellow_ DAPI blue x40.jpg]
