# Supplementary material for: NETosis associates with human TB lung tissue destruction and disease pathogenesis
Source: EMBO Mol Med. 2026 Jun 2;18(7):2547–72. doi: 10.1038/s44321-026-00435-3 (PMC13365388; doi:10.1038/s44321-026-00435-3)
Supplement: Supplementary file 10 — Source data Fig. 7 [file 44321_2026_435_MOESM10_ESM.zip › Figure 7/Source of data in Figure 7.docx]

Transcriptional profiling of neutrophil related genes in **Figure 7** was analyzed using datasets previously published in the work cited below:

*Pollara, G., Turner, C.T., Rosenheim, J., Chandran, A., Bell, L.C., Khan, A., Patel, A.,*

*Peralta, L.F., Folino, A., Akarca, A., et al. (2021). Exaggerated IL-17A activity in human in*

*vivo recall responses discriminates active tuberculosis from latent infection and cured disease. Sci. Transl. Med. 13, eabg7673. https://doi.org/10.1126/scitranslmed.abg7673.*
