# Supplementary material for: NETosis associates with human TB lung tissue destruction and disease pathogenesis
Source: EMBO Mol Med. 2026 Jun 2;18(7):2547–72. doi: 10.1038/s44321-026-00435-3 (PMC13365388; doi:10.1038/s44321-026-00435-3)
Supplement: Supplementary file 11 — Expanded View Figures [file 44321_2026_435_MOESM11_ESM.pdf]

Expanded View Figures

Figure EV1. Neutrophil-specific proteins are enriched and colocalize in necrotic caseum.

(**A**) MPO and (**B**) NE were abundant in early caseum (containing cellular debris) and necrotizing caseum. (**C**) NCF had a positive stain in caseum, which was more pronounced in necrotic regions. (**D**) NOX2 was more enriched in the borders of necrotizing caseum. IF staining of MPO (green) and NE (red) in the caseum (**E**). Scale bar represents 100, 500, and 1 mm.

 © The Author(s)

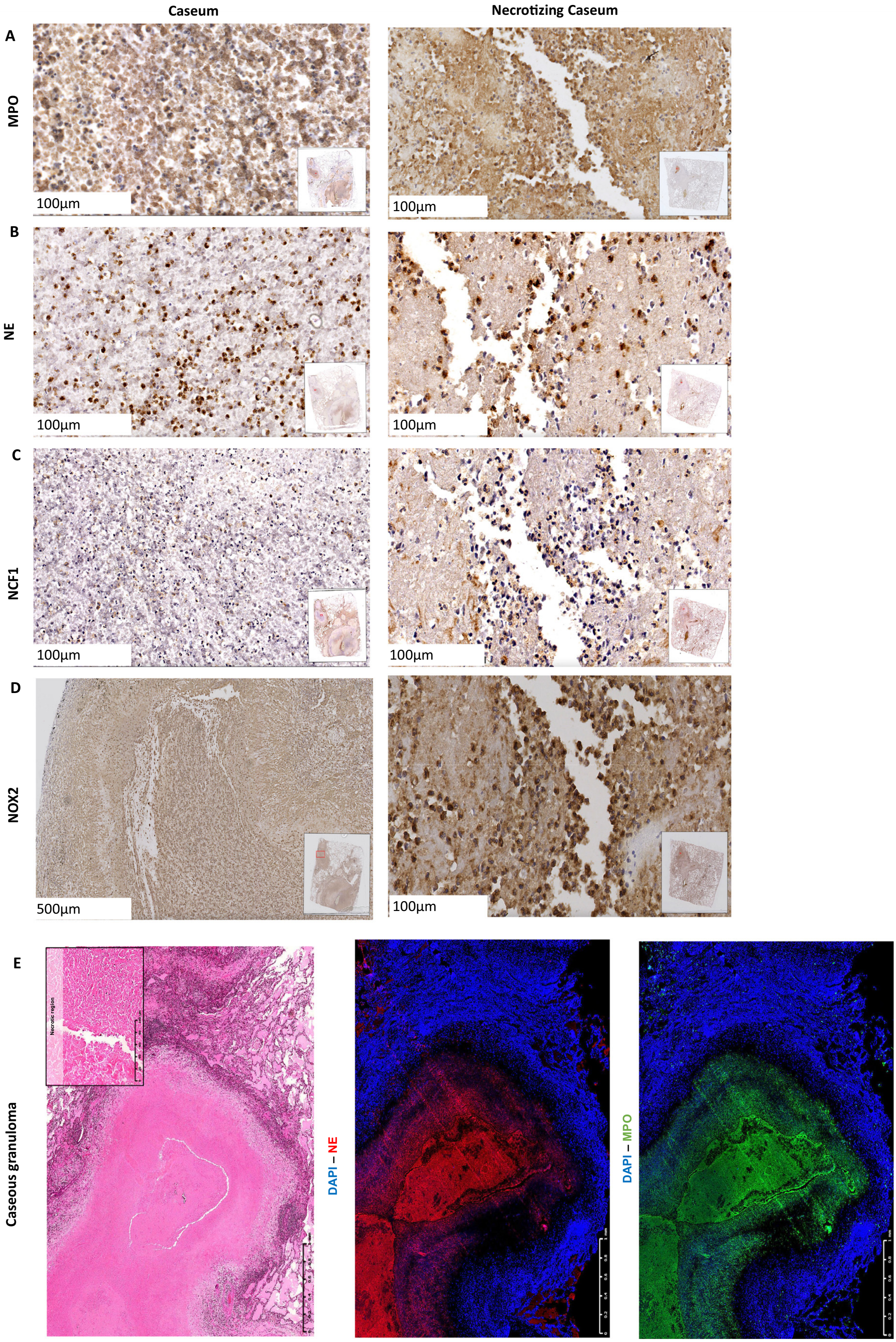
Caseum
Necrotizing Caseum
A
MPO
100µm
100µm
B
NE
100µm
100µm
C
NCF1
100µm
100µm
D
NOX2
500µm
100µm
E
Caseous granuloma
Necrotic region
DAPI – NE
DAPI – MPO

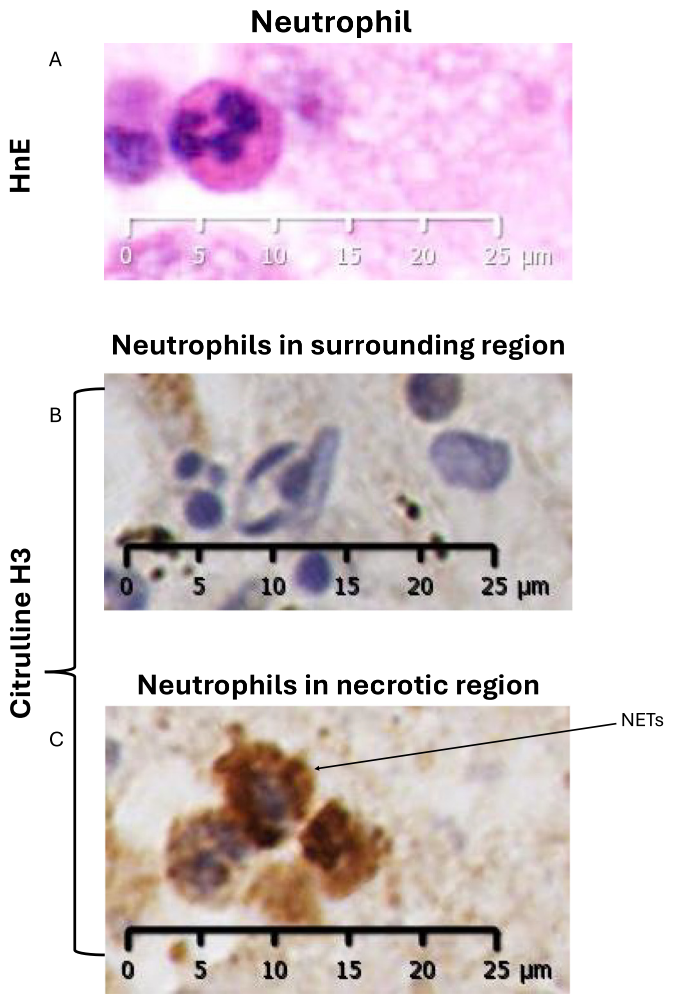

Figure EV2. NETs are more abundant in the caseum than in cellular regions.

Lung tissue samples were stained with hemolysin and eosin (**A**). IHC-stained tissue showing Citrulline H3-negative neutrophils in the cellular region of TB granuloma (**B**). IHC-stained tissue showing neutrophils extruding NETs in the caseum of TB granulomas (**C**). Scale bar = 25 µm.

 © The Author(s)

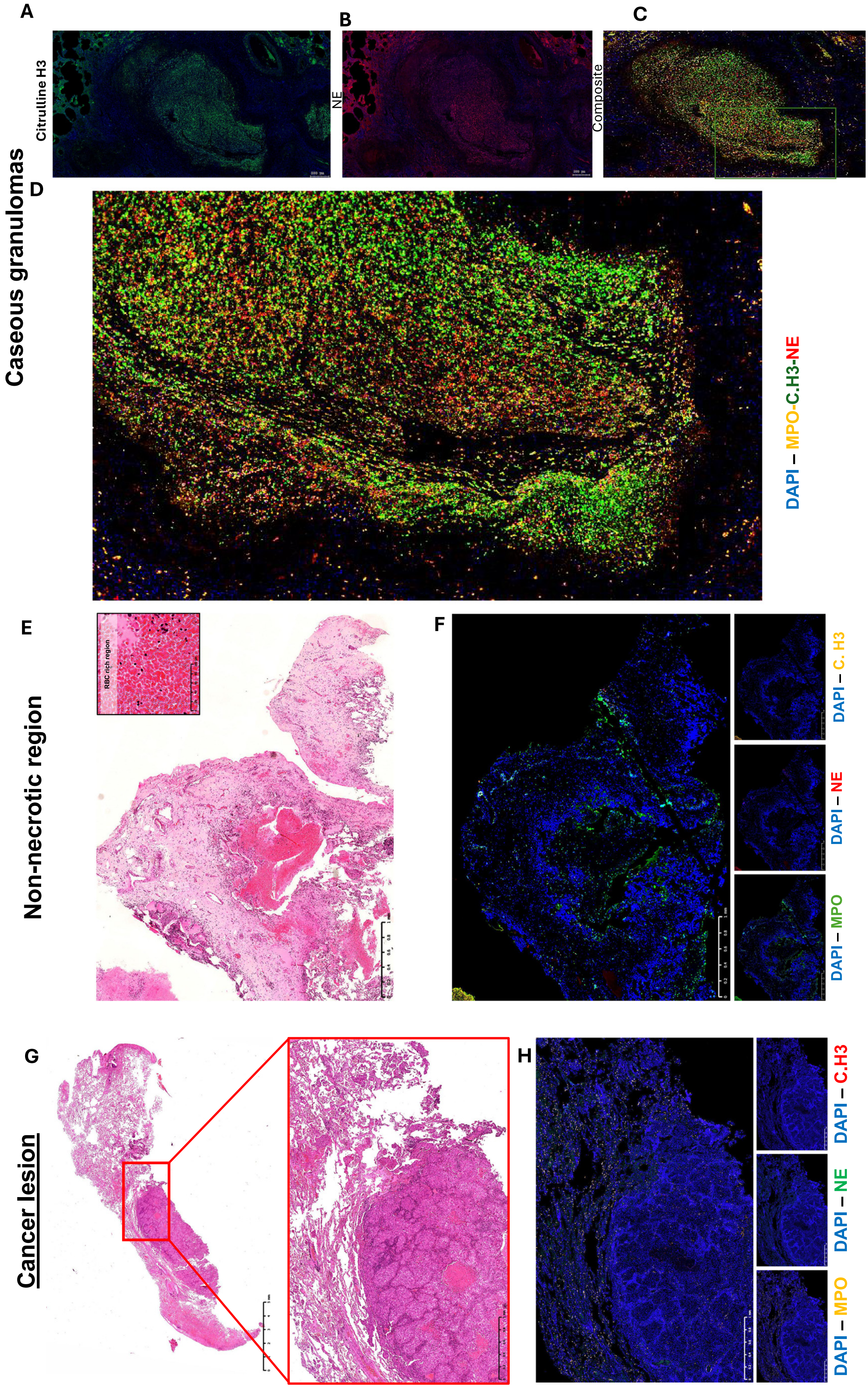
A
B
C
Citrulline H3
NE
Composite
Caseous granulomas
D
DAPI – MPO-C.H3-NE
E
RBC-rich region
F
DAPI – C. H3
DAPI – NE
DAPI – MPO
Non-necrotic region
G
H
Cancer lesion
DAPI – C.H3
DAPI – NE
DAPI – MPO

Figure EV3. Neutrophil elastase colocalizes with NETs in the granuloma caseum.

Immunofluorescence (IF) staining showing citrulline H3 (green) (**A**), NE (Red) (**B**), and composite citrulline H3, NE, and MPO (yellow) co-staining on the same caseous granuloma (**C**), which is zoomed in to show colocalization of NE and citrulline H3 (**D**). H&E staining of human TB lung illustrating a non-necrotic granuloma (**E**). IF staining of MPO (Green), NE (Red) and citrulline H3 (Yellow) on non-necrotic granuloma **(F)**. H&E staining of control human adenocarcinoma associated lung pathology (**G**). IF staining of the adenocarcinoma lesion showing MPO (Yellow), NE (Green), and citrulline H3 (Red). Scale bars represent 5 mm, 1 mm, 500 μm, and 50 μm as indicated on each image.

 © The Author(s)

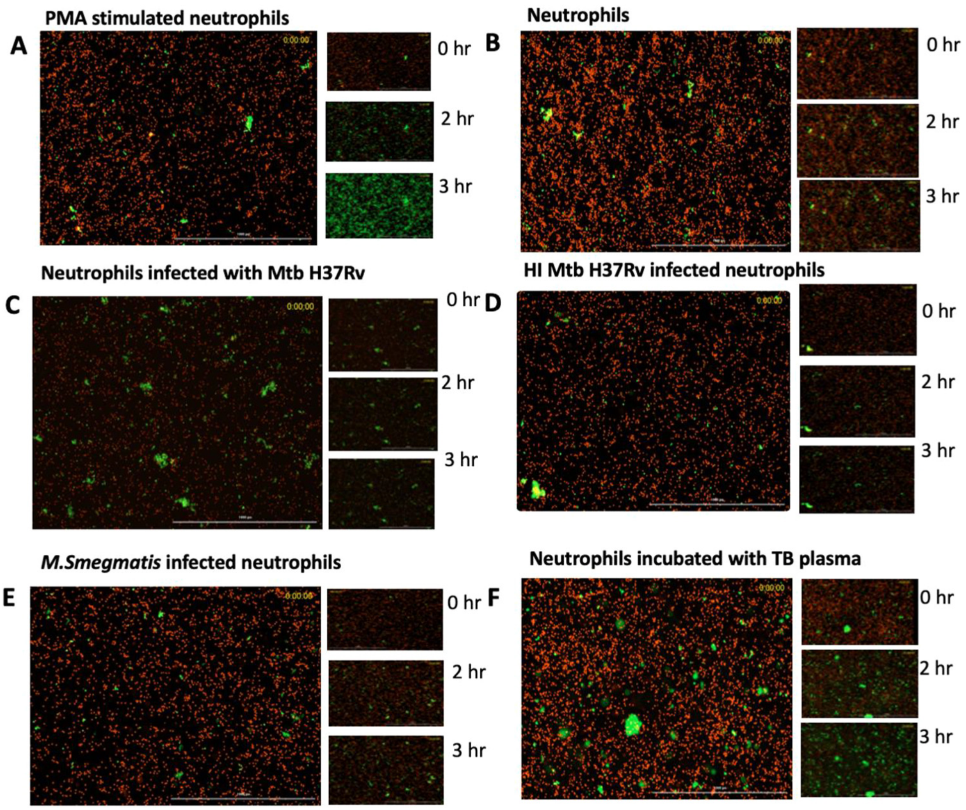

Figure EV4. Pathogenicity and viability of Mtb H37RV impact NETosis.

Neutrophils were stimulated/infected with (**A**) PMA stimulated neutrophils ($n = 3$), (**B**) neutrophils ($n = 3$), (**C**) neutrophils infected with Mtb H37Rv ($n = 3$), (**D**) neutrophils infected with Heat-Inactivated Mtb H37Rv ($n = 3$), (**E**) neutrophils infected with *M. smegmatis* ($n = 3$), and (**F**) Neutrophils incubated with TB plasma ($n = 3$). The indicated scale represents magnification with the Biotek® cytation 5 Gen 5 software at 1000 µm. Green fluorescence at an excitation/emission of 503/526 nm was detected with GFP filter, and Permeable nuclear red at 622/645 nm was detected with Cy5 filter using the Biotek® Cytation 5 cell imaging multi-mode reader. Images were captured every 30 min for 6 h and represent a single section within each well.

© The Author(s)

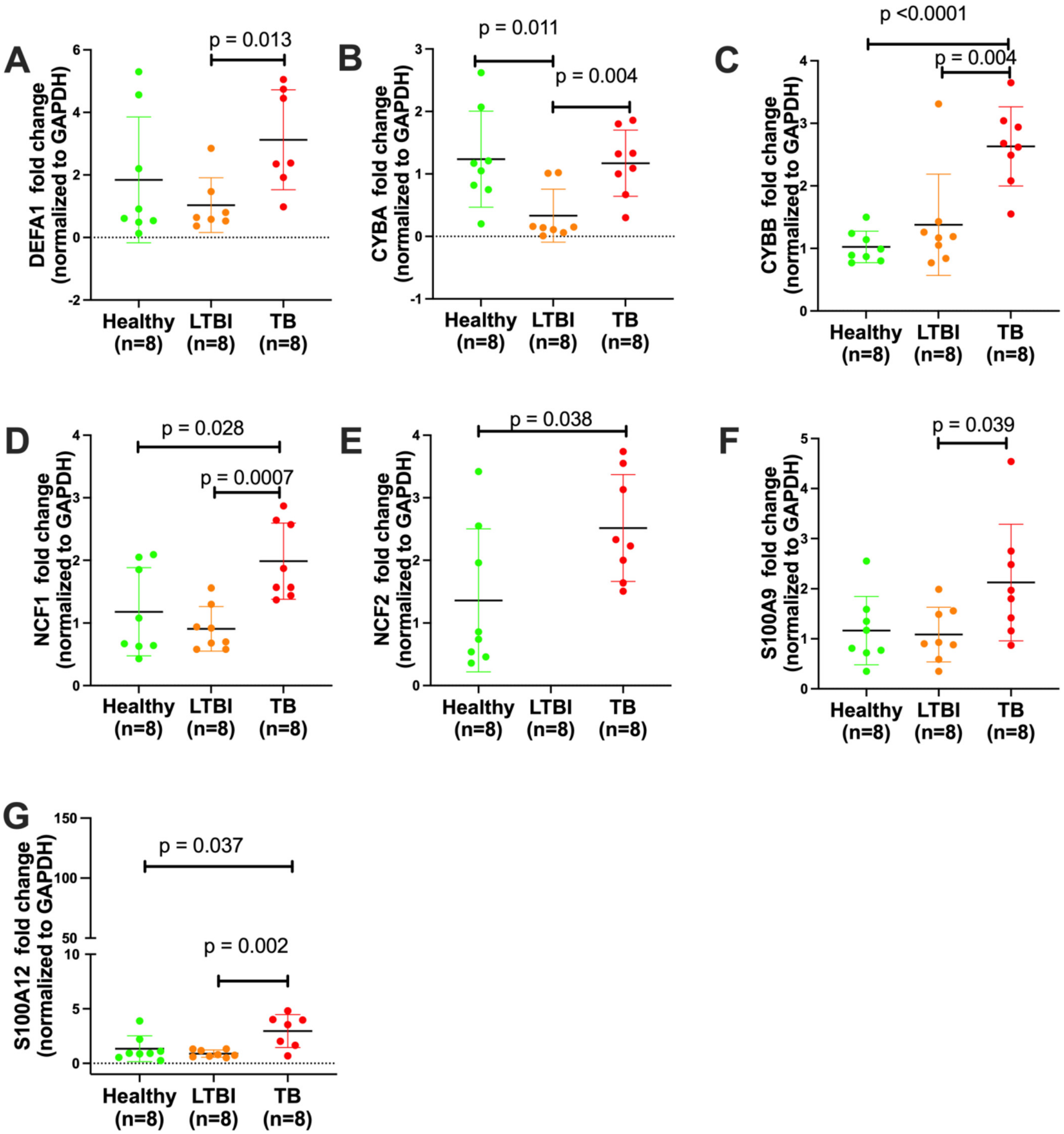

Figure EV5. Gene expression analysis of candidate genes in healthy ($n = 8$), LTBI ($n = 8$), and TB ($n = 8$) participants.

(**A**) DEFA1 was significantly upregulated in the TB arm compared to the LTBI arm ($p = 0.013$), (**B**) CYBA was more upregulated in the healthy arm compared to the LTBI arm ($p = 0.011$) and in the TB arm compared to the LTBI arm ($p = 0.004$), (**C**) CYBB was upregulated in the TB arm compared to the healthy arm ($p < 0.0001$) and LTBI arm ($p = 0.004$), (**D**) NCF1 was upregulated in the TB arm compared to the healthy arm ($p = 0.028$) and the LTBI arm ($p = 0.0007$), (**E**) NCF2 was more upregulated in the TB arm compared to the healthy arm ($p = 0.038$), (**F**) S100A9 was more upregulated in the TB arm compared to the LTBI arm ($p = 0.039$) and (**G**) S100A12 was upregulated in the TB arm compared to the healthy arm ($p = 0.037$) arm and LTBI arm ($p = 0.002$). All gene expression values were normalized to GAPDH using the $2\Delta\Delta^{ct}$ method. Unpaired *t*-tests were used to determine significant ($p < 0.05$) differences between groups.

© The Author(s)
