# Supplementary material for: NETosis associates with human TB lung tissue destruction and disease pathogenesis
Source: EMBO Mol Med. 2026 Jun 2;18(7):2547–72. doi: 10.1038/s44321-026-00435-3 (PMC13365388; doi:10.1038/s44321-026-00435-3)
Supplement: Supplementary file 3 — Peer Review File [file 44321_2026_435_MOESM3_ESM.pdf]

EMBO Molecular Medicine

NETosis associates with human TB lung tissue destruction and disease pathogenesis

Kimone Fisher, Thabo Mpotje, Kievershen Nargan, Denelle Moodley, Kerishka Rajkumar-Bhugeloo, Omolara Baiyegunhi, Threnesan Naidoo, Rajhmun Madansein, Mike Machaba Sathekge, Alasdair Leslie, Gillian Tomlinson, Gabriele Pollara, Mahdad Noursadeghi, Adrie Steyn, Thumbi Ndung'u, and Mohlopheni Marakalala

Corresponding author: Mohlopheni Marakalala (mjmarakalala@sun.ac.za)

Review Timeline:

Submission Date:	24th Mar 25
Editorial Decision:	2nd May 25
Revision Received:	7th Oct 25
Editorial Decision:	21st Oct 25
Revision Received:	15th Apr 26
Accepted:	20th Apr 26

Editor: Zeljko Durdevic

Transaction Report:

(Note: With the exception of the correction of typographical or spelling errors that could be a source of ambiguity, letters and reports are not edited. Depending on transfer agreements, referee reports obtained elsewhere may or may not be included in this compilation. Referee reports are anonymous unless the Referee chooses to sign their reports.)

2nd May 2025

Dear Dr. Marakalala,

Thank you for the submission of your manuscript to EMBO Molecular Medicine. We have now received feedback from the three reviewers who agreed to evaluate your manuscript. As you will see from the reports pasted below, while the referees #1 and #3 are overall supportive raising important concerns, referee #2 recognize the interest of the study but also raises serious concerns particularly regarding the inappropriate disease controls.

After the cross-commenting discussion it became clear that establishing a new cohort, such as individuals with streptococcal pneumonia as suggested by the referee #2, is a substantial undertaking and not necessary for the scope of this study. This point should be addressed by explicitly acknowledging it as a limitation and discuss the potential value of including a broader disease comparator in the future studies to extend the applicability of the findings. The focus of the major revision should be on providing more mechanistic insight and performing additional experiments that further strengthen the main conclusions of the study. If you would like to discuss further the points raised by the referees, I am available to do so via email or video. Let me know if you are interested in this option.

Further consideration of a revision that addresses reviewers' concerns in full will entail a second round of review. EMBO Molecular Medicine encourages a single round of revision only and therefore, acceptance or rejection of the manuscript will depend on the completeness of your responses included in the next, final version of the manuscript. For this reason, and to save you from any frustrations in the end, I would strongly advise against returning an incomplete revision.

We would welcome the submission of a revised version within three months for further consideration. Please let us know if you require longer to complete the revision.

I look forward to receiving your revised manuscript.

Yours sincerely,

Zeljko Durdevic

Zeljko Durdevic
Editor
EMBO Molecular Medicine

*****

When submitting your revised manuscript, please carefully review the instructions that follow below. We perform an initial quality control of all revised manuscripts before re-review; failure to include requested items will delay the evaluation of your revision.

We require:

1) A .docx formatted version of the manuscript text (including legends for main figures, EV figures and tables). Please make sure that the changes are highlighted to be clearly visible.

2) Individual production quality figure files as .eps, .tif, .jpg (one file per figure). For guidance, download the 'Figure Guide PDF': (https://www.embopress.org/page/journal/17574684/authorguide#figureformat).

3) A .docx formatted letter INCLUDING the reviewers' reports and your detailed point-by-point responses to their comments. As part of the EMBO Press transparent editorial process, the point-by-point response is part of the Review Process File (RPF), which will be published alongside your paper.

4) A complete author checklist, which you can download from our author guidelines

(https://www.embopress.org/page/journal/17574684/authorguide#submissionofrevisions). Please insert information in the checklist that is also reflected in the manuscript. The completed author checklist will also be part of the RPF.

5) Please note that all corresponding authors are required to supply an ORCID ID for their name upon submission of a revised manuscript.

6) It is mandatory to include a 'Data Availability' section after the Materials and Methods. Before submitting your revision, primary datasets produced in this study need to be deposited in an appropriate public database, and the accession numbers and database listed under 'Data Availability'. Please remember to provide a reviewer password if the datasets are not yet public (see https://www.embopress.org/page/journal/17574684/authorguide#dataavailability).

In case you have no data that requires deposition in a public database, please state so in this section. Note that the Data Availability Section is restricted to new primary data that are part of this study.

7) For data quantification: please specify the name of the statistical test used to generate error bars and P values, the number (n) of independent experiments (specify technical or biological replicates) underlying each data point and the test used to calculate p-values in each figure legend. The figure legends should contain a basic description of n, P and the test applied. Graphs must include a description of the bars and the error bars (s.d., s.e.m.). See also 'Figure Legend' guidelines: https://www.embopress.org/page/journal/17574684/authorguide#figureformat

8) At EMBO Press we ask authors to provide source data for the main manuscript figures. You will receive a separate email with instructions for providing source data with your revised manuscript, including how to upload and organize the files.

9) Our journal encourages inclusion of *data citations in the reference list* to directly cite datasets that were re-used and obtained from public databases. Data citations in the article text are distinct from normal bibliographical citations and should directly link to the database records from which the data can be accessed. In the main text, data citations are formatted as follows: "Data ref: Smith et al, 2001" or "Data ref: NCBI Sequence Read Archive PRJNA342805, 2017". In the Reference list, data citations must be labeled with "[DATASET]". A data reference must provide the database name, accession number/identifiers and a resolvable link to the landing page from which the data can be accessed at the end of the reference. Further instructions are available at .

10) We replaced Supplementary Information with Expanded View (EV) Figures and Tables that are collapsible/expandable online. A maximum of 5 EV Figures can be typeset. EV Figures should be cited as 'Figure EV1, Figure EV2" etc... in the text and their respective legends should be included in the main text after the legends of regular figures.

- For the figures that you do NOT wish to display as Expanded View figures, they should be bundled together with their legends in a single PDF file called *Appendix*, which should start with a short Table of Content. Appendix figures should be referred to in the main text as: "Appendix Figure S1, Appendix Figure S2" etc.

- Additional Tables/Datasets should be labeled and referred to as Table EV1, Dataset EV1, etc. Legends have to be provided in a separate tab in case of .xls files. Alternatively, the legend can be supplied as a separate text file (README) and zipped together with the Table/Dataset file.

See detailed instructions here:

.

11) The paper explained: EMBO Molecular Medicine articles are accompanied by a summary of the articles to emphasize the major findings in the paper and their medical implications for the non-specialist reader. Please provide a draft summary of your article highlighting

- the medical issue you are addressing,

- the results obtained and

- their clinical impact.

This may be edited to ensure that readers understand the significance and context of the research. Please refer to any of our published articles for an example.

12) Author contributions: You will be asked to provide CRediT (Contributor Role Taxonomy) terms in the submission system. These replace a narrative author contribution section in the manuscript.

13) A Conflict of Interest statement should be provided in the main text.

14) Every published paper now includes a 'Synopsis' to further enhance discoverability. Synopses are displayed on the journal webpage and are freely accessible to all readers. They include a short stand first (maximum of 300 characters, including space) as well as 2-5 one-sentences bullet points that summarizes the paper. Please write the bullet points to summarize the key NEW findings. They should be designed to be complementary to the abstract - i.e. not repeat the same text. We encourage inclusion of key acronyms and quantitative information (maximum of 30 words / bullet point). Please use the passive voice. Please attach these in a separate file or send them by email, we will incorporate them accordingly.

Please also suggest a striking image or visual abstract to illustrate your article as a PNG file 550 px wide x 300-600 px high.

15) Include a Reagents and Tools Table as part of the Methods section, which can be downloaded from our author guidelines (https://www.embopress.org/page/journal/17574684/authorguide#structuredmethods)

EMBO Molecular Medicine has a "scooping protection" policy, whereby similar findings that are published by others during review or revision are not a criterion for rejection. Should you decide to submit a revised version, I do ask that you get in touch after three months if you have not completed it, to update us on the status.

***** Reviewer's comments *****

Referee #1 (Remarks for Author):

Fisher and colleagues present a comprehensive and well analyzed set of data implicating NETosis as driver of lung issue damage in TB granulomas. Their research demonstrates that a neutrophil gene signature correlated with inflammatory disease and might serve as biomarker for active tuberculosis. Inhibition of neutrophil derived enzymes resulted in reduced Mtb induced NETosis which provided a mechanistic link and suggests a potential path for host directed therapy. NETosis associated genes were also upregulated in TB patients compared to individuals with LTBI a human in vivo tuberculin skin test (TST) model further validating their findings and reveling the clinical relevance of these findings. The data are well analyzed and clearly presented. Overall, this is an exciting and timely study, that take advantage of informative human tissue samples and provides new insights into TB pathogenesis.

I have some minor comments that the authors should address.
Please change "TB.....kills approximately 1..5 million people each year" to 1.3 million and update the refence with the latest WHO report.

The human in vivo tuberculin skin test (TST) model should be better explained to make it understandable for a nonspecialist.

There are several wording errors throughout the manuscript and I suggest that a native English speaker edits the manuscript. A few examples that need correction:
Line 58/59: The phenotypes included highly activated, proapoptotic phenotypes and immunoregulatory neutrophil subsets..." the second "phenotypes" should be removed
Line 97: "has" should be "have"
Line 364: "highly enriched in the caseum than cellular region" exchange "than" with "compared to"
Line 441; 'region the NETs enrichment..." should be "region of the NETS"
Line 439: "our proteins" exchange with "the studied proteins" or "the identified proteins"

The discussion is very lengthy, and difficult to read and fails to focus on and highlight the most important discoveries. It also contains unnecessary summaries/repetitions of the results. It should be significantly shortened and streamlined.

Referee #2 (Comments on Novelty/Model System for Author):

As described in note to authors, the use of healthy IGRA+ individuals is not the appropriate control here to determine potential causality of PMNs in active TB. Ideally, disease severity spectrum of ATB or alternative disease controls should be used.

Referee #2 (Remarks for Author):

Neutrophils are crucial components of innate immunity. However, paradoxically, they have been implicated in pathogenesis in both preclinical and clinical studies of tuberculosis. One aspect of neutrophil function implicated in TB pathogenesis is the

formation of extracellular traps, NETosis. Here, Fisher and colleagues explore this idea using human samples derived from healthy individuals (both IGRA- and IGRA+) and patients with a diagnosis of active TB. They leverage proteomic and other tools to explore the correlation between NETosis and active TB. They confirm that human granuloma samples contain neutrophils and neutrophil-derived proteins, particularly in caseum. The major findings are that markers of NETOSis are identified in caseum of human granulomas and that, in keeping with prior work, Mtb addition to ex vivo PMNs induce NETosis and that this can be inhibited with known inhibitors of the pathway. A major strength of this observational study is the use of human samples. However, it is hampered by lack of appropriate disease controls and lack of mechanistic insights that infer causality.

Comments

1. L. 371: "suggests a neutrophil mediated role in the granuloma progression and tissue destruction." It is hard to justify this sentence from an observational study of a small number of lung sections.

2. A major concern about the study design is that the comparator groups for active TB patients are healthy individuals (both IGRA+ and IGRA-). LTBI individuals, by definition, are not sick, and indeed, many will not even be latently infected, but will have cleared infection. Therefore, is it surprising that a disease cohort (active TB, ATB) expresses higher neutrophil activation markers than a health cohort? To really link the assertion as potentially causal, a better disease comparator group, e.g. those with streptococcal pneumonia (where neutrophils are generally considered protective) would be helpful. As such, while the authors demonstrate that ATB is associated with markers of neutrophil activation, this doesn't really shed any light on specific disease pathogenesis. In support of this, in general, markers identified were similar between IGRA- and IGRA+ health controls.

3. On this same note, can the authors determine if the markers of neutrophil activation or NETOsis at least correlate with Mtb burden in ATB individuals - since all the ATB patients were Xpert+, the Ct value of qPCR can serve as a surrogate of bacterial burden. That would advance the study in terms of correlating the identified markers with potential disease severity.

4. Minor comment, but BCG would serve as a better control than M. smegmatis to determine if pathogenic Mtb is needed to activate NETOsis.

Minor comments
1. The abstract is full of jargon/abbreviations (e.g. MPO, CYBB etc), these should be written out on first encounter, and in general, would benefit from clearer writing (e.g. L43 of the abstract needs complete rewriting).

2. Throughout much of the manuscript, there is a confusion between "in vitro", "ex vivo" and "in vivo" - e.g. the assay with human neutrophils from healthy donors should be "ex vivo" or "in vitro", but not "in vivo" etc.

3. L 357: why weren't these neutrophil proteins reported in the previous work? Is that because they weren't expressed at high levels or identified with low confidence? The following sentence suggests that low level expression wasn't the reason.

Referee #3 (Remarks for Author):

This study presents compelling evidence that NETosis-a form of neutrophil-mediated cell death-contributes to tissue damage in tuberculosis (TB). The authors convincingly demonstrate the presence of NETosis-associated proteins in necrotic regions of human TB granulomas, implicating this process in disease pathology. Functional studies further support this link, as genetic inhibition of key NETosis pathways reduced neutrophil death in vitro, pointing to a potential therapeutic strategy. The clinical relevance of these findings is underscored by the observed upregulation of NETosis-related genes in blood samples from individuals with active TB and following tuberculin skin test (TST) challenge. Overall, this is a highly significant study that supports the hypothesis that NETosis is not only a marker but also a driver of TB progression. However, the manuscript would benefit from addressing the minor but important points outlined below.
1. The criteria for classifying granulomas into solid, caseous, and necrotizing caseous types are not clearly described. Please elaborate on the morphological characteristics and any molecular or histological markers used to distinguish these granuloma types or regions.
2. The brown staining in Figure 1 is difficult to interpret because the background coloration is also brown-ish, making it challenging to distinguish signal from background. Using a more contrasting pseudocolor may improve clarity.
3. Please clarify the nature of the black streak visible in Figure 2C. It appears to be an artifact, but an explanation is needed.
4. The intense yellow signal observed in the central region of Caseum 3 in Figure 4B may represent tissue damage or a false positive signal, rather than true MPO staining. Please verify and clarify this in the text.
5. Some figure legends, such as that for Figure 4, are difficult to follow. Consider revising them to provide more detailed and clear descriptions of the experimental conditions, staining, and interpretation.
6. The statistical data supporting Figure 5 are not clearly shown. The text in the manuscript states that inhibitors significantly

reduced NETosis over a 6-hour incubation ($p < 0.05$), but it is unclear which inhibitors, which time points were analyzed, and what statistical tests were applied. Please include these details in the figure and/or legend.

7. Recent studies (PMID: 39637864; 33149141) have also explored related mechanisms. While these do not detract from the novelty of the current work, especially given its use of human samples, the manuscript would be strengthen if the authors discussed in the context of the their findings.

Referee #1 (Remarks for Author):

Fisher and colleagues present a comprehensive and well-analysed set of data implicating NETosis as a driver of lung tissue damage in TB granulomas. Their research demonstrates that a neutrophil gene signature correlated with inflammatory disease and might serve as a biomarker for active tuberculosis. Inhibition of neutrophil-derived enzymes resulted in reduced Mtb-induced NETosis, which provided a mechanistic link and suggests a potential path for host-directed therapy. NETosis-associated genes were also upregulated in TB patients compared to individuals with LTBI, a human in vivo tuberculin skin test (TST) model, further validating their findings and revealing the clinical relevance of these findings. The data are well analysed and clearly presented. Overall, this is an exciting and timely study that takes advantage of informative human tissue samples and provides new insights into TB pathogenesis

Query 1:
I have some minor comments that the authors should address.
Please change "TB.....kills approximately 1..5 million people each year" to 1.3 million and update the reference with the latest WHO report.

Response 1:

We thank the reviewer for pointing this out. The sentence has been edited from 1.5 to 1.3 million and the latest WHO TB global report has been cited.

Query 2:

The human in vivo tuberculin skin test (TST) model should be better explained to make it understandable for a nonspecialist.

Response 2:

We thank the reviewer for the suggestion. The section has been revised and we added the following explanation under the method section describing the

*'Transcriptional profiling of neutrophil related genes in the human TST challenge model'***:** Transcriptional profiling of neutrophil related genes was analyzed using our previously published dataset[9], obtained from a human TST challenge model, in which individuals with LTBI or TB were exposed to Mtb specific antigen, via a tuberculin skin test (TST), and immune response was analyzed at the site of TST challenge. Appropriate citation was also added.

Query 3:

There are several wording errors throughout the manuscript and I suggest that a native English speaker edits the manuscript. A few examples that need correction:

Line 58/59: The phenotypes included highly activated, proapoptotic phenotypes and immunoregulatory neutrophil subsets..." the second "phenotypes" should be removed
Line 97: "has" should be "have"
Line 364: "highly enriched in the caseum than cellular region" exchange "than" with "compared to"
Line 441; 'region the NETs enrichment..." should be "region of the NETS"
Line 439: "our proteins" exchange with "the studied proteins" or "the identified proteins"

Response 3:
We thank the reviewer for pointing out the wording errors. We have edited the manuscript, including corrections of the errors pointed out:

Line 63 (originally Line 58/59): the second phenotypes has been removed

Line 108 (originally 97): “has” has been replaced with “have”

Line 383 (originally 364): “than” has been replaced with “compared to”

Line 435 (originally 441); 'region the NETs enrichment..." has been fixed to "region of the NETs enrichment…"

Line 471 (originally 439): "our proteins" has been changed to "the identified proteins"

Query 4:
The discussion is very lengthy, and difficult to read and fails to focus on and highlight the most important discoveries. It also contains unnecessary summaries/repetitions of the results. It should be significantly shortened and streamlined.

Response 4:

We thank the reviewer for the recommendation. Unnecessary parts of the discussion have been deleted and it has now been streamlined, although some additional parts had to be added to accommodate recommendations made by other reviewers/the editor; including on discussions about study controls and discussion of recently published manuscripts.

Referee #2 (Comments on Novelty/Model System for Author):

As described in note to authors, the use of healthy IGRA+ individuals is not the appropriate control here to determine potential causality of PMNs in active TB. Ideally, disease severity spectrum of ATB or alternative disease controls should be used.

Response:
The LTBI (+IGRA) group was designed not as a control group but rather another group we sought to investigate within our study. Our control group were the healthy (IGRA-) individuals. The reason we also included LTBI in our study was to determine if there were any NETosis markers upregulated upon infection, before the onset of the clinical disease. However, we did not see any NETosis specific markers associated with LTBI group, which had similar response to healthy controls. We appreciate the reviewer's insights that 'LTBI' may also represent individuals that are not 'latently infected' but those that may have cleared infection. We appreciate that an experimental group with a well characterized disease severity spectrum would

give a conclusive association of NETosis with disease severity; and this could be considered for future work in a larger sample cohorts.

Referee #2 (Remarks for Author):

Neutrophils are crucial components of innate immunity. However, paradoxically, they have been implicated in pathogenesis in both preclinical and clinical studies of tuberculosis. One aspect of neutrophil function implicated in TB pathogenesis is the formation of extracellular traps, NETosis. Here, Fisher and colleagues explore this idea using human samples derived from healthy individuals (both IGRA- and IGRA+) and patients with a diagnosis of active TB. They leverage proteomic and other tools to explore the correlation between NETosis and active TB. They confirm that human granuloma samples contain neutrophils and neutrophil-derived proteins, particularly in caseum. The major findings are that markers of NETOSis are identified in caseum of human granulomas and that, in keeping with prior work, Mtb addition to ex vivo PMNs induce NETosis and that this can be inhibited with known inhibitors of the pathway. A major strength of this observational study is the use of human samples.

However, it is hampered by lack of appropriate disease controls and lack of mechanistic insights that infer causality.

Comments

Query 1:

L. 371: "suggests a neutrophil mediated role in the granuloma progression and tissue destruction." It is hard to justify this sentence from an observational study of a small number of lung sections.

Response 1:

We thank the reviewer for the comment. We have changed the above-mentioned sentence to emphasize spatial association rather than potential causality. The sentence now, now Line 390, reads: “Our data reveals association of neutrophil related proteins with TB granuloma caseum.”

Query 2.

A major concern about the study design is that the comparator groups for active TB patients are healthy individuals (both IGRA+ and IGRA-). LTBI individuals, by definition, are not sick, and indeed, many will not even be latently infected, but will have cleared infection. Therefore, is it surprising that a disease cohort (active TB, ATB) expresses higher neutrophil activation markers than a health cohort? To really link the assertion as potentially causal, a better disease comparator group, e.g. those with streptococcal pneumonia (where neutrophils are generally considered protective) would be helpful. As such, while the authors demonstrate that ATB is associated with markers of neutrophil activation, this doesn't really shed any light on specific disease pathogenesis. In support of this, in general, markers identified were similar between IGRA- and IGRA+ health controls.

Response 2:

We appreciate the reviewer’s useful suggestions. We agree that the inclusion of a broader disease comparator would strengthen the link of neutrophil mediated response to TB pathogenesis. As noted by the editor in the decision letter, that after the cross-commenting discussion it became clear that establishing a new cohort, such as individuals with streptococcal pneumonia as suggested, is a substantial undertaking and not necessary for the scope of this study. We have acknowledged this point as a limitation of our study and also discussed the potential value of including a broader disease comparator in the future studies to extend the applicability of the findings. The following sentence has been included in the discussion (Line 642-646):

“A limitation of our study is the lack of a broader non-TB disease comparator group. Including a disease control such as *Streptococcus pneumoniae*, where NETosis has

been characterized (https://pmc.ncbi.nlm.nih.gov/articles/PMC8008068/), would have strengthened our understanding of neutrophil-driven lung damage. For future studies, such broader disease controls should be considered for understanding of neutrophil-driven mechanisms in TB pathogenesis”

Query 3:

On this same note, can the authors determine if the markers of neutrophil activation or NETOsis at least correlate with Mtb burden in ATB individuals - since all the ATB patients were Xpert+, the Ct value of qPCR can serve as a surrogate of bacterial burden. That would advance the study in terms of correlating the identified markers with potential disease severity.

Response 3:

We appreciate the suggestion by the reviewer. Our previously published work has shown correlation of neutrophil count with disease severity, measured through chest X-ray score and bacterial loads. More details and figures are at the bottom of this response. To confirm the association of neutrophil activation/NETosis with potential pathological severity, we have now performed additional experiments staining for MPO, Neutrophil elastase and Citrulline H3 in tissues with ranging pathological damage, including non-necrotic lesions and caseous granulomas with necrotic core, potentially representing different levels of pathological severity. We also included an additional experiment with a non-TB lung control tissue from a participant who underwent surgery due to cancer. New immunofluorescence images, as shown below, were incorporated into the revised manuscript (Figure 4 C, D and EV Figure 3 E,F,G,H) and the results section ‘Neutrophil extracellular traps co-localize with neutrophil elastase in caseous granulomas’ has been updated as follows:

‘…we found that citrulline H3 was abundant in the caseous centre of the granulomas, and consistently colocalized with NE in all the heterogeneous forms of the caseum we sampled (Figure 4B, C and Expanded View Figure 3C, D).

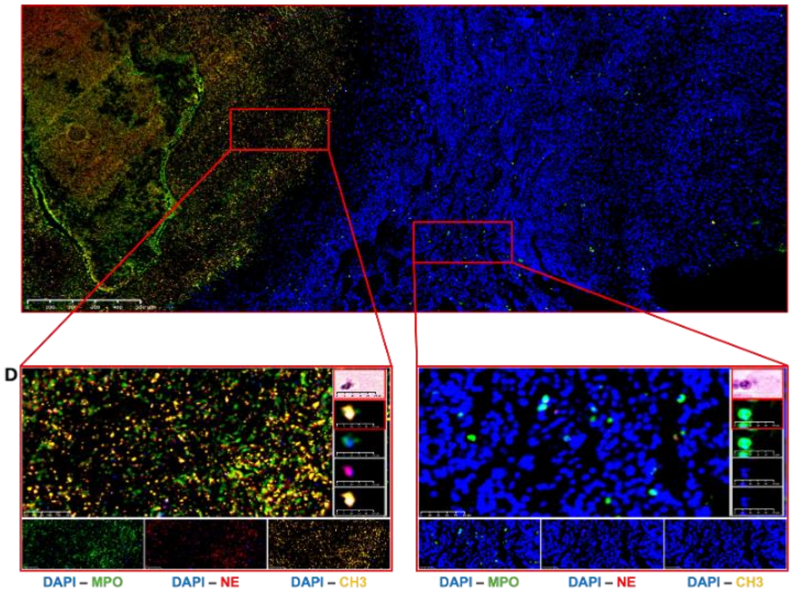

Caseous granuloma

In contrast, this co-localization was absent in non-necrotic granuloma (Expanded View Figure 3E and F). The zoomed in images indicated the striking association of the NE activity alongside NET release in the areas of tissue damage (Figure 4C, D and Expanded View Figure 3D). In contrast, only very little NE was found in the cellular region outside the caseum, with no evidence of colocalization with NETs (Figure 4C, D and Expanded View Figure 3D). *Further cellular-level analysis showed that neutrophils (MPO-positive) within the caseous regions exhibited stronger co-localization of NE and citrulline H3 compared to those in the (Figure 4C, D) outer cellular areas, indicating increased NETosis activity in the caseous regions. Additionally, the co-localization of NE with citrullinated H3 was minimal in control cancer-associated necrotic lesions, suggesting that the heightened NETosis activity may be specifically driven by TB (Expanded View Figure 3G and H)*

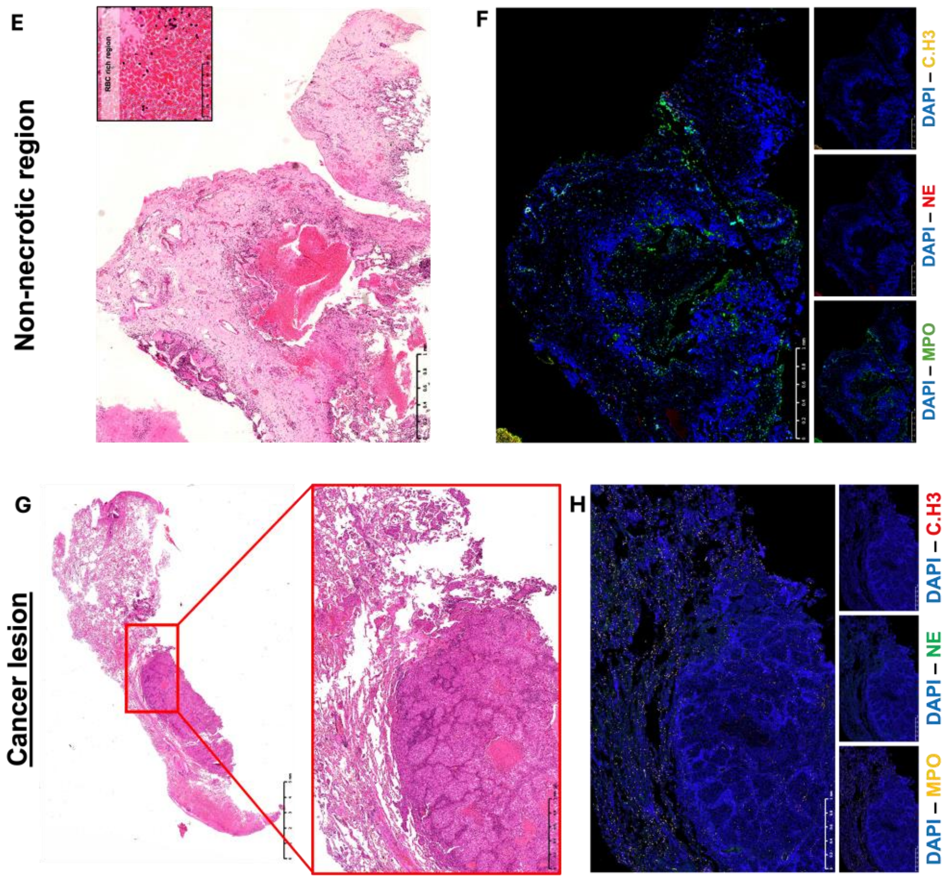

Our previous work (https://pubmed.ncbi.nlm.nih.gov/32983107/) has reported a correlation of neutrophil count with mycobacterial load and Chest X-ray score, suggesting an association with disease severity (as shown in D and E below). Of course, these data cannot be used for the current manuscript, but we sincerely hope the combination of the additional tissue work above, together with the published neutrophil correlation with bacterial load and chest X-ray, provide evidence of potential association of with disease severity.

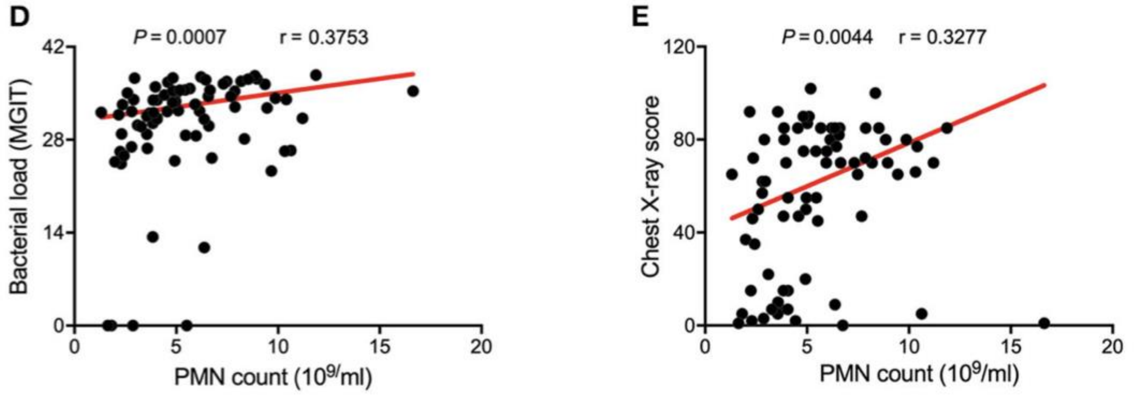

https://pubmed.ncbi.nlm.nih.gov/32983107/

Query 4: Minor comment, but BCG would serve as a better control than M. smegmatis to determine if pathogenic Mtb is needed to activate NETOsis.

Response 4:

We appreciate the reviewer's suggestion of BCG as a control. *M. smegmatis* shares a high degree of genetic homology with *M. tuberculosis*. In addition, *M. smegmatis* has been widely used as a non-pathogenic model strain in mycobacteriology. While BCG would be a good control as the reviewer suggested, we believe *M. smegmatis*, which is not virulent or pathogenic, also make a good control to determine whether pathogenicity is an important factor in NETosis induction.

Minor comments

1. The abstract is full of jargon/abbreviations (e.g. MPO, CYBB etc), these should be written out on first encounter, and in general, would benefit from clearer writing (e.g. L43 of the abstract needs complete rewriting).

2. Throughout much of the manuscript, there is a confusion between "in vitro", "ex vivo" and "in vivo" - e.g. the assay with human neutrophils from healthy donors should be "ex vivo" or "in vitro", but not "in vivo" etc.

3. L 357: why weren't these neutrophil proteins reported in the previous work? Is that because they weren't expressed at high levels or identified with low confidence? The following sentence suggests that low level expression wasn't the reason.

Response to minor comments:

1. We thank the reviewer for the recommendations. We have fully written out the abbreviations MPO, CYBB and NCF1. " Using immunohistochemistry (IHC) we validated the abundance of neutrophil associated proteins, including myeloperoxidase (MPO), cytochrome b-245 beta chain (CYBB) and neutrophil

cytosolic factor 1(NCF1), as well as NETosis markers, neutrophil elastase (NE) and citrullinated H3, in necrotizing caseum of human TB granulomas."

2. Corrections have been made regarding the use of in-vitro/ex-vivo instead of in-vivo, including the following:

-Line 120-121 (originally 114) "Targeting specific neutrophil genes resulted in the reduction of NETosis in a neutrophil **in vitro** model of Mtb infection."

Line 572 (originally) 551: **In vivo** was removed form the following sentence: "NETosis associated genes are upregulated in TB compared to LTBI in a human standardized antigen challenge model."

3. Thanks for the query. These proteins were not reported because they were not the focus of the previous study. The previous work, Marakalala et al, 2016, reported on over 3000 proteins in different regions of lung granulomas, with proteins in the arachidonic pathway utilized for validation by staining in situ. The current work is based on further analysis of the proteomics dataset, but with a focus and detailed characterization on neutrophil related proteins (which were not characterized in the previous work) and mechanisms through which they associate with the TB granuloma caseum.

Referee #3 (Remarks for Author):

This study presents compelling evidence that NETosis-a form of neutrophil-mediated cell death-contributes to tissue damage in tuberculosis (TB). The authors convincingly demonstrate the presence of NETosis-associated proteins in necrotic regions of human TB granulomas, implicating this process in disease pathology. Functional studies further support this link, as genetic inhibition of key NETosis pathways reduced neutrophil death in vitro, pointing to a potential therapeutic strategy. The clinical relevance of these findings is underscored by the observed upregulation of NETosis-related genes in blood samples from individuals with active TB and following tuberculin skin test (TST) challenge. Overall, this is a highly significant study that supports the hypothesis that NETosis is not only a marker but also a driver of TB progression. However, the manuscript would benefit from addressing the minor but important points outlined below.

Query 1

The criteria for classifying granulomas into solid, caseous, and necrotizing caseous types are not clearly described. Please elaborate on the morphological characteristics and any molecular or histological markers used to distinguish these granuloma types or regions.

Response 1

The following paragraph has been added to classify the various granulomas emphasized in this study; Line 72-78 “Although there is a spectrum of granuloma diversity, some of the common types of granulomas include: solid, caseous and cavitary granulomas. Solid granulomas are often well-organised and non-necrotic cellular structures. Caseous granulomas mainly have necrotic centres that are acellular often surrounded by a cellular periphery. Early caseous granulomas may also be characterized by presence of karyorrhectic debri at the centres. Some necrotizing caseous granulomas, exhibiting extensive central necrosis, may be characterized by liquefaction, frequently accompanied by cavitation”

We have also added the following statement in figure legend 1(Line 701 – 703) to describe the granuloma types used: ‘…solid granulomas with compact cellular clusters (i), early caseous granulomas with cellular debris at the centre (ii), and necrotising caseous granulomas displaying central necrosis (iii)’

Query 2

The brown staining in Figure 1 is difficult to interpret because the background coloration is also brown-ish, making it challenging to distinguish signal from background. Using a more contrasting pseudocolor may improve clarity.

Response 2

We appreciate the reviewer’s comments. To help clarify any discrepancies regarding morphological characteristics that may be difficult to differentiate by IHC, we have performed additional experiments, with immunofluorescent staining of some proteins in Figure 1, including NE and MPO. Both MPO and NE increase with caseation,

which validates findings using the brown staining. The new fluorescent staining data has been added to EV Figure 1 (panel E) (also shown below).

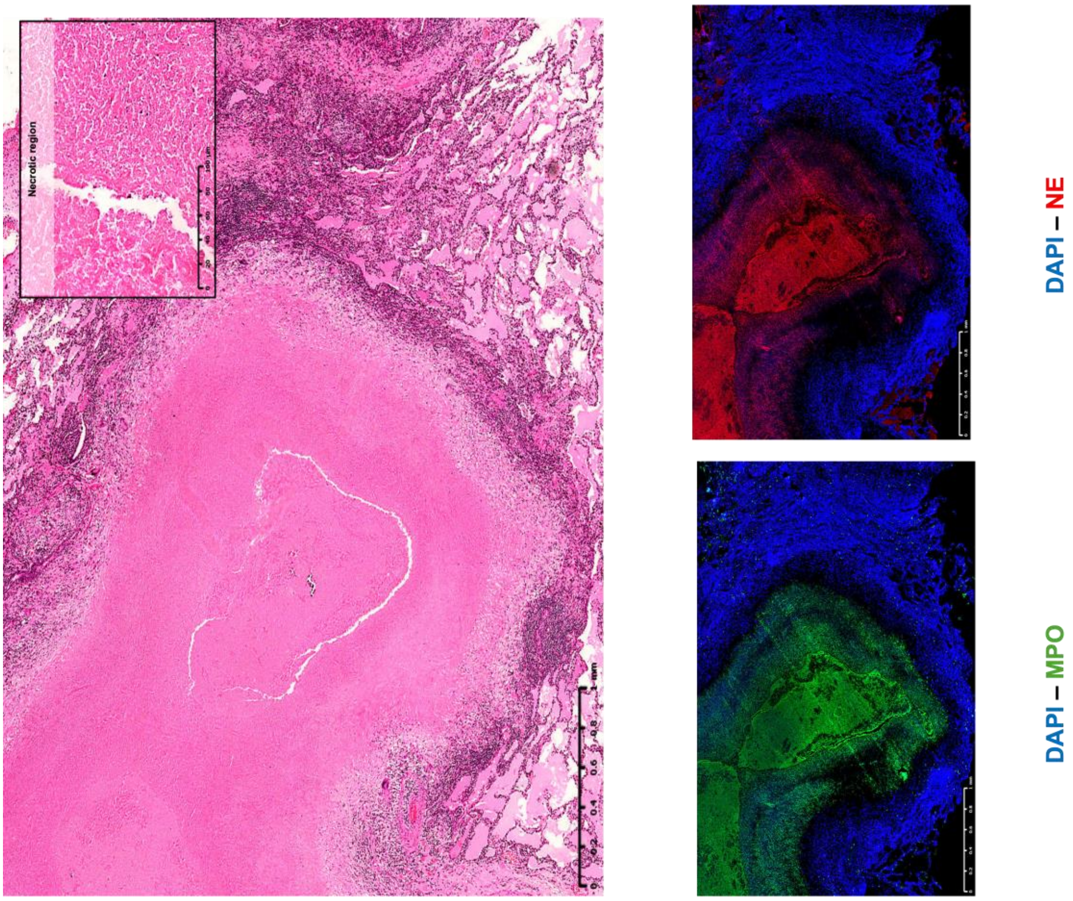

Query 3

Please clarify the nature of the black streak visible in Figure 2C. It appears to be an artifact, but an explanation is needed.

Response 3:

We thank the reviewer for drawing this to our attention. The black streak visible in figure 2c was indeed an artifact of H&E staining which occurred during preparation of the tissue. We have replaced the image with a new 'clean' image of immunofluorescent MPO staining (shown below and in Fig. 2c), showing MPO localization in the centre of the caseum, as initially shown by the old image.

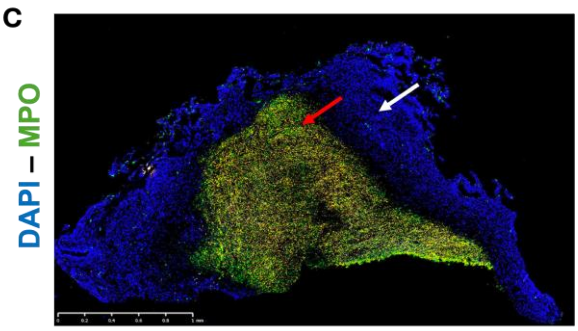

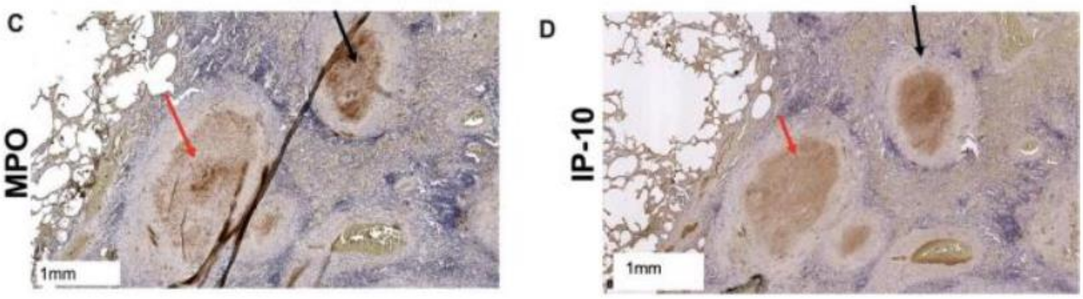

Query 4

The intense yellow signal observed in the central region of Caseum 3 in Figure 4B may represent tissue damage or a false positive signal, rather than true MPO staining. Please verify and clarify this in the text.

Response 4:

We thank the reviewer for drawing our attention to the need for clarification regarding the mentioned image. We agree the yellow signal could represnt an artefact due to tissue damage. We have performed new staining of more similar granulomas. The new data, shown below, has no false positive signal, and has been added to the main figure, Figure 4C, D.

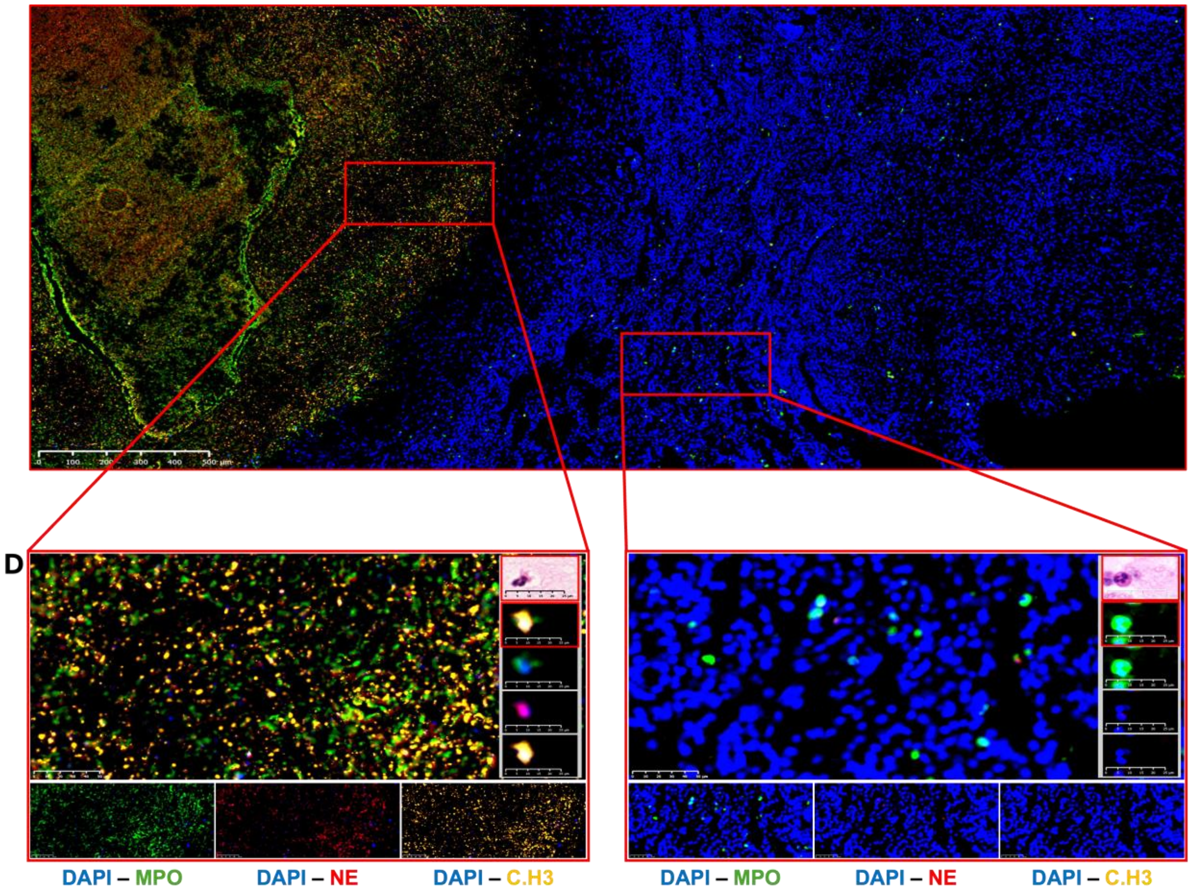

Query 5

Some figure legends, such as that for Figure 4, are difficult to follow. Consider revising them to provide more detailed and clear descriptions of the experimental conditions, staining, and interpretation.

Response 5

Figure 4 legend has been updated to as follows:

Figure 4. NE colocalizes with NETosis in multiple caseous granulomas. H&E staining of human TB-diseased lung illustrating various caseous granulomas, caseum 1, containing karyorrhectic debris at the centre, and caseum 2 and 3, both containing acellular necrotic core. (A). Immunofluorescence staining showing citrulline H3 (green), NE (Red) and MPO (yellow) in various caseous granulomas (B). Illustration of the spatial localization of citrullinated H3 (yellow), MPO (green) and NE (Red) within the necrotic centre of a caseous granuloma (C). Zoomed in images showing IF colocalization of NE and citrulline H3 in the caseum and no

colocalization in the cellular region of the same granuloma (Scale bars represent 25, 50, 500μm, 2.5mm and 5mm.

Query 6

The statistical data supporting Figure 5 are not clearly shown. The text in the manuscript states that inhibitors significantly reduced NETosis over a 6-hour incubation ($p < 0.05$), but it is unclear which inhibitors, which time points were analyzed, and what statistical tests were applied. Please include these details in the figure and/or legend.

Response 6:

More details have been added to Figure 5 legend, which is now updated as follows:

Figure 5: Interception of NE, MPO and NOX2 reduces NETosis induced by Mtb H37Rv infection of healthy neutrophils. Neutrophils were treated with specific pharmaceutical inhibitors at timepoint 0 to determine whether NETosis induced by Mtb infection could potentially be reduced by HDTs. In each condition, (A) Mtb H37Rv-infected neutrophils treated with neutrophil elastase inhibitor Sivelestat 1mM (blue square), (B) NOX2/CYBB inhibitor GSK2795039 at 200 mM (red square) and (C) MPO inhibitor Verdiperstat at 10 mM (purple square) significantly reduced NETosis at the 6 hour mark, compared to the MTB H37Rv infected neutrophil groups ($p<0.05$). The presence of NETosis was lower compared to those neutrophils that were not treated, from time point 0 -6 hrs in each inhibitor condition. Kinetic imaging was performed for a duration of 6 hours, at 30 min intervals. Images were captured at a 1000 mm scale and represent a single section within each well. Repeated Measures Anova were used to compare each time point and each treatment condition and control, significant results were defined as those with $p < 0.05$.

Query 7

Recent studies (PMID: 39637864; 33149141) have also explored related mechanisms. While these do not detract from the novelty of the current work, especially given its use of human samples, the manuscript would be strengthen if the authors discussed in the context of the their findings.

Response 7:

We appreciate the reviewer's feedback regarding more recent publications.
We have added the paragraph below in our discussion (Line 623-631), citing 39637864, Chowdhury et al., (2024) and 33149141, Moreira-Texeira et al (2020):

A recent report by Moreira-Texeira demonstrated that increased type I IFN signalling induces NETosis, promoting mycobacterial growth in the absence of GM-CSF. The type I IFN-induced NETosis was also associated with disease severity in TB-susceptible C3HeB/FeJ mice (Moreira-Texeira et al, 2020). This is consistent with a recent work by Chowdhury et al, which demonstrated that Mtb induces a type I IFN and PAD4-dependent NET release in mouse neutrophils, resulting in bacterial replication (Chowdhury et al, 2024). Interestingly, the NET release was associated with necrotic regions in granulomas obtained from non-human primates (Chowdhury et al, 2024), which is in agreement with our observation of NETosis localization with the caseum in human TB granulomas.

21st Oct 2025

Dear Dr. Marakalala,

Thank you for the submission of your revised manuscript to EMBO Molecular Medicine. I am pleased to inform you that we will be able to accept your manuscript pending the following final amendments:

1) Please address the remaining concern of the referee #2 by reanalyzing the data as suggested.
2) Figures: We note that some images/panels might be reused e.g. Figure 1 B-F and Figure 3A, B & G and within Figure 3 B, C, E and G. Please cite in the respective figure legend every reused image/panel.
3) Author checklist: Please complete the form by entering information about Author, Journal and Manuscript Number at the top of the page.
4) In the main manuscript file, please do the following:
- Please address all comments suggested by our data editors listed below:
o Figure legends:
1. Please note that the exact p values are not provided in the legends of figures 2A, 7B-D, F.
2. Please indicate the statistical test used for data analysis in the legends of figures 7A-J.
3. Please note that information related to n is missing in the legends of figures 2A, 3D, H; 5A-C; 7A-J.
4. Please note that the error bars are not defined in the legends of figures 2A, 3D, H; 5A-C.
- Add up to 5 keywords.
- Please correct the order and headings of the manuscript sections to: Abstract, Introduction, Results, Discussion, Methods, Acknowledgements, Disclosure and competing interests statement, References, Figure legends, Tables and their legends, Expanded View Figure legends.
- In Methods, provide the statement that informed consent was obtained from all human subjects and that the experiments conformed to the principles set out in the WMA Declaration of Helsinki and the Department of Health and Human Services Belmont Report.
- In Methods, add the following paragraph:

Graphics:
(some of the... OR Figure #... OR synopsis) Graphics were created with BioRender.com.
- Rename "Conflict of interest" to "Disclosure and competing interests statement". We updated our journal's competing interests policy in January 2022 and request authors to consider both actual and perceived competing interests. Please review the policy https://www.embopress.org/competing-interests and update your competing interests if necessary.
- Author contributions: Please remove it from the manuscript and specify author contributions in our submission system. CRediT has replaced the traditional author contributions section because it offers a systematic machine-readable author contributions format that allows for more effective research assessment. You are encouraged to use the free text boxes beneath each contributing author's name to add specific details on the author's contribution. More information is available in our guide to authors:
https://www.embopress.org/page/journal/17574684/authorguide#authorshipguidelines
- Indicate in legends exact n and exact p values, not a range, along with the statistical test used. To keep the figures "clear" some authors found providing an Appendix table Sx with all exact p-values preferable. You are welcome to do this if you want to.
- Please rename Expanded View Figure 1 etc. to Figure EV1 etc. and update their callouts in the manuscript. EV figure legend should be placed under the heading Expanded View Figure Legends.
- Correct the reference citation in the text and reference list. In the text a reference should be cited by author and year of publication. Include a space between a word and the opening parenthesis of the reference that follows. In the reference list, citations should be listed in alphabetical order. Where there are more than 10 authors on a paper, 10 will be listed, followed by "et al.". Also, please remove DOIs. Please check "Author Guidelines" for more information.
https://www.embopress.org/page/journal/17574684/authorguide#referencesformat
5) Tables: Please rename the expanded view tables to "Table EV1" and "Table EV2", add legends at the top of each page and update their callouts in the main text and in the Reagent Table.
6) Funding:
- Please merge it with Acknowledgments.
- Please make sure that information about all sources of funding are complete in both our submission system and in the manuscript. Currently, South African Medical690 Research Council (SAMRC) funding is missing in our submission system. Please correct.
7) The Paper Explained: Please add it to the main manuscript file.
8) Synopsis:
- Synopsis image: Please submit the image as a separate 550 px-wide x 300-600 pixels high high-resolution jpeg file. Also, remove the BioRender credit from the file.
- Please check your synopsis text and image before submission with your revised manuscript. Please be aware that in the proof stage minor corrections only are allowed (e.g., typos).
9) As part of the EMBO Publications transparent editorial process initiative (see our Editorial at

http://embomolmed.embopress.org/content/2/9/329), EMBO Molecular Medicine will publish online a Review Process File (RPF) to accompany accepted manuscripts. This file will be published in conjunction with your paper and will include the anonymous referee reports, your point-by-point response and all pertinent correspondence relating to the manuscript. Let us know whether you agree with the publication of the RPF and as here, if you want to remove or not any figures from it prior to publication. Please note that the Authors checklist will be published at the end of the RPF.
10) Please provide a point-by-point letter INCLUDING my comments as well as the reviewer's reports and your detailed responses (as Word file).

I look forward to reading a new revised version of your manuscript as soon as possible.

Yours sincerely,

Zeljko Durdevic

Zeljko Durdevic
Senior Editor
EMBO Molecular Medicine

*** Instructions to submit your revised manuscript ***

*** PLEASE NOTE *** As part of the EMBO Publications transparent editorial process initiative (see our Editorial at https://www.embopress.org/doi/pdf/10.1002/emmm.201000094), EMBO Molecular Medicine will publish online a Review Process File to accompany accepted manuscripts.

In the event of acceptance, this file will be published in conjunction with your paper and will include the anonymous referee reports, your point-by-point response and all pertinent correspondence relating to the manuscript. If you do NOT want this file to be published, please inform the editorial office at contact@embomolmed.org.

When submitting your revised manuscript, please include:

1) a .docx formatted version of the manuscript text (including Figure legends and tables)

2) Separate figure files*

3) supplemental information as Expanded View and/or Appendix. Please carefully check the authors guidelines for formatting Expanded view and Appendix figures and tables at
https://www.embopress.org/page/journal/17574684/authorguide#expandedview

4) a letter INCLUDING the reviewer's reports and your detailed responses to their comments (as Word file).

5) The paper explained: EMBO Molecular Medicine articles are accompanied by a summary of the articles to emphasize the major findings in the paper and their medical implications for the non-specialist reader. Please provide a draft summary of your article highlighting
- the medical issue you are addressing,
- the results obtained and
- their clinical impact.
This may be edited to ensure that readers understand the significance and context of the research.
Please refer to any of our published articles for an example.

6) Author contributions: the contribution of every author must be detailed in a separate section.

7) EMBO Molecular Medicine now requires a complete author checklist (https://www.embopress.org/page/journal/17574684/authorguide) to be submitted with all revised manuscripts. Please use the

checklist as guideline for the sort of information we need WITHIN the manuscript. The checklist should only be filled with page numbers were the information can be found. This is particularly important for animal reporting, antibody dilutions (missing) and exact values and n that should be indicted instead of a range.

8) Every published paper now includes a 'Synopsis' to further enhance discoverability. Synopses are displayed on the journal webpage and are freely accessible to all readers. They include a short stand first (maximum of 300 characters, including space) as well as 2-5 one sentence bullet points that summarise the paper. Please write the bullet points to summarise the key NEW findings. They should be designed to be complementary to the abstract - i.e. not repeat the same text. We encourage inclusion of key acronyms and quantitative information (maximum of 30 words / bullet point). Please use the passive voice. Please attach these in a separate file or send them by email, we will incorporate them accordingly.

You are also welcome to suggest a striking image or visual abstract to illustrate your article. If you do please provide a jpeg file 550 px-wide x 300-600px high.

9) A Conflict of Interest statement should be provided in the main text

10) Please note that we now mandate that all corresponding authors list an ORCID digital identifier. This takes <90 seconds to complete. We encourage all authors to supply an ORCID identifier, which will be linked to their name for unambiguous name identification.

Currently, our records indicate that the ORCID for your account is 0000-0001-7476-1652.

Please click the link below to modify this ORCID:
Link Not Available

11) Include a Reagents and Tools Table as part of the Methods section, which can be downloaded from our author guidelines (https://www.embopress.org/page/journal/17574684/authorguide#structuredmethods)

*Additional important information regarding Figures

Each figure should be given in a separate file and should have the following resolution:
Graphs 800-1,200 DPI
Photos 400-800 DPI
Colour (only CMYK) 300-400 DPI"

Figures are not edited by the production team. All lettering should be the same size and style; figure panels should be indicated by capital letters (A, B, C etc). Gridlines are not allowed except for log plots. Figures should be numbered in the order of their appearance in the text with Arabic numerals. Each Figure must have a separate legend and a caption is needed for each panel.

*Additional important information regarding figures and illustrations can be found at https://bit.ly/EMBOPressFigurePreparationGuideline. See also figure legend preparation guidelines: https://www.embopress.org/page/journal/17574684/authorguide#figureformat

***** Reviewer's comments *****

Referee #2 (Remarks for Author):

The revised manuscript by Fisher and colleagues is much improved.

I still think a relatively trivial additional analysis should be performed, which would considerably strengthen the causal claims, central to this paper. For the n=30 subjects with ATB that were diagnosed with GeneXpert, the Ct value, which is a good proxy for bacterial burden and a reasonable (albeit imperfect) proxy for disease severity, would allow the markers of NETosis analyzed from those samples to be plotted to see if there is a positive correlation. This can be done e.g. for serum MPO (Fig. 2A) and the markers measured in Fig. 6. This would not require any additional experiments, but just a re-analysis of the existing data. Since Ct values are always provided with Xpert if there is a positive diagnosis, the additional information needed for the anlaysis should be available.

Referee #2 (Remarks for Author):

The revised manuscript by Fisher and colleagues is much improved.

I still think a relatively trivial additional analysis should be performed, which would considerably strengthen the causal claims, central to this paper. For the n=30 subjects with ATB that were diagnosed with GeneXpert, the Ct value, which is a good proxy for bacterial burden and a reasonable (albeit imperfect) proxy for disease severity, would allow the markers of NETosis analyzed from those samples to be plotted to see if there is a positive correlation. This can be done e.g. for serum MPO (Fig. 2A) and the markers measured in Fig. 6. This would not require any additional experiments, but just a re-analysis of the existing data. Since Ct values are always provided with Xpert if there is a positive diagnosis, the additional information needed for the analysis should be available.

Authors' response:

We appreciate the reviewer’s comment. Despite exhaustive efforts, Ct values raw data were inaccessible to us. Our study sites only provided a GeneXpert qualitative result, as detected or not detected. We appreciate that the Ct value, as proxy for colony forming units, would have enabled us to perform a correlative link of NETosis markers to disease severity (in the context of bacterial load).

However, we addressed this gap by contextualising our findings in relation to two recent experimental models. Moreira-Teixeira et al. (2020) demonstrated that Type I IFN-induced NETosis actively promotes mycobacterial growth in C3HEb/FeJ mice. And Chowdhury et al. (2024), reported that PAD4-dependent NET release leads to increased bacterial replication. These mechanistic insights from mouse models provide a scientific basis for our findings that the elevated NETosis markers observed in our patient cohort likely signify a disease severity. This is in addition to the data that we included in the earlier revision demonstrating association of neutrophil activation/NETosis with pathological severity, which we demonstrated by staining for MPO, Neutrophil elastase and Citrulline H3 in human tissues with ranging pathological damage, including non-necrotic lesions and caseous granulomas with necrotic core, potentially representing different levels of pathological severity. We have cited and discussed the above two reports.

20th Apr 2026

Dear Dr. Marakalala,

We are pleased to inform you that your manuscript is accepted for publication and is now being sent to our publisher to be included in the next available issue of EMBO Molecular Medicine.

Your manuscript will be processed for publication by EMBO Press. It will be copy edited and you will receive page proofs prior to publication. Please note that you will be contacted by Springer Nature Author Services to complete licensing and payment information.

You may qualify for financial assistance for your publication charges - either via a Springer Nature fully open access agreement or an EMBO initiative. Check your eligibility: https://link.springer.com/journal/44321/how-to-publish-with-us

Should you be planning a Press Release on your article, please get in contact with embo_production@springernature.com as early as possible in order to coordinate publication and release dates.

If you have any questions, please do not hesitate to contact the Editorial Office. Thank you for your contribution to EMBO Molecular Medicine.

Yours sincerely,
Zeljko Durdevic

Zeljko Durdevic
Senior Editor
EMBO Molecular Medicine

>>> Please note that it is EMBO Molecular Medicine policy for the transcript of the editorial process (containing referee reports and your response letter) to be published as an online supplement to each paper. If you do NOT want this, you will need to inform the Editorial Office via email immediately. More information is available here: https://link.springer.com/partners/embo-press/editorial-policies#Peer%20review

EMBO Press Author Checklist

Corresponding Author Name: Mohlopheni J Marakalala	
Journal Submitted to: EMBO Molecular Medicine	
Manuscript Number: EMM-2025-21650	

USEFUL LINKS FOR COMPLETING THIS FORM
The EMBO Journal - Author Guidelines
EMBO Reports - Author Guidelines
Molecular Systems Biology - Author Guidelines
EMBO Molecular Medicine - Author Guidelines

Reporting Checklist for Life Science Articles (updated January
This checklist is adapted from Materials Design Analysis Reporting (MDAR) Checklist for Authors. MDAR establishes a minimum set of requirements in transparent reporting in the life sciences (see Statement of Task: 10.31222/osf.io/9sm4x). Please follow the journal's guidelines in preparing your
Please note that a copy of this checklist will be published alongside your article.

Abridged guidelines for figures
1. Data
The data shown in figures should satisfy the following conditions:
- the data were obtained and processed according to the field's best practice and are presented to reflect the results of the experiments in an accurate and unbiased manner.
- ideally, figure panels should include only measurements that are directly comparable to each other and obtained with the same assay.
- plots include clearly labeled error bars for independent experiments and sample sizes. Unless justified, error bars should not be shown for technical
- if n<5, the individual data points from each experiment should be plotted. Any statistical test employed should be justified.
- Source Data should be included to report the data underlying figures according to the guidelines set out in the authorship guidelines on Data

2. Captions
Each figure caption should contain the following information, for each panel where they are relevant:
- a specification of the experimental system investigated (eg cell line, species name).
- the assay(s) and method(s) used to carry out the reported observations and measurements.
- an explicit mention of the biological and chemical entity(ies) that are being measured.
- an explicit mention of the biological and chemical entity(ies) that are altered/varied/perturbed in a controlled manner.
- the exact sample size (n) for each experimental group/condition, given as a number, not a range;
- a description of the sample collection allowing the reader to understand whether the samples represent technical or biological replicates (including how many animals, litters, cultures, etc.).
- a statement of how many times the experiment shown was independently replicated in the laboratory.
- definitions of statistical methods and measures:
  - common tests, such as t-test (please specify whether paired vs. unpaired), simple χ2 tests, Wilcoxon and Mann-Whitney tests, can be unambiguously identified by name only, but more complex techniques should be described in the methods section;
  - are tests one-sided or two-sided?
  - are there adjustments for multiple comparisons?
  - exact statistical test results, e.g., P values = x but not P values < x;
  - definition of 'center values' as median or average;
  - definition of error bars as s.d. or s.e.m.

Please complete ALL of the questions below.
Select "Not Applicable" only when the requested information is not relevant for your study.

Materials

Newly Created Materials	Information included in the manuscript?	In which section is the information available? (Reagents and Tools Table, Materials and Methods, Figures, Data Availability Section)
New materials and reagents need to be available; do any restrictions apply?	Not Applicable	All reagents used are in the Materials and Methods

Antibodies	Information included in the manuscript?	In which section is the information available? (Reagents and Tools Table, Materials and Methods, Figures, Data Availability Section)
For **antibodies** provide the following information:<br>- Commercial antibodies: RRID (if possible) or supplier name, catalogue number and or/clone number<br>- Non-commercial: RRID or citation	Yes	Information on antibodies used is provided in the materials and methods

DNA and RNA sequences	Information included in the manuscript?	In which section is the information available? (Reagents and Tools Table, Materials and Methods, Figures, Data Availability Section)
**Short novel DNA or RNA including primers, probes**: provide the sequences.	Yes	Supplementary Table 2

Cell materials	Information included in the manuscript?	In which section is the information available? (Reagents and Tools Table, Materials and Methods, Figures, Data Availability Section)
**Cell lines:** Provide species information, strain. Provide accession number in repository **OR** supplier name, catalog number, clone number, and/**OR** RRID.	Not Applicable	
**Primary cultures:** Provide species, strain, sex of origin, genetic modification status.	Not Applicable	
Report if the cell lines were recently **authenticated** (e.g., by STR profiling) and tested for mycoplasma contamination.	Not Applicable	

Experimental animals	Information included in the manuscript?	In which section is the information available? (Reagents and Tools Table, Materials and Methods, Figures, Data Availability Section)
**Laboratory animals or Model organisms:** Provide species, strain, sex, age, genetic modification status. Provide accession number in repository **OR** supplier name, catalog number, clone number, **OR** RRID.	Not Applicable	
**Animal observed in or captured from the field:** Provide species, sex, and age where possible.	Not Applicable	
Please detail **housing and husbandry conditions**.	Not Applicable	

Plants and microbes	Information included in the manuscript?	In which section is the information available? (Reagents and Tools Table, Materials and Methods, Figures, Data Availability Section)
**Plants:** provide species and strain, ecotype and cultivar where relevant, unique accession number if available, and source (including location for collected wild specimens).	Not Applicable	
**Microbes:** provide species and strain, unique accession number if available, and source.	Yes	Mycobacterium tuberculosis H37Rv was used. Information is in the materials and methods section

Human research participants	Information included in the manuscript?	In which section is the information available? (Reagents and Tools Table, Materials and Methods, Figures, Data Availability Section)
If collected and within the bounds of privacy constraints report on age, sex and gender or ethnicity for all study participants.	Yes	Materials and methods

Core facilities	Information included in the manuscript?	In which section is the information available? (Reagents and Tools Table, Materials and Methods, Figures, Data Availability Section)
If your work benefited from core facilities, was their service mentioned in the acknowledgments section?	Yes	Materials and methods

Design

Study protocol	Information included in the manuscript?	In which section is the information available? (Reagents and Tools Table, Materials and Methods, Figures, Data Availability Section)
If study protocol has been **pre-registered, provide DOI in the manuscript**. For clinical trials, provide the trial registration number **OR** cite DOI.	Not Applicable	
Report the **clinical trial registration number** (at ClinicalTrials.gov or equivalent), where applicable.	Not Applicable	

Laboratory protocol	Information included in the manuscript?	In which section is the information available? (Reagents and Tools Table, Materials and Methods, Figures, Data Availability Section)
Provide DOI OR other citation details if **external detailed step-by-step protocols** are available.	Yes	doi: 10.12688/gatesopenres.13472.2

Experimental study design and statistics	Information included in the manuscript?	In which section is the information available? (Reagents and Tools Table, Materials and Methods, Figures, Data Availability Section)
Include a statement about **sample size** estimate even if no statistical methods were used.	Yes	Materials and methods
Were any steps taken to minimize the effects of subjective bias when allocating animals/samples to treatment (e.g. **randomization procedure**)? If yes, have they been described?	Not Applicable	
Include a statement about **blinding** even if no blinding was done.	Not Applicable	
Describe **inclusion/exclusion criteria** if samples or animals were excluded from the analysis. Were the criteria pre-established?<br>If sample or data points were omitted from analysis, report if this was due to attrition or intentional exclusion and provide justification.	Yes	Inclusion criteria was described in the Materials and methods
For every figure, are **statistical tests** justified as appropriate? Do the data meet the assumptions of the tests (e.g., normal distribution)? Describe any methods used to assess it. Is there an estimate of variation within each group of data? Is the variance similar between the groups that are being statistically compared?	Yes	Information provided in the Figure legends and Materials and Methods

Sample definition and in-laboratory replication	Information included in the manuscript?	In which section is the information available? (Reagents and Tools Table, Materials and Methods, Figures, Data Availability Section)
In the figure legends: state number of times the experiment was **replicated** in laboratory.	Yes	Figure legends
In the figure legends: define whether data describe **technical or biological replicates**.	Yes	Figure legends

Ethics

Ethics	Information included in the manuscript?	In which section is the information available? (Reagents and Tools Table, Materials and Methods, Figures, Data Availability Section)
Studies involving **human participants**: State details of **authority granting ethics approval** (IRB or equivalent committee(s), provide reference number for approval.	Yes	Materials and Methods
Studies involving **human participants**: Include a statement confirming that **informed consent** was obtained from all subjects and that the experiments conformed to the principles set out in the WMA Declaration of Helsinki and the Department of Health and Human Services Belmont Report.	Yes	Materials and Methods
Studies involving **human participants: For** publication of **patient photos**, include a statement confirming that consent to publish was obtained.	Not Applicable	
Studies involving experimental **animals**: State details of **authority granting ethics approval** (IRB or equivalent committee(s), provide reference number for approval. Include a statement of compliance with ethical regulations.	Not Applicable	
Studies involving **specimen and field samples:** State if relevant **permits** obtained, provide details of authority approving study; if none were required, explain why.	Not Applicable	

Dual Use Research of Concern (DURC)	Information included in the manuscript?	In which section is the information available? (Reagents and Tools Table, Materials and Methods, Figures, Data Availability Section)
Could your study fall under dual use research restrictions? Please check biosecurity documents and list of **select agents and toxins** (CDC): https://www.selectagents.gov/sat/list.htm	Not Applicable	
If you used a select agent, is the security level of the lab appropriate and reported in the manuscript?	Not Applicable	
If a study is subject to dual use research of concern regulations, is the name of the **authority granting approval and reference number** for the regulatory approval provided in the manuscript?	Not Applicable	

Reporting

The MDAR framework recommends adoption of discipline-specific guidelines, established and endorsed through community initiatives. Journals have their own policy about requiring specific guidelines and recommendations to complement MDAR.

Adherence to community standards	Information included in the manuscript?	In which section is the information available? (Reagents and Tools Table, Materials and Methods, Figures, Data Availability Section)
State if relevant guidelines or checklists (e.g., **ICMJE, MIBBI, ARRIVE, PRISMA**) have been followed or provided.	Not Applicable	
For **tumor marker prognostic studies,** we recommend that you follow the **REMARK** reporting guidelines (see link list at top right). See author guidelines, under 'Reporting Guidelines'. Please confirm you have followed these guidelines.	Not Applicable	
For **phase II and III randomized controlled trials**, please refer to the **CONSORT** flow diagram (see link list at top right) and submit the CONSORT checklist (see link list at top right) with your submission. See author guidelines, under 'Reporting Guidelines'. Please confirm you have submitted this list.	Not Applicable	

Data Availability

Data availability	Information included in the manuscript?	In which section is the information available? (Reagents and Tools Table, Materials and Methods, Figures, Data Availability Section)
Have **primary datasets** been deposited according to the journal's guidelines (see 'Data Deposition' section) and the respective accession numbers provided in the Data Availability Section?	Not Applicable	
Were **human clinical and genomic datasets** deposited in a public access-controlled repository in accordance to ethical obligations to the patients and to the applicable consent agreement?	Not Applicable	
Are **computational models** that are central and integral to a study available without restrictions in a machine-readable form? Were the relevant accession numbers or links provided?	Not Applicable	
If publicly available data were reused, provide the respective **data citations in the reference list**.	Yes	Accession number of the publicly available data was provided in the methods. Citation of the original dataset (Marakalala et al, Nat. Med. 2016) is also in the citation list
